# Phenotypic plasticity in maize grain yield: Genetic and environmental insights of response to environmental gradients

**DOI:** 10.1002/tpg2.70078

**Published:** 2025-08-07

**Authors:** Fatma Ozair, Alper Adak, Seth C. Murray, Ryan T. Alpers, Alejandro C. Aviles, Dayane C. Lima, Jode Edwards, David Ertl, Michael A. Gore, Candice N. Hirsch, Joseph E. Knoll, James C. Schnable, Maninder P. Singh, Erin E. Sparks, Addie Thompson, Teclemariam Weldekidan, Wenwei Xu

**Affiliations:** ^1^ Interdisciplinary Graduate Program in Genetics and Genomics (Department of Biochemistry and Biophysics) Texas A&M University College Station Texas USA; ^2^ Department of Soil and Crop Sciences Texas A&M University College Station Texas USA; ^3^ Department of Plant and Agroecosystem Sciences University of Wisconsin–Madison Madison Wisconsin USA; ^4^ Bayer Crop Science St. Louis Missouri USA; ^5^ USDA‐ARS Corn Insect and Crop Genetics Research Unit Ames Iowa USA; ^6^ Iowa Corn Promotion Board Johnston Iowa USA; ^7^ Plant Breeding and Genetics Section, School of Integrative Plant Science Cornell University Ithaca New York USA; ^8^ Department of Agronomy and Plant Genetics University of Minnesota St. Paul Minnesota USA; ^9^ USDA‐ARS Crop Genetics and Breeding Research Unit Tifton Georgia USA; ^10^ Department of Agronomy and Horticulture University of Nebraska–Lincoln Lincoln Nebraska USA; ^11^ Department of Plant, Soil and Microbial Sciences Michigan State University East Lansing Michigan USA; ^12^ Department of Plant and Soil Sciences University of Delaware Newark Delaware USA; ^13^ Texas A&M AgriLife Research Texas A&M University System Lubbock Texas USA

## Abstract

Understanding genotype‐by‐environment (G × E) interactions that underlie phenotypic variation, when observed for complex traits in multi‐environment trials, is important for biological discovery and for crop improvement. The regression‐on‐the‐mean model is an approach to observe G × E trends for complex traits across a gradient of environmental inputs. Biologically relevant environmental index values can be utilized to quantify phenotypic plasticity of individuals by correlating environmental means and environmental parameters within specific time windows. By accounting for trait stability, improvements can be made in genome‐wide association studies and genomic prediction models involving data with high volumes of environments, genotypes, and their interaction effects. Here, field data collected through the national hybrid maize (*Zea mays* L.) Genomes‐to‐Fields project was analyzed. Reaction norm parameters were obtained from photothermal ratio (PTR) indices for hybrid grain yield (GY) using three separate tester populations across 29 diverse environments. The PTR time windows most correlated with the average GY were discovered to differ by tester but were confounded by region. Using 100,000 single‐nucleotide polymorphisms (SNPs), we discovered 96 quantitative trait loci (QTLs) significantly associated with GY and six QTLs significantly associated with GY stability. The modified, regression‐on‐the‐mean genomic prediction model using PTR‐estimated reaction norm parameters of each hybrid worked nearly as well as a traditional, additive genomic prediction model using the G × E interaction terms but took 192× less time. The PTR genomic prediction model predicted untested environment performance (0.57–0.71) better than untested hybrid performance (0.26–0.37). This study suggests improved potential for multi‐environment genomic predictions by incorporating environmental measures to dissect the complexities of differential performance of genotypes across environments.

## Core Ideas

- Developmentally sensitive timepoints for grain yield were revealed by temporal photothermal ratio (PTR) data.
- Hybrid grain yield plasticity was quantified by modeling genotype‐by‐environment (G × E) interactions with a PTR index.
- PTR‐derived values allowed for hybrid genomic prediction scenarios for grain yield without prior performance data.
- The regression‐on‐the‐mean model had comparable GY prediction ability to the additive model but with reduced computational burden.
- Loci and candidate genes accounting for grain yield plasticity were identified by PTR‐derived data.

**Abbreviations**
BLUP: best linear unbiased predictor
G × E: genotype‐by‐environment
G2F: Genomes‐to‐Fields
GDD: growing degree days
GWAS: genome‐wide association studies
GY: grain yield
MAGIC: multiparent advanced generation intercross
PTR: photothermal ratio
QTL: quantitative trait locus
SNP: single‐nucleotide polymorphism

## 1 INTRODUCTION

The phenotypic development and growth of crops are largely influenced by the environmental conditions they are exposed to during their life cycle. Crops, being stationary sessile species, evolved to adapt to their local surroundings by sensing environmental stimuli and producing biological responses best suited for their survival. (Pierik & Testerink, 2014). Genotype‐by‐environment (G × E) interactions and phenotypic plasticity are key concepts in the study of crop adaptation and resilience. The G × E interaction refers to the differential performance of genotypes across various environmental conditions, indicating that the success of a particular genetic makeup is highly dependent on the environment in which it is placed. Phenotypic plasticity, on the other hand, is the ability of a single genotype to produce different phenotypes in response to changing environmental conditions, thus playing a crucial role in how crops adapt to various stresses and climatic variations. The critical role of G × E interactions and phenotypic plasticity has been shown in the adaptation, resilience, and yield stability of crops. Understanding these dynamics is vital for genetics, crop improvement, and plant breeding programs to effectively respond to the challenges posed by climate change and environmental variability. The collection of all abiotic and biotic factors imposed on a crop throughout growth directly contributes to the trends observed for G × E and phenotypic plasticity; therefore, it is crucial to consider a multitude of genotypes and environments to uncover these patterns. One approach to quantify phenotypic plasticity is by examining the reaction norms of genotypes, which are graphical representations showing the range of phenotypes expressed by a single genotype across a gradient of environmental conditions (Fu & Wang, 2023; Guo et al., 2020; Li et al., 2018). The Finlay–Wilkinson method for regression analysis of phenotypes, also known as the regression‐on‐the‐mean model, has been widely used as an approach to evaluate trait stability by regressing the trait value of individual genotypes against an index of the mean performance of a trait across several environments (Finlay & Wilkinson, 1963). The slope of a genotype's reaction norm is particularly insightful because it provides a measure of the degree of plasticity: Steeper slopes indicate greater plasticity, as the phenotype changes more markedly with environmental variation. Various methods of defining a continuous, environmental component to incorporate into reaction norms have been proposed to better understand underlying patterns of G × E in crop species (Choquette et al., 2023; de los Campos et al., 2020; Jarquín et al., 2017).

Maize (*Zea mays* L.) is among the most notable model species studied for phenotypic plasticity due to its importance as a common crop grown across diverse environments (Kusmec et al., 2017; Oostra et al., 2018). Historical cultivation of maize landraces adapted to different geographic regions, beyond its center of origin in Central America, demonstrates adaptation potential while understanding the genetic and phenotypic variability of populations (Arteaga et al., 2016; Prasanna, 2012; Rivas et al., 2022). Research on phenotypic plasticity and reaction norms in maize have significantly advanced our understanding of how crops can be bred and managed for better performance under diverse conditions. For instance, studying the effect of artificial selection on phenotypic plasticity in maize highlighted the importance of the genetic basis of G × E interactions to identify adaptive limitations in modern germplasm suited to temperate North American regions (Gage et al., 2017)​. Their approach used genome‐wide association studies (GWAS) to explore the genomic locations associated with these interactions, providing insights into the regions of the genome involved in phenotypic plasticity. While plant breeders and researchers are aware that maize traits display variable sensitivity to different environmental parameters, the underlying physiology and alleles causing these differences remain largely unknown and untested across diverse environments for agronomic traits of interest. Many environments are needed to dissect the various environmental parameters influencing maize traits at the gene and genotype levels.

A separate study in maize, focusing on quantifying phenotypic plasticity to improve maize yields, emphasized the complexity of breeding decisions due to the intricate relationship between yield, plastic responses to multiple stresses, and yield‐related genes (Kusmec et al., 2018)​​. The findings suggested that identifying loci conferring plasticity or stability from exotic germplasm is essential for maximizing future adaptation and yield of maize hybrids. Elite maize lines selected in optimal US Midwest environments and cultivation for specific traits have diminished the genetic diversity of hybrids throughout successive generations of breeding (Gerke et al., 2015; Khoury et al., 2021). The introgression of rare, favorable alleles from exotic germplasm unlocks the potential to improve traits influenced by G × E and plasticity through the selection process (Wang et al., 2017).

**Core Ideas**

- Developmentally sensitive timepoints for grain yield were revealed by temporal photothermal ratio (PTR) data.
- Hybrid grain yield plasticity was quantified by modeling genotype‐by‐environment (G × E) interactions with a PTR index.
- PTR‐derived values allowed for hybrid genomic prediction scenarios for grain yield without prior performance data.
- The regression‐on‐the‐mean model had comparable GY prediction ability to the additive model but with reduced computational burden.
- Loci and candidate genes accounting for grain yield plasticity were identified by PTR‐derived data.

Phenotypic plasticity's contribution to maize adaptation and heterosis further demonstrates the significance of understanding underlying genetic architecture (N. Liu et al., 2020). By modeling genotype and environment interactions, valuable insights have been made into the effects of specific genetic loci on adaptation‐related traits and heterosis, underlining the importance of quantitative trait loci (QTLs) or quantitative trait nucleotides effects across different environments. Optimization of hybrids tolerating specific environmental stresses is possible in maize by leveraging heterosis in addition to the selection for additive genetic trait plasticity.

A complex genetic architecture underlying the plasticity of maize agronomic traits has revealed a dynamic interplay between genetic diversity, environmental variation, and their interactions​​ (Jin et al., 2023). For example, altitude‐related environmental factors were found to be drivers for variation in flowering time but not in plant architecture and yield traits, underscoring the importance of site‐specific breeding strategies for local adaptation for various traits.

The genetic interconnection between, for instance, yield and yield stability is a cornerstone of crop breeding and agronomy, demonstrating the intricate balance between local genetic selection and environmental adaptability. This linkage suggests that the successful selection of crops for high yield does not come synergistically with or at the expense of yield stability, but rather, these two crucial traits can be improved concurrently (Tollenaar & Lee, 2002).

Coordinated research initiatives across numerous broad environments, like the Genomes‐to‐Fields (G2F) G × E project (https://www.genomes2fields.org/), are needed to further explore and validate the genetic relationships between yield and yield stability across a wide range of environments. The G2F G × E project from 2014 to 2023 has trialed approximately 180,000 field plots involving more than 2500 maize hybrids across 162 unique environments in North America, the largest public sector cooperative project of its type. The hybrids tested, however, have changed across the years (Lima, Washburn, et al., 2023).

To understand the genetic basis of the interplay between G × E and phenotypic plasticity, G2F datasets on maize tester hybrids across 29 environments in 2020 and 2021 were analyzed because these years used the same hybrids. Comprehensive insight into the genetic architecture of phenotypic plasticity was investigated for grain yield (GY). This study aimed to (i) observe trends in the variation of GY across multiple environments with diverse gradients, (ii) determine environmental index values for reaction norm modeling using the mean values of a weather parameter across time windows that are highly correlated with the mean GY across all environments, (iii) examine the reaction norm for GY using parameters (e.g., slope, intercept) of the linear model by fitting the individual hybrids’ performances to the environmental index, (iv) identify QTL and candidate genes associated with GY and its stability, and (v) assess the findings of this study by comparing the metrics of different G × E genomic prediction models.

## 2 MATERIALS AND METHODS

### 2.1 Plant material and growing environments

**FIGURE 1:**
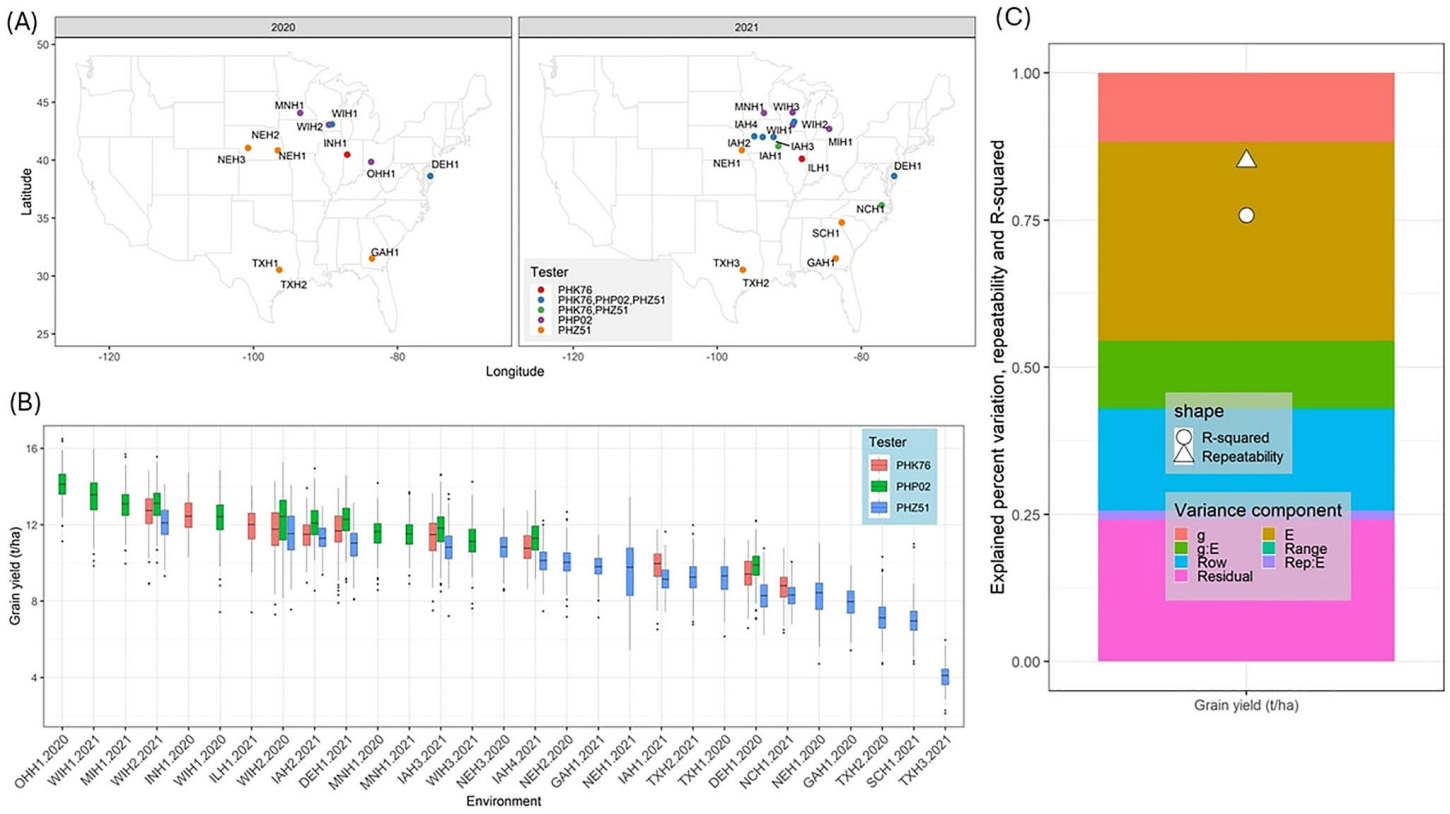
(A) Data on Genomes‐to‐Fields (G2F) growing environments during 2020 and 2021, color‐coded by tester information for cultivated hybrids. (B) The arrangement of grain yield values for hybrids in ascending order. (C) Relative contribution of variances for grain yield (measured in t/ha). The variance of range effects was too small to be shown in the figure.

In this study, the 2020 and 2021 G2F project's maize hybrids, detailed in Lima, Aviles, et al. (2023), were planted in 29 environments from a combination of year:location. A subset of 220 inbreds, selected from 779 doubled haploid inbreds derived from the Stiff Stalk MAGIC (multiparent advanced generation intercross) population (Michel et al., 2022), were used to advance hybrids grown across G2F environments in 2020 and 2021. These 220 inbreds were common across each of the three hybrid populations, which were created using three non‐Stiff Stalk expired plant variety protection tester lines (PHK76 [Pioneer Hi‐Bred International, Inc., 1988], PHP02 [Pioneer Hi‐Bred International, Inc., 1989], and PHZ51 [Pioneer Hi‐Bred International, Inc., 1987]). The three hybrid populations with their tester and environment information are given in Figure 1A.

Measurements for GY were recorded in each environment based on standard operating procedures (SOP). SOP details were determined within the G2F project guidelines (https://www.genomes2fields.org/resources/). In general, PHZ51 hybrids are adapted to southern regions, PHP02 to northern regions, and PHK76 to intermediate regions in the Midwest (Figure 1A). PHZ51, PHP02, and PHK76 were represented in 20, 14, and 11 environments, respectively. Overall, six locations (seven environments) featured all three tester populations together, such as Iowa and Delaware, and two locations featured two tester populations together. There were a total of 45 tester:environment combinations.

### 2.2 Best linear unbiased predictors (BLUPs) for GY

The linear model fit to the data was of the following form: $\mathrm{Grain}\;\mathrm{Yiel}d_{ijkl}=\mu +{\mathrm{Genotype}}_{i}+{\mathrm{Environment}}_{j}+{\;\mathrm{GE}}_{ij}+{\mathrm{Range}}_{k}+{\mathrm{Row}}_{l}+\mathrm{Rep}{\left[E\right]}_{kj}+\varepsilon_{ijkl}$, where $\mathrm{Grain}{\mathrm{Yield}}_{ijkl}$ represents the phenotypic data of GY (t/ha) for the *i*th hybrid in the *j*th environment, *k*th range, and *l*th row, *μ* is the grand mean of the trait, ${\mathrm{Genotype}}_{i}\frac{iid}{∼}N\left(0,\sigma_{G_{i}}^{2}\right)$ is the random effect of hybrid *i*, ${\mathrm{Environment}}_{j}\frac{iid}{∼}N\left(0,\sigma_{E_{j}}^{2}\right)$ is the random effect of environment *j*, ${\mathrm{GE}}_{ij}\frac{iid}{∼}N\left(0,\sigma_{{\mathrm{GE}}_{ij}}^{2}\right)$ is the random effect of interactions between hybrid *i* and environment *j*, ${\mathrm{Range}}_{k}\frac{iid}{∼}N\left(0,\sigma_{{\mathrm{Range}}_{k}}^{2}\right)$ and ${\mathrm{Row}}_{l}\frac{iid}{∼}N\left(0,\sigma_{{\mathrm{Row}}_{l}}^{2}\right)$ are the horizontal and lateral grids of plots within each environment (rows are parallel to planted rows and ranges are perpendicular), $\mathrm{Rep}{\left[E\right]}_{kj}\frac{iid}{∼}N\left(0,\sigma_{\mathrm{Rep}{\left[E\right]}_{kj}}^{2}\right)$ is the replication effects found in each environment since each environment contains two replications, and $\varepsilon_{ijkl}\frac{iid}{∼}N\left(0,\sigma_{\varepsilon_{ijkl}}^{2}\right)$ refers to the residual error. All model effects except the mean, *μ*, were considered random. Variance components were estimated from the full dataset with restricted maximum likelihood using the lmer() function contained in the lme4 R package (Bates et al., 2015). The heritability for GY was calculated as follows:


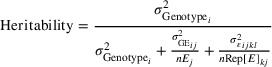

where $\sigma_{i}^{2}$, $\sigma_{ij}^{2}$, and $\sigma_{\varepsilon_{ijkl}^{2}}$ are the variance components of the overall genotype effects, the interaction between genotype and environment, and the error term, respectively. BLUP values for GY were obtained from the combined effects of genotypes, environments, and their interaction (G × E). The BLUP values were retained for the subsequent genomic prediction and reaction norm calculations.

### 2.3 Environmental index

The EnvRtype package in R was implemented to obtain daily weather data derived from the NASA Prediction of Worldwide Energy Resources (POWER) project (Costa‐Neto et al., 2021). The environmental index was calculated based on the Pearson correlations between the photothermal ratio (PTR) and the mean value of the G × E BLUPs for GY across the growing environments separated by the three hybrid populations. The PTR is an environmental parameter that represents the ratio of day length (hour) divided by growing degree days (GDD) (Li et al., 2021; B. Liu & Heins, 2002). The GDD was calculated based on $\left(\frac{T_{\max}-T_{\min}}{2}\right)-T_{\mathrm{base}}$, where $T_{\max}$ and $T_{\min}$ are the maximum and minimum temperatures (°C), respectively, recorded without thresholding, and $T_{\mathrm{base}}$ is the base temperature, set as 10°C (Costa‐Neto et al., 2021; Soltani & Sinclair, 2012). Mean PTR values were calculated for windows of days of all sizes from a window of 1 day to 120 days. For each window size, average PTR values were calculated for all possible windows from 1 day after planting in 1‐day increments. For example, for a 5‐day window, the mean PTR value was calculated from Day 1 to 5, Day 2 to 6, …, up to Day 116 to 120 after planting. Subsequently, the mean PTR value from the window that demonstrated the highest correlation to the yield estimates in each environment was utilized as the environmental index for reaction norms.

### 2.4 Stability analysis

The stability of GY was calculated based on the linear fit of scaled and centered GY of hybrids across the environmental index of environments. Then, slope and intercept were retrieved for each hybrid and used as a merit of stability and performance (Sjoberg et al., 2020) using regression where the G × E BLUPs were set as the dependent variable and the PTR index values were set as the independent variable. The stability can be classified according to the following slope values: slope < 1 where the performance was higher than expected in unfavorable environments compared to favorable environments based on the population average performance, slope = 0 where the performance was consistent across unfavorable and favorable environments, slope = 1 where the performance was consistent across environmental means, and slope > 1 where the performance was higher than expected in favorable environments compared to unfavorable environments based on the population average performance (Bernardo, 2002).

### 2.5 QTLs analysis

Genomic data from the 220 inbreds in 2020 and 2021 obtained from CyVerse (https://doi.org/10.25739/hzzs‐a865 and https://doi.org/10.25739/5ae3‐sw62) were employed for QTL mapping. A total of 100,000 single‐nucleotide polymorphisms (SNPs) were selected, evenly distributed across 10 chromosomes. Further details related to genomic data can be found in Michel et al. (2022). Three types of phenotypes were used for dissecting GY of the three hybrid populations: (i) the mean GY of the hybrids, (ii) the slope value of the hybrids, and (iii) the intercept values of the hybrids. QTL mapping was conducted using R/qtl2, where the population type was set as “dh6,” in accordance with the development design of the inbreds of the six‐way doubled haploid Wisconsin MAGIC population (Broman et al., 2019). R/qtl2::scan1perm() function was used to calculate the threshold for significant QTLs using 1000 permutations, and resulted in a limit of detection threshold of five to identify significant regions. The candidate genes within these intervals were identified using the Zm‐B73‐NAM‐5.0 reference genome on MaizeGDB (https://jbrowse.maizegdb.org/).

### 2.6 Environmental index facilitated genomic prediction

An environmental index facilitated genomic prediction was conducted to predict the GY, validated using four common prediction scenarios related to a plant breeding program (Jarquín et al., 2017). Bayesian generalized kernel regression genomic prediction models were applied to the three different hybrid populations separately using reproducing kernel Hilbert spaces from the BGLR package (Perez & de los Campos, 2014). The steps were as follows: (i) 220 hybrids were partitioned into tested and untested groups with percent ratios of approximately 70% and 30%, respectively. (ii) Environments were similarly separated into tested and untested categories using the leave‐one‐out method, where one environment was removed for the next step, designated as the untested environment, while the others were termed as tested environments. (iii) A linear fit was applied for each tested hybrid using their GY data and the environmental index (see Section 2.3) of the tested environments, followed by calculating the slope and intercept values. (iv) The genomic prediction model was trained using the slope and intercept values of the tested hybrids in the tested environments. (v) The slope and intercept values of both untested and tested hybrids were predicted using a genomic relationship matrix derived from 100,000 genomic markers using ridge regression (rrBLUP package) (Endelman, 2011). The markers with a minor allele frequency less than 0.05 were excluded, and missing markers were imputed using expectation maximization. (vi) GY values of both untested and tested hybrids were calculated using their predicted slope and intercept values, along with the genomic relationship matrix estimated from the 100,000 SNPs. The two models assessed for prediction accuracy were based on (Malosetti, 2013):

*Model 1 (M1)*: Additive effects model

$$Y_{ij}=\mu +G_{i}+E_{j}+GE_{ij}+\varepsilon_{ij}$$

where $Y_{ij}$ is the GY of the *i*th hybrid in the *j*th environment, μ is the overall mean, $G_{i}\;$ is the genotype of the *i*th hybrid, $E_{j}\;$ is the *j*th environment, $GE_{ij}$ is the G × E of the *i*th hybrid in the *j*th environment, and $\varepsilon_{ij}$ is the residual error.

*Model 2 (M2)*: Regression on the mean model

$$Y_{ij}=\mu +G_{i}+E_{j}+b_{i}E_{j}+\varepsilon_{ij}$$

M2 retains factors from the M1 model, except $GE_{ij}\;$ is replaced by $b_{i}E_{j}$, which is the *i*th hybrid's specific reaction norm parameter in the *j*th environment. Both models were run using Texas A&M's High Performance Research Computer.

Subsequently, four types of cross‐validation schemes between the actual GY and the predicted GY were calculated for tested hybrids in tested environments, untested hybrids in tested environments, tested hybrids in untested environments, and untested hybrids in untested environments. This procedure was iterated 100 times for each cross‐validation scenario using different hybrids and environments summarized as the mean estimates and confidence intervals for each hybrid and environment for each set of testers.

## 3 RESULTS

### 3.1 Sources of variation for GY

The variance component estimates for GY in Figure 1C revealed that the environment explained the highest variation (∼30%), which aligns with the amount of diverse environments included in the study. The G × E effect explained about 12% of the variation in GY. The effects of G × E on GY were further explored after confirming its contribution to phenotypic variation across the hybrids. The moderate model *R* ^2^ value of ∼0.75 is likely reduced by the high contribution of unexplained variation in the residual error. The environments in which all three populations were planted consistently had lower BLUPs for PHZ51 and higher BLUPs for PHP02 (Figure 1B). The southern environments using PHZ51, which were distributed over the widest geographic locations, tended to have the lowest BLUP values overall.

### 3.2 Identifying the environmental index linked to GY

**FIGURE 2:**
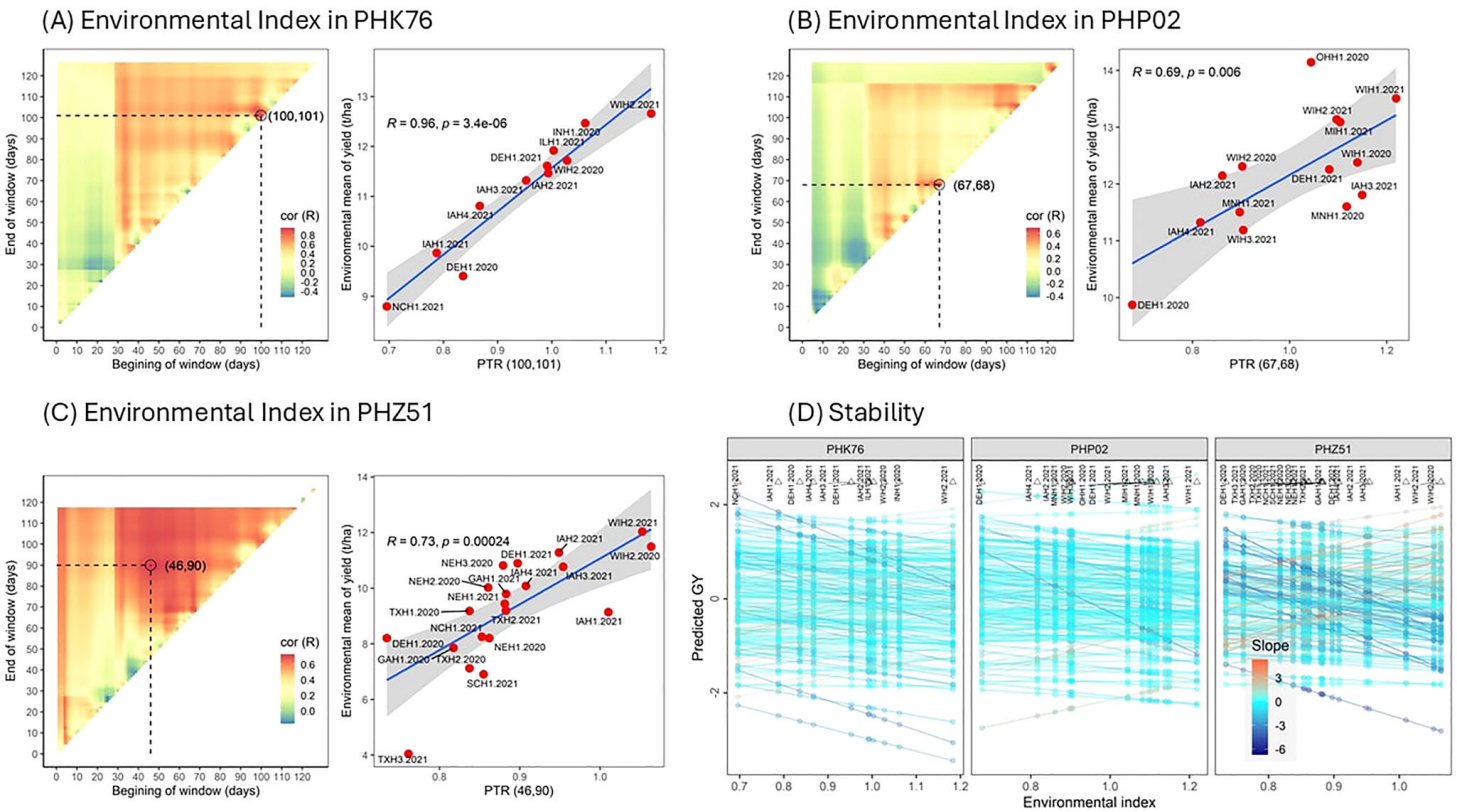
Environmental index using sliding window correlation between photothermal ratio (PTR) and average grain yield (t/ha) across environments for the three hybrid populations and their stability. Figure Panels (A) (PHK76), (B) (PHP02), and (C) (PHZ51) show the correlations between values of all possible windows of PTR with the grain yield (left), highlighting the highest correlations between specific windows and the mean grain yield across environments in hybrid populations. (D) Predicted grain yield (predicted GY, on the *y*‐axis) calculated by linearly regressing the scaled and centered grain yield of each hybrid on the environmental index (on the *x*‐axis) across each growing environment of three hybrid populations. Points are the predicted grain yield values. Each line, representing a hybrid, was colored based on the slope parameter of the linear fit to visualize the reaction norms of the hybrids.

Based on the sliding window correlation analysis between PTR and BLUPs of GY from each environment, specific windows were identified that showed the strongest correlations with GY separately across the three maize hybrid populations (PHK76, PHP02, and PHZ51) and across all tested environment (Figure 2A–C). In the case of PHK76, the greatest correlation was observed in the 2‐day window between 100 and 101 days (Figure 2A). For PHP02, the highest correlation was found in the 2‐day window of 67–68 days (Figure 2B). In the case of PHZ51, the most significant correlation spanned a broader period, specifically the 45‐day window from Day 46 to Day 90 (Figure 2C). The mean PTR value in these specific windows was designated as the environmental index. Later, the stability of hybrid GY was analyzed using linear fits built between the environmental index and GY (centered and scaled) of each environment, revealing different reaction norms of hybrids (Figure 2D).

### 3.3 QTLs linked to GY and its stability

**FIGURE 3:**
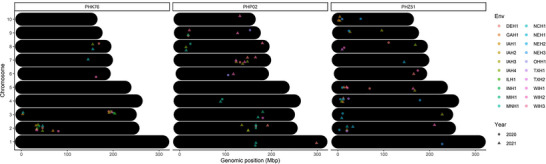
Quantitative trait loci (QTLs) linked to grain yield in three different hybrid populations advanced by PHK76, PHP02, and PHZ51 testers across various growing environments and years.

Several QTLs were discovered for GY across different environments and years, depending on the hybrid populations (Figure 3); their corresponding candidate genes are provided in Table S1. A total of 96 unique QTLs were significantly associated with GY, with 24 belonging to PHK76, 34 to PHP02, and 38 to PHZ51. As most QTLs were unique to environments and testers, the PHZ51 population had the most QTLs across the chromosomes, likely due to this population being planted in the most environments compared to the other populations.

**FIGURE 4:**
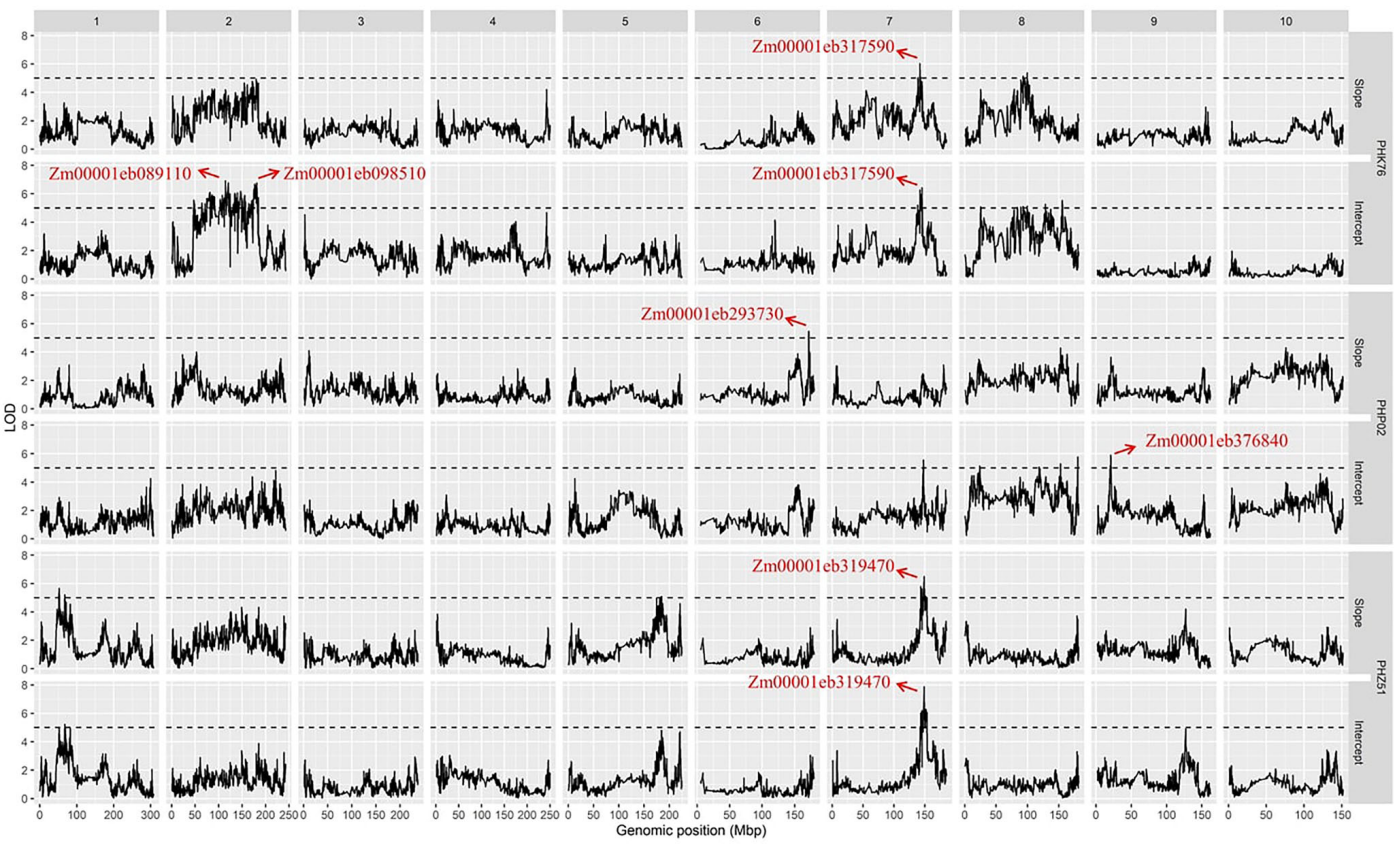
Quantitative trait loci (QTLs) identified for slope and intercept of hybrids across three testers. Testers for each population are provided on the right. A limit of detection (LOD) threshold of five was used for significance.

The merit of GY stability across locations and years (29 environments) was quantified by slope and intercept values. Among the QTLs discovered, six relevant QTLs were identified related to GY stability as shown in Figure 4 and Table S2. Two of these loci were detected for both slope and intercept (PHK76 Chr 7 and PHZ51 Chr 7). The detected positions of these Chr 7 loci between the two testers were 6.7 Mb apart. The function of potential candidate genes related to GY stability for three hybrid populations is also given in Table 1. In total, six candidate genes related to GY stability were provided across 29 environments and three hybrid populations.

**TABLE 1:** Quantitative trait loci (QTLs) discovered along with candidate genes and their functions for grain yield, slope, and intercept for three different maize populations with PHK76, PHP02 and PHZ51 testers.

Population	Trait	SNP position (Mbp)	Chr	No. genes in interval	Likely candidate gene	Function
PHK76	Slope	142217610–142236552	7	11	*Zm00001eb317590*	E3 ubiquitin‐protein ligase RFWD2 (RING/U‐box superfamily protein) (ubiquitin‐protein ligase/zinc ion‐binding protein)
PHK76	Intercept	114599617–114602053	2	41	*Zm00001eb089110*	Alpha‐humulene/(−)‐(E)‐beta‐caryophyllene synthase
PHK76	Intercept	181334786–181336694	2	50	*Zm00001eb098510*	60S ribosomal protein L33‐B (60S ribosomal protein L35a‐2)
PHK76	Intercept	142217610–142236552	7	5	*Zm00001eb317590*	E3 ubiquitin‐protein ligase RFWD2 (RING/U‐box superfamily protein) (ubiquitin‐protein ligase/zinc ion‐binding protein)
PHP02	Slope	171965158–171966322	6	1	*Zm00001eb293730*	Uncharacterized protein
PHP02	Intercept	21039872–21045940	9	15	*Zm00001eb376840*	NB‐ARC domain‐containing protein
PHZ51	Slope	148935616–148937490	7	69	*Zm00001eb319470*	Auxin‐responsive protein (IAA33–Aux/IAA‐transcription factor 33)
PHZ51	Intercept	148935616–148937490	7	84	*Zm00001eb319470*	Auxin‐responsive protein (IAA33–Aux/IAA‐transcription factor 33)

Abbreviation: SNP, single‐nucleotide polymorphism.

### 3.4 Environmental index‐based genomic prediction for GY

**FIGURE 5:**
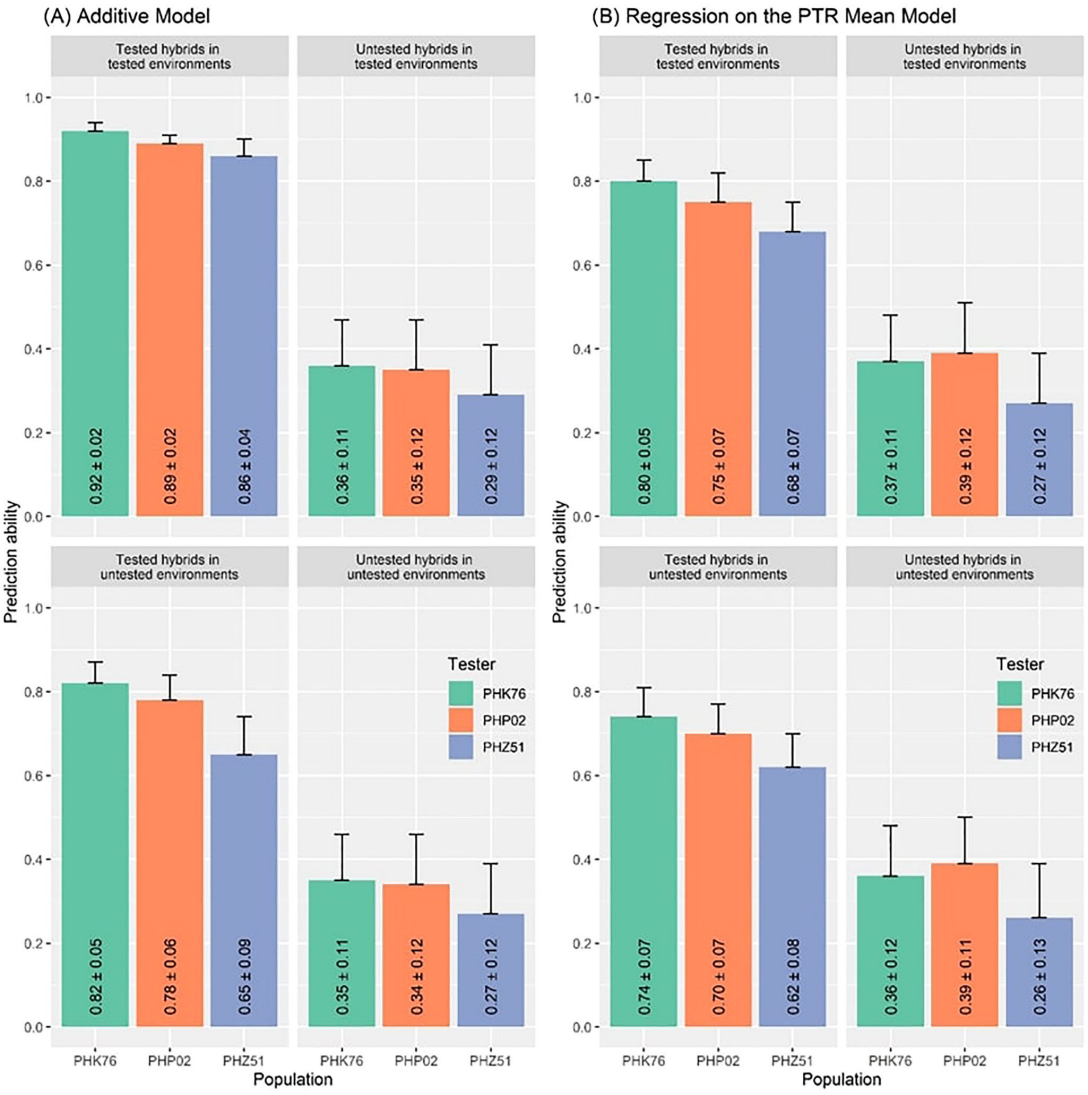
Comparison of two models for genomic prediction response. (A) Genomic prediction of grain yield facilitated using M1 (additive model with genotype‐by‐environment [G × E]) for four prediction scenarios illustrated by each facet and three hybrid populations illustrated by legend. The standard error is represented by the vertical lines above the bars. (B) Genomic prediction of grain yield facilitated using M2 (regression on the photothermal ratio [PTR] mean model).

A specific PTR window differentiated the environments for the three hybrid populations. The reaction norm of GY was then revealed as a response to day length and GDD, thanks to the linear regression of the hybrids' GY on the specific window of PTR across distinct environments (Figure 2D). Slope and intercept are the merit of the plasticity of each hybrid in two aspects: (i) slope is the identification of which hybrids are more or less favorable across varying environmental conditions and (ii) intercept is the identification of hybrids’ baseline yield potential. Environmental index facilitated genomic prediction provided different prediction abilities with small differences between PHK76, PHP02, and PHZ51 testers grown across 11, 14, and 20 environments (Figure 5). Both the M1 (additive effects model, with G × E interaction effects) and M2 (regression on the PTR mean model) performed similarly in most cases. M1 was better on tested hybrids in tested environments across all three tester populations. For both models, in tested hybrids in untested environments, the prediction ability was between ∼0.6 and 0.8 depending on the hybrid population, and M1 performed better in all cases. In untested hybrids in tested environments, the prediction ability dropped to ∼0.3–0.4, and M1 was numerically better for two of three testers. The most challenging prediction scenario, untested hybrids in untested environments, provided prediction abilities ranging between ∼0.2 and 0.4, and the M1 model performed numerically better for two of three testers.

### 3.5 Computational costs between the M1 and M2 models

Although both of the genomic models incorporated G × E effects to predict GY, the computational burden with the M2 model was greatly reduced in comparison to the M1 model. Using the same computing infrastructure, the M1 model took 192× longer to run (24 days vs. 3 h). The M2 relies on G × E inputs derived from the slope and intercept values of reaction norms; thus, it consists of a matrix with the same amount of observations (rows) as the number of genotypes. On the other hand, the M1 model relies on fitting all the combinations of genotypes by environment, thus greatly increasing the number of observations as the amount of genotypes/environments increases. As shown in Figure 5, both models give comparable prediction accuracies. Therefore, the M2 model is preferable when using an optimal environmental parameter, datasets are large, and run time is important in decision‐making.

## 4 DISCUSSION

Overall, this MAGIC‐derived hybrid population was grown in a high number of environments and uniquely used multiple tester lines to ensure adaptation of the tested hybrids was relevant to the target environments. GY had moderately high heritability, in part due to the fact these were doubled haploid lines that were not pre‐selected for high GY. This high heritability allowed for the dissection of environmental factors and the genetic mapping of numerous loci.

### 4.1 Identifying the environmental index linked to GY

The hybrids used in the G2F project data demonstrated substantial G × E interactions and phenotypic plasticity. Maize's adaptability and stability across diverse environmental conditions are crucial for maximizing GY over many diverse target environments and over unpredictable weather. This study uniquely contributes to the understanding of GY in maize by identifying specific environmental windows that correlated with yield variation across diverse environments. This highlights the significance of the temporal dimensions of environmental factors in maize development and GY, and the interaction with genetic factors. Identifying timepoints with the greatest effects on G × E interactions is useful to understand when to measure physiological aspects of the crop. Like this study, prior research has shown that maize and sorghum (*Sorghum bicolor* (L.) Moench) exhibit significant phenotypic plasticity in response to environmental stimuli (Jin et al., 2023; Kusmec et al., 2017; Li et al., 2018; Millet et al., 2019; Mu et al., 2022). Photoperiodicity as well as heat sensitivity can affect crucial stages of vegetative and flowering development that ultimately affect GY (Bonhomme et al., 1994; Lobell et al., 2013; Wang et al., 2010). Therefore, differentiating populations based on a specific environmental index that is biologically meaningful and variable can help to dissect the target environment G × E patterns at play during crucial developmental stages when assessing the performance of hybrids. Incorporation of environmental data in the form of numerically ordered reaction norms along with phenotypic data may also reveal the underlying variation among hybrids based on their sensitivity to local conditions (Piepho & Blancon, 2023). Each tester used for hybrid formation had unique genetic makeups that showed clear differences in the window sizes for PTR correlations to environmental mean yields. This was partially expected considering the range of environmental locations where the hybrids were planted and the inherent adaptations of the individual testers. However, the dramatic differences observed in window sizes across the populations, as early as 45 days for hybrids with PHZ51 testers and 100–101 days for hybrids with PHK76 testers, were surprising. Plotting each environment's weather parameters over time, we did not observe any distinct spikes or obvious causes for these specific windows, or the differences between them. These varying windows may be due to genetic differences, environmental factors, or their interactions, which we could not separate since these testers were confounded with tested regions in all but two locations. Among environmental factors, the day length affecting photoperiod and the confounding associations with temperature had a pronounced difference depending on geographic latitudes. Southern locations tend to have longer day lengths than the northern regions early in the year, but the opposite is true in summer. This, combined with differences in planting dates, results in southern oddities like Texas (a southern location), flowering as the days are getting longer. In contrast, hybrids in most US maize production and G2F locations flower as the days are getting shorter. The southern conditions for both photoperiod and temperature are not particularly suitable for the temperate‐adapted inbreds of the Wisconsin MAGIC population. This was intentionally modulated through tester choice in hybrids, which allowed hybrids with the same population of lines to be appropriately evaluated in all locations. Thus, an environmental index that captures the inherent environmental difference of multi‐environment field trials is crucial to establish relevant findings for a trait of interest to assess critical windows of development.

### 4.2 QTLs linked to GY and its stability

The discovery of QTLs related to GY varied significantly across different environments (Figure 3; Data S1), reflecting the varied genetic segregants impacting GY variation in maize. It should be noted that because segregating lines were evaluated on the same tester, QTLs reflect genetic segregation in the lines that are not masked by the tester. This is more likely to discover practically relevant QTLs as farmers grow hybrids, not inbreds. However, it is also expected that fewer QTLs would be discovered using hybrids instead of inbred lines per se. Only two QTLs of 96 corresponded across individual environments or testers. In terms of stability QTL, there was no correspondence of loci between testers, with the possible exception of the PHK76 and PHZ51 Chr 7 QTL. However, a 6.7 Mb difference in location is unlikely to have the same cause within this higher resolution population. This result of inconsistent loci detected for a complex trait like yield is expected and has been shown in previous genetic mapping studies of both inbred lines and hybrids (Mural et al., 2022; Melchinger et al., 1998). The three tester lines have different loci and alleles from each other, which are expected to mask different segregating loci in the doubled haploid MAGIC lines. Differences in environmental interactions can further cause different segregating loci in the doubled haploid lines and hybrids to have differential effects. The inconsistency of QTL is a major challenge for marker‐assisted breeding of quantitative traits in maize, especially in cumulative traits like GY.

The MAGIC approach to doubled haploid development allowed high resolution of QTL (smaller QTL intervals with fewer candidate genes) as predicted by theory and empirically demonstrated (Anderson et al., 2018). The resulting improved resolution reduced the number of annotated genes in each QTL interval and the likely candidate genes that were investigated. The presence of candidate genes like E3 ubiquitin‐protein ligase RFWD2 and auxin‐responsive proteins in identified QTLs suggests complex regulatory mechanisms influencing yield stability. An auxin biosynthesis pathway was previously identified related to stability performance of plant height and flowering time in maize (Fu & Wang, 2023); similarly, we discovered a segregating auxin‐responsive candidate gene (*Zm00001eb319470*) corresponding to the slope of the GY in hybrid population with PHZ51 tester, grown in the greatest number of environments. Auxin‐responsive genes are related to plant growth and development and are responsive to environmental stimuli (Luo et al., 2018) and were likely to have played a major role on plasticity in G2F maize hybrids used in this study. The *Zm00001eb319470* locus encodes the IAA33–Aux/IAA‐transcription factor 33, known to interact with auxin response factors (ARFs) and inhibit the transcription of genes activated by ARFs. This interaction is essential in the auxin signaling pathway (Luo et al., 2018; Lv et al., 2020), influencing numerous phenotypic traits such as the height from ground to top ear node in maize (Wallace et al., 2014). Variations in *Zm00001eb319470*, as indicated by the environmental index (46–90 days of PTR across 20 environments in Figure 2C), might be associated with the genetic basis for taller plant height and increased yield observed in northern environments compared to southern ones. This hypothesis aligns with the idea that IAA33, encoded by *Zm00001eb319470*, could have enhanced activity in northern environments where longer days relative to GDD could influence its contribution to the stability of GY through plant growth and development processes.

### 4.3 Environmental index‐based genomic prediction for GY

Genomic prediction and selection have been widely implemented for the purpose of selecting the best individuals for overall crop improvement and genetic gain (Crossa et al., 2014; Hunt et al., 2018). Inbred lines and their derived hybrids are easier to analyze for genomic prediction models (and GWAS) than hybrids due to simpler additive models for homozygosity, but inbred lines per se limits the scope of application, and the predictions would not be relevant for more genetically complex hybrids. In addition to additive variation, hybrids are prone to exhibit differential performance across environments resulting from dominance, variable epistatic interactions, and differential gene expression. However, some challenges associated with hybrid genomic prediction may be overcome with sufficient data that account for the phenotypic plasticity, such as multi‐environmental trials on the same set of hybrids. This study demonstrated the ability to predict GY of untested hybrids in diverse maize hybrid populations and environments using a combination of an environmental index and genomic prediction analysis, uniquely through the stability of GY determined by its slope and intercept (Figure 2). The genomic prediction models showed that GY predictions made with untested genotypes in tested environments had reduced prediction accuracy compared to the tested genotypes in untested environments. This could be because the same genotypes performed relatively consistently across all environments or because the hybrids shared similar genetic backgrounds since they were crossed with the same testers, reducing variability in G versus E. However, the proportion of G × E variance (∼12%) was comparable to the amount of genetic variance (∼20%) across this study (Figure 1). Considering the expected genetic variation between the testers in hybrid populations, it is expected that without sufficient performance data on the individual hybrids, the model would be less accurate for untested genotypes. Ideally, a high prediction accuracy value for the untested genotypes in untested environments scenario would provide knowledge for trait stabilities since they are complex and not easily detectable without extensive and costly field trials. This methodology, reflecting the complex interplay between genotype, phenotype, and environment, could allow improved prediction of complex traits with high phenotypic plasticity derived by a combination of environmental clues. Further physiological modeling of yield component traits could provide further gains (Cooper et al., 2016).

The approach of using sliding window‐based environmental indices has been successfully integrated before in predicting flowering times in sorghum and rice (*Oryza sativa* L.) (Li et al., 2018). The novel approach of using a PTR‐based environmental index for genomic prediction was promising based on previous studies in oat (*Avena sativa* L.) and wheat (*Triticum aestivum* L.), where PTR explained the highest amount of variation for mean yield across environments (Guo et al., 2020). Here, PTR was used in maize GY for the first time to our knowledge. The incorporation of a PTR index differs from other genomic prediction studies commonly used to assess the performance of maize lines by also using a biologically relevant sliding window and an extensive set of environments (Choquette et al., 2023; de los Campos et al., 2020; Jarquín et al., 2017). The use of environmental indices generally, and PTR specifically here, provides insights into how specific environmental parameters can be integrated into strategies to enhance yield predictability and stability by determining the timeframe at which an environmental parameter has the most impact on a trait across contrasting environments. As previously mentioned, variability in day length and temperature (GDD) are crucial environmental parameters throughout growth and maturity but are partly confounded; thus, a PTR index can capture both environmental components simultaneously without confounding to assess the performance of a genotype.

### 4.4 Use of these findings

The incorporation of data derived from environmental indices alongside genomic data offers a useful development in methodologies in predictive plant breeding, considering the evolving challenges of climate change and its effects on crop productivity. Utilizing these predictive capabilities can aid researchers in better understanding the diverse trajectories of reaction norms of complex traits. Continued exploration promises to enhance these models further, potentially extending their relevance to a wider variety of crops and future environmental scenarios. Phenomic prediction models, which incorporate temporal phenomic markers of genotypes, may also provide insight on diverse environmental parameters’ effects on plasticity at different stages of a trait (Adak et al., 2023). Rather than focusing on the relationship between temporal environmental parameters affecting a single timepoint related to the terminal stage, temporal phenomic data and crop growth modeling have the potential to capture trait values across different growth periods, which would highlight the stages of a trait that are more correlated with a specific environmental parameter. The combination of G × E data derived from this study along with data from different domains throughout the breeding process may be examined simultaneously using deep learning algorithms to improve existing predictive models that would otherwise present limitations for data‐driven breeding practices (Xu et al., 2022). This could effectively contribute to advancements in crop genetics and breeding practices.

## 5 CONCLUSION

Understanding the underlying role of G × E for complex traits is beneficial for quantifying the phenotypic variation seen within individuals across diverse environments using reaction norms. When combined with genotype data, this framework can uncover loci responsible for trait stabilities. This study sheds light on the varied genetic architecture of GY stability of three hybrid populations exposed to a varying range of PTR values in natural environments. The extent of environments used to derive correlations between PTR and mean GY has not been previously implemented for genomic prediction models in maize. The moderate‐to‐high prediction accuracies calculated for the tested hybrids in tested and untested environments reveal that future breeding efforts can be tailored to accommodate known hybrids based on the patterns of their phenotypic plasticity for GY. This can help future breeding efforts in designing field trials for lines that are either locally adapted to environments or for selecting lines that are stable across multiple environments based on performance predictions from environmental and genetic predictors.

## AUTHOR CONTRIBUTIONS

**Fatma Ozair**: Conceptualization; data curation; formal analysis; investigation; visualization; writing—original draft; writing—review and editing. **Alper Adak**: Conceptualization; data curation; formal analysis; investigation; methodology; project administration; supervision; visualization; writing—original draft; writing—review and editing. **Seth C. Murray**: Conceptualization; data curation; funding acquisition; investigation; methodology; project administration; resources; supervision; writing—original draft; writing—review and editing. **Ryan T. Alpers**: Data curation; project administration; resources. **Alejandro C. Aviles**: Data curation; project administration; resources. **Dayane C. Lima**: Data curation; project administration; resources. **Jode Edwards**: Funding acquisition; resources; validation; writing—review and editing. **David Ertl**: Funding acquisition; project administration; resources; writing—review and editing. **Michael A. Gore**: Data curation; investigation; project administration; resources; writing—review and editing. **Candice N. Hirsch**: Data curation; funding acquisition; investigation; project administration; resources. **Joseph E. Knoll**: Data curation; funding acquisition; project administration; resources. **James C. Schnable**: Data curation; investigation; project administration; resources. **Maninder P. Singh**: Data curation; investigation; project administration; resources; writing—review and editing. **Erin E. Sparks**: Project administration; resources; supervision. **Addie Thompson**: Data curation; funding acquisition; investigation; project administration; resources. **Teclemariam Weldekidan**: Data curation; investigation; project administration; resources. **Wenwei Xu**: Data curation; investigation; project administration; resources.

## CONFLICT OF INTEREST STATEMENT

The authors declare no conflicts of interest.

## Supporting information



Supplemental Table 1 (S1) contains the significant quantitative trait loci (QTLs) discovered using a LOD threshold of 5, along with candidate genes and their functions for grain yield for three different maize tester populations. Supplemental Table 2 (S2) contains the significant quantitative trait loci (QTLs) discovered using a LOD threshold of 5, along with candidate genes and their functions for grain yield slope and intercept for the same three populations as above.

Supplemental Material

## Data Availability

All R codes and input files for this research are stored in GitHub (https://github.com/fatmaoz25/ReactionNormGenomicPrediction) for readers to access.
