# Supplementary material for: Phenotypic plasticity in maize grain yield: Genetic and environmental insights of response to environmental gradients
Source: Plant Genome. 2025 Aug 7;18(3):e70078. doi: 10.1002/tpg2.70078 (PMC12332327; doi:10.1002/tpg2.70078)

Genomic Prediction using Reaction Norm Parameters

Fatma Ozair

2025-04-16

Required Files Below

Data Files Here – Store all files in a folder named **"RNGP"** *(Reaction Norm Genomic Prediction - RNGP)* This will be your **working directory**

Description

One Sentence Summary

The aim of this study is to predict grain yield (tons/hect.) of a set of maize hybrids sharing a common parent by using their genomic and reaction norm data.

General Summary

Grain yield is one of the most important agronomic traits of interest when studying maize. Grain yield is a complex trait that is controlled by many small-effect loci. Therefore, it is difficult to narrow down on the individual genes acting on this trait without reliable modeling scenarios and sufficient data. Aside from genetic variation, grain yield performance of genotypes is also impacted by their environment and genotype-by-environment (GxE) interaction effects. GxE effects arise when a genotype performs differently depending on the environment in which it is in. Thus, it is crucial to account for these additional sources of variation in the model to generate accurate predictions for breeding scenarios where certain genotypes/environments have not been observed/experimented.

GxE can be modeled in terms of the differential mean response across environments (A) or in terms of all the combinations of the genotype:environment (B). The former may be considered to be more computationally efficient than the latter because it requires less terms to be iterated through when performing genomic predictions.

- (A) :

Regression on the mean model (Finlay-Wilkinson): y is the vector for the dependent variable, μ is the grand mean, G_i is the effect of the ith genotype, E_j is the effect of the jth environment, b_iE_j is the reaction norm parameter effect of the ith genotype in the jth environment, and ε is the residual error not explained by the model.

$$y = \mu + G_i + E_j + b_iE_j + \varepsilon$$

- (B) :

Additive effects model: y is the vector for the dependent variable, μ is the grand means, G_i is the effect of the ith genotype, E_j is the effect of the jth envvironment, GE_{ij} is the GxE interaction effect of the ith genotype in the jth environment, and ε is the residual error not explained by the model.

$$y = \mu + G_i + E_j + GE_{ij} + \varepsilon$$

This study used the Genomes to Fields (G2F) 2020 and 2021 data set with the intention of conducting genomic prediction for grain yield (tons/hect.) using reaction norm parameters. Three hybrid tester populations derived from the cross between ex-PVP inbreds (PHK76, PHP02, and PHZ51) and the doubled haploid Wisconsin Stiff Stalk MAGIC population (W10004) were analyzed for a total of 174 hybrids. The study consisted of 29 unique environments across the U.S. to get the full scope of GxE patterns. The photothermal ratio (PTR) was used as a biologically-relevant weather parameter to numerically order the environments to obtain quantified values for grain yield stability for each hybrid. Two different genomic prediction models were conducted (refer to A and B) to compare the results.

Visualize Environments/Trials

Data File(s) Needed:

- envRtype.txt

Code

Load `envRtype.txt` – This data set contains the subset of environments that will be considered for the analysis. The hybrid populations used in the study were planted in these locations. There are **29 total environments (state:year combinations)**.

```
#load libraries
pacman::p_load(readr, ggplot2, maps, ggrepel)

#refer to data set in github repository
git_path <- "https://raw.githubusercontent.com/fatmaoz25/ReactionNormGenomicPrediction/refs/heads/main"
locations_file <- "envRtype.txt"
locations_path <- file.path(git_path, locations_file)

#table summary
locations <- read.table(locations_path, header=T)
head(locations)
```

```
##        env      lat       lon      start     end100     end125 location year
## 1 DEH1.2020 38.63014 -75.46610 2020-05-14 2020-08-22 2020-09-16     DEH1 2020
## 2 DEH1.2021 38.63500 -75.45500 2021-04-27 2021-08-05 2021-08-30     DEH1 2021
## 3 GAH1.2020 31.50728 -83.55828 2020-04-01 2020-07-10 2020-08-04     GAH1 2020
## 4 GAH1.2021 31.50728 -83.55828 2021-03-25 2021-07-03 2021-07-28     GAH1 2021
## 5 IAH1.2021 41.21390 -91.53710 2021-05-01 2021-08-09 2021-09-03     IAH1 2021
## 6 IAH2.2021 42.06830 -94.85980 2021-04-27 2021-08-05 2021-08-30     IAH2 2021
##              Tester
## 1 PHK76,PHP02,PHZ51
## 2 PHK76,PHP02,PHZ51
## 3             PHZ51
## 4             PHZ51
## 5       PHK76,PHZ51
## 6 PHK76,PHP02,PHZ51
```

```r
dim(locations)
```

```
## [1] 29  9
```

```r
#generate U.S. map with state boundaries
us_map <- map_data("state")
```

The figure below displays the environments where each of the hybrid populations were planted and considered for this study. There were 11 environments containing the PHK76 population, 14 environments with PHP02, and 20 environments with PHZ51. Not every tester was featured in every environment because of growth limitations of regionally adapted genotypes. PHK76 is adapted to the Midwest, PHP02 to the North, and PHZ51 to the South.

```
## [1] "Number of Environments with PHK76: 11"

## [1] "Number of Environments with PHP02: 14"

## [1] "Number of Environments with PHZ51: 20"
```

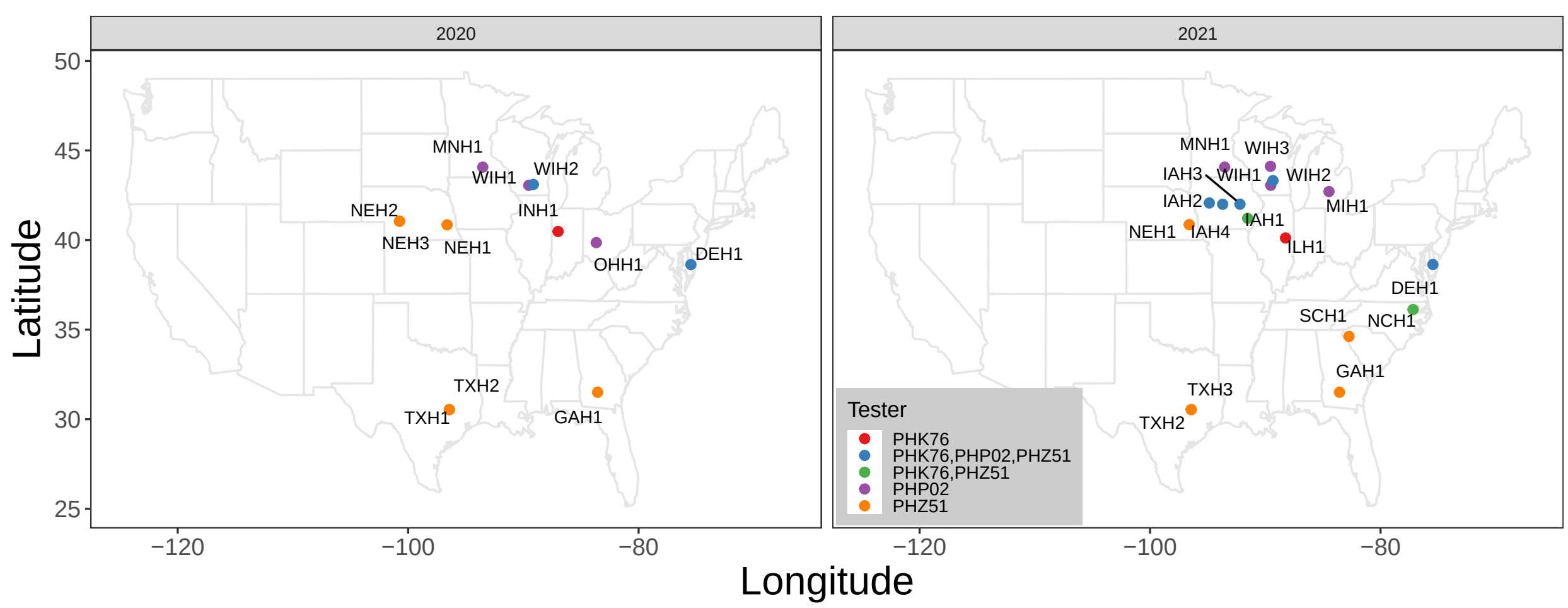

Analysis of Variance (ANOVA)

Data File(s) Needed:

- g2f_2020_phenotypic_clean_data.csv
- g2f_2021_phenotypic_clean_data.csv

Code

Load `g2f_2020_phenotypic_clean_data.csv` and `g2f_2021_phenotypic_clean_data.csv` – The following code combines the 2020 and 2021 G2F data frames for the 29 environments that are of interest for this study.The columns of the data frame are displayed to show the various types of data collected for each observation/hybrid that was planted. For this study, we will be focusing on the column for grain yield (bushels/acre) since this is the trait we want to ultimately analyze.

```r
#load libraries
pacman::p_load(readr, nlme, sjstats, lme4, RColorBrewer, dplyr, ggplot2, tidyverse, ggrepel, caret, MuMI

#refer to data set in github repository
git_path <- "https://raw.githubusercontent.com/fatmaoz25/ReactionNormGenomicPrediction/refs/heads/main"
df20_file <- "g2f_2020_phenotypic_clean_data.csv"
df20_path <- file.path(git_path, df20_file)
df20 <- read.csv(df20_path)


df20 <- df20[df20$Field.Location %in% c("DEH1","GAH1","INH1","MNH1","NEH1","NEH2","NEH3","OHH1","TXH1",

df21_file <- "g2f_2021_phenotypic_clean_data.csv"
df21_path <- file.path(git_path, df21_file)
df21 <- read.csv(df21_path)
df21 <- df21[df21$Field.Location %in% c( "DEH1", "GAH1", "ILH1", "MNH1", "NCH1", "NEH1", "SCH1", "TXH2"

df <- rbind(df20,df21)
df$Env <- paste(df$Field.Location,df$Year, sep = ".")
df <- df[!df$Replicate %in% c(3,4),]
colnames(df)
```

```
##  [1] "Year"
##  [2] "Field.Location"
##  [3] "State"
##  [4] "City"
##  [5] "Plot.length..center.center.in.feet."
##  [6] "Plot.area..ft2."
##  [7] "Alley.length..in.inches."
##  [8] "Row.spacing..in.inches."
##  [9] "Rows.per.plot"
## [10] "X..Seed.per.plot"
## [11] "Experiment"
## [12] "Source"
## [13] "Pedigree"
## [14] "Family"
## [15] "Tester"
## [16] "Replicate"
## [17] "Block"
## [18] "Plot"
## [19] "Plot_ID"
## [20] "Range"
## [21] "Pass"
## [22] "Date.Plot.Planted..MM.DD.YY."
## [23] "Date.Plot.Harvested..MM.DD.YY."
## [24] "Anthesis..MM.DD.YY."
## [25] "Silking..MM.DD.YY."
## [26] "Anthesis..days."
## [27] "Silking..days."
## [28] "Plant.Height..cm."
## [29] "Ear.Height..cm."
## [30] "Stand.Count....of.plants."
## [31] "Root.Lodging....of.plants."
```

```
## [32] "Stalk.Lodging....of.plants."
## [33] "Grain.Moisture...."
## [34] "Test.Weight..lbs."
## [35] "Plot.Weight..lbs."
## [36] "Grain.Yield..bu.A."
## [37] "Plot.Discarded..enter..yes..or.blank."
## [38] "Comments"
## [39] "Filler"
## [40] "Snap....of.plants."
## [41] "Env"
```

Next, we have to run an analysis of variance (ANOVA) to partition the variance components for grain yield in order to obtain heritability estimates. Heritability reveals how well a phenotype is at indicating the breeding value of a genotype that has multiple replicates in multi-environment trials (MET). It is calculated as the ratio between the genotypic variance and the phenotypic variance, thus we can estimate how much of the phenotypic variance is attributed to genetic influences.

This study uses the **Model II ANOVA** in particular, which requires a continuous dependent variable with **random** categorical predictors. This implies that the levels incorporated for the different predictors are sampled from a random population of all possible levels. Treating all factors as random is ideal for this situation since we are not restricting our inferences to ONLY the environments and genotypes observed in this study.

The data used here contains both cross-sectional and longitudinal clustering. The genotypes are assigned to multiple environment, range, and pass levels (cross-sectional clustering) with two replicates each (longitudinal clustering). This clustering violates independence assumptions of a simple linear regression model where there is only a single fixed effect (complete pooling), so we have to use a mixed effects linear model (MLM) to account for hierarchical data structures (partial pooling).

The following code formats the variables of interest as factors to make them compatible for the `lmer()` function. Since each of the observations within these variables is labeled as a random, independent number, we do not want the model to assume that these observations are numerical/ordinal (ordered). Instead we want the model to treat them as nominal (unordered) labels. The default estimation method for `lmer()` is restricted maximum likelihood (REML). REML is an unbiased estimator for variance components because it removes degrees of freedom for every fixed effect estimated for the predictors. Thus, it does not underestimate the variance and works well for smaller sample sizes within the clusters (unlike maximum likelihood (ML)).

The following equation shows the random effects model that was used to dissect the variance components and to calculate the best linear unbiased predictor (BLUP) values for grain yield:

$$Grain\ Yield_{ijklm} = \mu + Genotype_i + Environment_j + GE_{ij} + Range_k + Row_l + Rep_{(j)m} + \varepsilon_{ijklm}$$

μ is the mean grain yield of the population, $Genotype_i$ is the effect of the genotype, $Environment_j$ is the effect of the environment, GE_{ij} is the effect of GxE interactions, $Range_k$ is the effect of the range, Row_l is the effect of the row, $Rep_{(j)m}$ is the effect of the replicate, and ε_{ijklm} is the residual error not explained by the model. **Since all the predictors are treated as random, they are assumed to be normally distributed**

*note: grain yield is converted from bushels/acre to tons/hect. for the model.

```
df$Range <- as.factor(df$Range)
df$Pass <- as.factor(df$Pass)
df$Replicate  <- as.factor(df$Replicate )
df$Pedigree <- as.factor(df$Pedigree)
df$Env <- as.factor(df$Env)
```

```r
df$Grain.Yield..bu.A. <- as.numeric(df$Grain.Yield..bu.A.)

anova<- lmer(Grain.Yield..bu.A.*.0673 ~
               (1|Pedigree)+
               (1|Env)+
               (1|Pedigree:Env)+
               (1|Range)+
               (1|Pass)+
               (1|Replicate:Env), df )
summary(anova)
```

```
## Linear mixed model fit by REML ['lmerMod']
## Formula: Grain.Yield..bu.A. * 0.0673 ~ (1 | Pedigree) + (1 | Env) + (1 |
##     Pedigree:Env) + (1 | Range) + (1 | Pass) + (1 | Replicate:Env)
##    Data: df
##
## REML criterion at convergence: 104045.5
##
## Scaled residuals:
##     Min      1Q  Median      3Q     Max
## -6.5521 -0.4731  0.0266  0.5070  5.3271
##
## Random effects:
##  Groups        Name        Variance Std.Dev.
##  Pedigree:Env  (Intercept) 1.27451  1.1289
##  Pedigree      (Intercept) 1.30754  1.1435
##  Pass          (Intercept) 1.91923  1.3854
##  Replicate:Env (Intercept) 0.16510  0.4063
##  Range         (Intercept) 0.03613  0.1901
##  Env           (Intercept) 3.77574  1.9431
##  Residual                  2.69978  1.6431
## Number of obs: 24171, groups:
## Pedigree:Env, 16680; Pedigree, 1215; Pass, 178; Replicate:Env, 58; Range, 50; Env, 29
##
## Fixed effects:
##             Estimate Std. Error t value
## (Intercept)  10.2982     0.3874   26.58
## optimizer (nloptwrap) convergence code: 0 (OK)
## Model failed to converge with max|grad| = 0.00569172 (tol = 0.002, component 1)
```

Next, we have to compute some metrics to assess the performance of the ANOVA model. The coefficient of variation (cv) shows the relative variability of the datapoints about the mean. The cv is .8%, indicating that the data has tight clustering and low dispersion.

The root mean square error (RMSE) tells us how close the predicted values are to the actual values. In this case, the computed rmse is 1.38, which means the model has good to moderate fit.

The R^2 value of 0.75 indicates the fraction of the variance explained by the fixed and random effects, which is moderately high.

```
## [1] "Coefficient of Variation 0.00888621186362837"
```

```
## [1] "rmse 1.37666717553187"
```

```
## [1] "R 0.758474016749554"
```

To determine the heritability for grain yield, the variance components due to each factor must be extracted to plug-in scaled values for the following equation:

$$Heritability = \frac{\sigma_G}{\sigma_G + \frac{\sigma_{GE}}{n_E} + \frac{\sigma_\varepsilon}{n_E * n_{rep}}}$$

where σ_G is the genotype effects' variance, σ_{GE} is the GxE effects' variance, σ_ε is the residual error variance, n_E is the number of environments, and n_{rep} is the number of reps.

The heritability of 85% is sufficient for a trait as complex as grain yield. This indicates that the expressed phenotypes for each genotype are heritable between the two reps and across different environments.

```
VC<-as.data.frame(print(VarCorr(anova ), comp=c("Variance")))
```

```
##  Groups        Name        Variance
##  Pedigree:Env  (Intercept) 1.274508
##  Pedigree      (Intercept) 1.307537
##  Pass          (Intercept) 1.919233
##  Replicate:Env (Intercept) 0.165097
##  Range         (Intercept) 0.036131
##  Env           (Intercept) 3.775738
##  Residual                  2.699784
```

```
VC$Percent<-round(VC$vcov/sum(VC$vcov)*100,2)

heritability <- VC[2,6] / (VC[2,6] +  VC[1,6]/length(unique(df$Env)) +  (VC[7,6]/length(unique(df$Env))

VC[,7] <-rmse
VC[,8] <-heritability
VC[,9] <-R
names(VC)[7:9] <- c("Rmse","Heritability","R")
head(VC)
```

```
##             grp        var1 var2       vcov     sdcor Percent     Rmse
## 1  Pedigree:Env (Intercept) <NA> 1.27450849 1.1289413   11.40 1.376667
## 2      Pedigree (Intercept) <NA> 1.30753702 1.1434758   11.70 1.376667
## 3          Pass (Intercept) <NA> 1.91923336 1.3853640   17.17 1.376667
## 4 Replicate:Env (Intercept) <NA> 0.16509656 0.4063208    1.48 1.376667
## 5         Range (Intercept) <NA> 0.03613074 0.1900809    0.32 1.376667
## 6           Env (Intercept) <NA> 3.77573824 1.9431259   33.78 1.376667
##   Heritability        R
## 1    0.8503759 0.758474
## 2    0.8503759 0.758474
## 3    0.8503759 0.758474
## 4    0.8503759 0.758474
## 5    0.8503759 0.758474
## 6    0.8503759 0.758474
```

```
head(VC)
```

```
##             grp        var1 var2       vcov     sdcor Percent     Rmse
## 1  Pedigree:Env (Intercept) <NA> 1.27450849 1.1289413   11.40 1.376667
## 2      Pedigree (Intercept) <NA> 1.30753702 1.1434758   11.70 1.376667
## 3          Pass (Intercept) <NA> 1.91923336 1.3853640   17.17 1.376667
## 4 Replicate:Env (Intercept) <NA> 0.16509656 0.4063208    1.48 1.376667
## 5         Range (Intercept) <NA> 0.03613074 0.1900809    0.32 1.376667
## 6           Env (Intercept) <NA> 3.77573824 1.9431259   33.78 1.376667
##   Heritability        R
## 1    0.8503759 0.758474
## 2    0.8503759 0.758474
## 3    0.8503759 0.758474
## 4    0.8503759 0.758474
## 5    0.8503759 0.758474
## 6    0.8503759 0.758474
```

```
dim(VC)
```

```
## [1] 7 9
```

The grain yield BLUPs were calculated by adding the predicted GxE effects for an individual (using `coef()`) with the conditional mode of an individual's environmental and genotype effect (using `ranef()`).

```
E <- ranef(anova)$'Env'
G <- ranef(anova)$'Pedigree'
GE <- coef(anova)$'Pedigree:Env'
head(E)
```

```
##           (Intercept)
## DEH1.2020  -1.2274658
## DEH1.2021   1.1569229
## GAH1.2020  -2.0454634
## GAH1.2021  -0.1697819
## IAH1.2021  -0.7298216
## IAH2.2021   1.1206653
```

```
dim(E)
```

```
## [1] 29  1
```

```
head(G)
```

```
##                   (Intercept)
## 2369/LH123HT       1.42577427
## 6046-BECKS         1.50063518
## ARPA W22 (X17EA) -4.97115243
## B14A/H95         -0.28240151
## B14A/MO17        -0.10269223
## B14A/OH43         0.06600184
```

```r
dim(E)
```

```
## [1] 29  1
```

```r
head(GE)
```

```
##                         (Intercept)
## 2369/LH123HT:DEH1.2020    10.69425
## 2369/LH123HT:DEH1.2021    10.70029
## 2369/LH123HT:GAH1.2020    11.44762
## 2369/LH123HT:GAH1.2021    10.33499
## 2369/LH123HT:IAH1.2021    11.93272
## 2369/LH123HT:IAH2.2021    11.88451
```

```r
dim(GE)
```

```
## [1] 16680     1
```

After calculating the BLUPs, they are stored in a separate data frame where ONLY the genotypes with a Wisconsin stiff stalk parent are kept.

```
##       Yield                interaction          Pedigree      Env Tester
## 1  9.850034 W10004_0006/PHK76:DEH1.2020 W10004_0006/PHK76 DEH1.2020  PHK76
## 2 11.844458 W10004_0006/PHK76:DEH1.2021 W10004_0006/PHK76 DEH1.2021  PHK76
## 3  9.213935 W10004_0006/PHK76:IAH1.2021 W10004_0006/PHK76 IAH1.2021  PHK76
## 4 10.960674 W10004_0006/PHK76:IAH2.2021 W10004_0006/PHK76 IAH2.2021  PHK76
## 5 12.002896 W10004_0006/PHK76:IAH3.2021 W10004_0006/PHK76 IAH3.2021  PHK76
## 6 10.751814 W10004_0006/PHK76:IAH4.2021 W10004_0006/PHK76 IAH4.2021  PHK76
##        Inbred Location Year
## 1 W10004_0006     DEH1 2020
## 2 W10004_0006     DEH1 2021
## 3 W10004_0006     IAH1 2021
## 4 W10004_0006     IAH2 2021
## 5 W10004_0006     IAH3 2021
## 6 W10004_0006     IAH4 2021
```

```
## [1] 15490     8
```

Environmental Index Search

Data File(s) Needed:

- envRtype.txt
- df.clim.csv
- Yield.wide.na.csv
- env.index.phk76.GY.csv
- env.index.php02.GY.csv
- env.index.phz51.GY.csv

Code

The first step in determining the environmental index is to load the `Yield.wide.na.csv` to calculate the mean grain yield value within environments for each tester population.

```
rm(list=ls())
#load packages
pacman::p_load(dplyr, EnvRtype)
git_path <- "https://raw.githubusercontent.com/fatmaoz25/ReactionNormGenomicPrediction/refs/heads/main"

yield_file <- "Yield.wide.na.csv"
yield_path <- file.path(git_path, yield_file)
yield <- read.csv(yield_path)

df.clim_file <- "df.clim.csv"
df.clim_path <- file.path(git_path, df.clim_file)
df.clim <- data.table::fread(df.clim_path)  %>% as.data.frame()

#calculate mean grain yield for each environment
yield <- cbind(yield$Inbred, stack(yield[,3:ncol(yield)]))
yield <- yield %>% tidyr::separate(ind, into = c("Tester", "Env"), sep = "_")
names(yield) <- c("Inbred", "Yield", "Tester", "env")
yield <- yield %>% group_by(env,Tester) %>% mutate(Mean = mean(Yield)) %>% as.data.frame()

#split environmental means according to tester
phk76 <- yield[yield$Tester=="PHK76",]
php02 <- yield[yield$Tester=="PHP02",]
phz51 <- yield[yield$Tester=="PHZ51",]
phk76 <- phk76[!duplicated(phk76$env),][,c(4,5)]
php02 <- php02[!duplicated(php02$env),][,c(4,5)]
phz51 <- phz51[!duplicated(phz51$env),][,c(4,5)]

head(phk76)
```

```
##           env      Mean
## 1   DEH1.2020  9.406983
## 175 DEH1.2021 11.611270
## 349 IAH1.2021  9.866236
## 523 IAH2.2021 11.465615
## 697 IAH3.2021 11.319659
## 871 IAH4.2021 10.809764
```

```
dim(phk76)
```

```
## [1] 11  2
```

```
head(php02)
```

```
##            env      Mean
## 1915 DEH1.2020  9.872847
## 2089 DEH1.2021 12.255644
## 2263 IAH2.2021 12.149298
```

```
## 2437 IAH3.2021 11.808447
## 2611 IAH4.2021 11.323348
## 2785 MIH1.2021 13.092889
```

```
dim(php02)
```

```
## [1] 14  2
```

```
head(phz51)
```

```
##            env      Mean
## 4351 DEH1.2020  8.204401
## 4525 DEH1.2021 10.899782
## 4699 GAH1.2020  7.855889
## 4873 GAH1.2021  9.798067
## 5047 IAH1.2021  9.140951
## 5221 IAH2.2021 11.282331
```

```
dim(phz51)
```

```
## [1] 20  2
```

```
head(df.clim[,c(4,12,24,30,33)])
```

```
##         env   YYYYMMDD        N    GDD      PTR
## 1 DEH1.2020 2020-05-14 14.10979  6.640 2.124969
## 2 DEH1.2020 2020-05-15 14.13917 13.390 1.055950
## 3 DEH1.2020 2020-05-16 14.16795 11.335 1.249930
## 4 DEH1.2020 2020-05-17 14.19613  8.340 1.702174
## 5 DEH1.2020 2020-05-18 14.22370  8.255 1.723041
## 6 DEH1.2020 2020-05-19 14.25063  6.165 2.311538
```

Using the `df.clim` dataset, perform max-min normalization on the day length and growing degree days (GDD) vectors to scale them to be between 0-1. Next, use the normalized values to calculate the photothermal ratio (PTR).

$$PTR = \frac{normalized\ daylength}{normalized\ GDD}$$

The figure below shows PTR plotted from the planting start date to end date. PTR values spiked in numerous environments on certains days between day 0 to day 30. PTR was relatively low in all environments after day 30, but some spikes were seen again toward the end date of day 126. There are not any clear patterns in PTR across the growing season.

```
pacman::p_load(ggplot2, dplyr)

#Assuming your data frame is named 'data' and has columns 'Environment', 'DL', 'GDD', 'Precipitation'
normalize <- function(x) {
  (x - min(x)) / (max(x) - min(x))
}

df.clim <- df.clim %>%
```

```r
  group_by(env) %>%
  mutate(
    DL_normalized = normalize(N),
    GDD_normalized = normalize(GDD),
    Precipitation_normalized = normalize(PRECTOT)
  ) %>%
  ungroup() %>% as.data.frame()

head(df.clim[,c(4,12,35,36)])
```

```
##          env   YYYYMMDD DL_normalized GDD_normalized
## 1 DEH1.2020 2020-05-14     0.7688341      0.1050640
## 2 DEH1.2020 2020-05-15     0.7802959      0.4979627
## 3 DEH1.2020 2020-05-16     0.7915285      0.3783469
## 4 DEH1.2020 2020-05-17     0.8025259      0.2040163
## 5 DEH1.2020 2020-05-18     0.8132826      0.1990687
## 6 DEH1.2020 2020-05-19     0.8237927      0.0774156
```

```r
df.clim$PTR_normalized <-df.clim$DL_normalized/df.clim$GDD_normalized


p<-ggplot(df.clim, aes(x=daysFromStart, y=PTR_normalized, group=env)) +
  geom_line(aes(color=env))
#  geom_point(aes(color=supp))
p
```

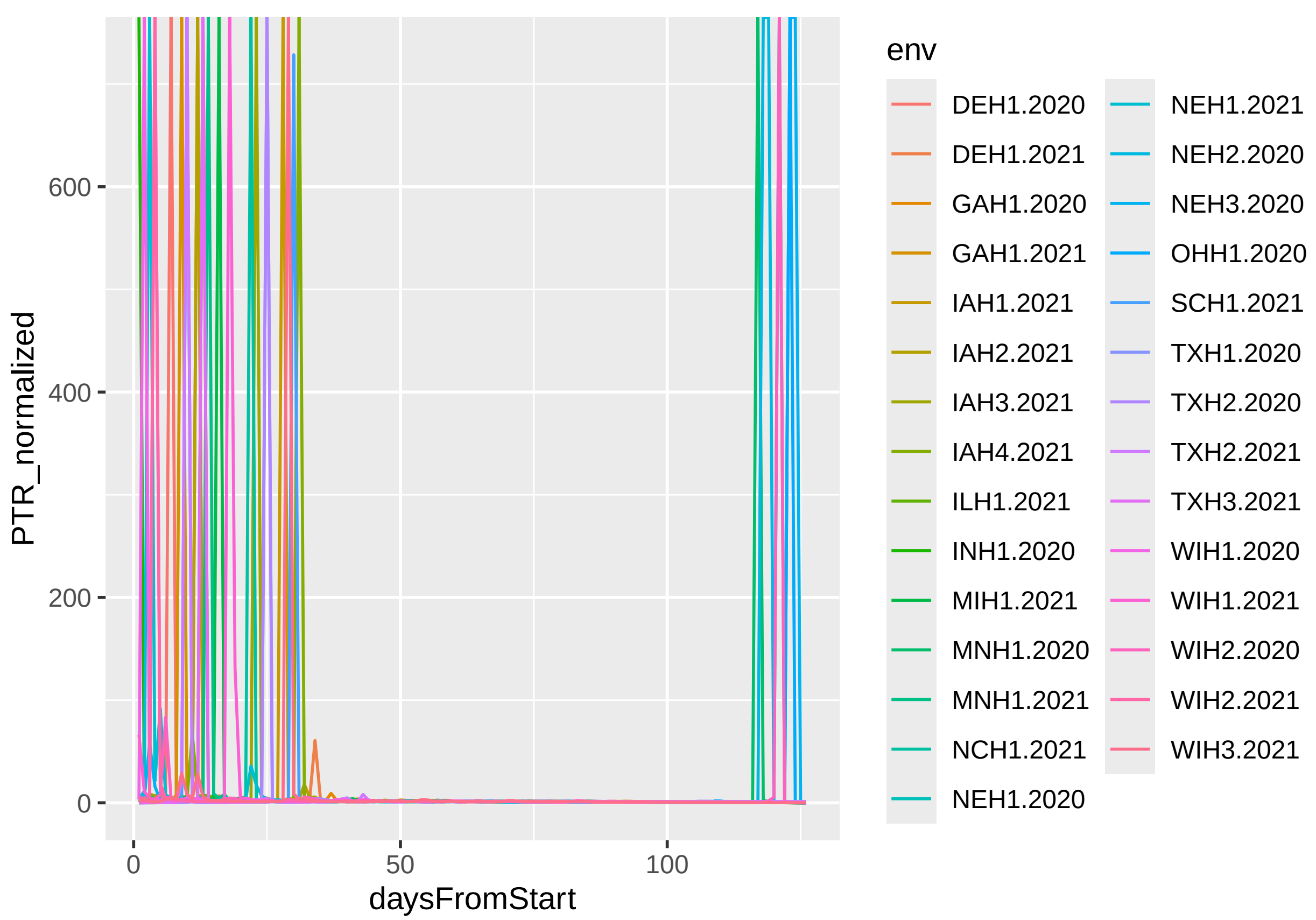

The chunk of code below first creates a data frame that calculates the mean PTR value for each window size for each of the days in each environment (day 1 - day 126). Next, it calculates the mean PTR value in each environment for all time window sizes from day 1 - day 126.

```
pacman::p_load(data.table, zoo)

#PTR wide format
temperature_data <- reshape2::dcast(df.clim,  daysFromStart~env, value.var = "PTR")
head(temperature_data[1:5])
```

```
##   daysFromStart DEH1.2020 DEH1.2021 GAH1.2020 GAH1.2021
## 1             1  2.124969 1.5063982  2.466519 0.9370904
## 2             2  1.055950 0.9818530  1.993915 0.8042488
## 3             3  1.249930 0.9002999  1.408226 0.8178058
## 4             4  1.702174 2.0682142  1.109167 0.9425414
## 5             5  1.723041 2.6677056  1.024117 1.6651354
## 6             6  2.311538 1.3492508  1.049985 1.0610927
```

```
names(temperature_data)[1] <- "day"
dim(temperature_data)
```

```
## [1] 126  30
```

```
set.seed(123) #Ensure reproducibility

#Define a function to calculate mean temperatures for all window sizes for a given environment
calculate_mean_temps_for_all_windows <- function(temperature_vector) {
  results <- list()
  total_days <- length(temperature_vector)

  for (window_size in 1:total_days) {
    mean_temps <- sapply(1:(total_days-window_size+1), function(start_day) {
      mean(temperature_vector[start_day:(start_day+window_size-1)])
    })
    results[[as.character(window_size)]] <- mean_temps
  }

  return(results)
}

#Apply the function to each environment column (excluding the 'day' column)
all_env_means <- list()

for (env in names(temperature_data)[-1]) { # Skip 'day' column
  env_data <- temperature_data[[env]]
  all_env_means[[env]] <- calculate_mean_temps_for_all_windows(env_data)
}


# Initialize an empty data.table to store the results
results_dt <- data.table(environment = character(),
                         window_size = integer(),
```

```r
                         start_day = integer(),
                         end_day = integer(),
                         mean_temperature = numeric())

# Environment names, assuming they were the column names in your original temperature_data
environment_names <- names(temperature_data)[-1]

# Flatten the results and populate results_dt
for (i in seq_along(all_env_means)) {
  env_results <- all_env_means[[i]] # Results for the current environment
  env_name <- environment_names[i]

  for (window_size in seq_along(env_results)) {
    window_data <- env_results[[window_size]]
    if (is.null(window_data)) next # Skip if there's no data for this window size

    # Calculate start and end days for each window
    start_days <- seq_len(length(window_data))
    end_days <- start_days + window_size - 1

    # Append to results_dt
    results_dt <- rbindlist(list(results_dt, data.table(environment = env_name,
                                                        window_size = window_size,
                                                        start_day = start_days,
                                                        end_day = end_days,
                                                        mean_temperature = window_data)),
                            use.names = TRUE, fill = TRUE)
  }
}

results_dt <- results_dt %>% as.data.frame()

head(results_dt)
```

```
##   environment window_size start_day end_day mean_temperature
## 1   DEH1.2020           1         1       1         2.124969
## 2   DEH1.2020           1         2       2         1.055950
## 3   DEH1.2020           1         3       3         1.249930
## 4   DEH1.2020           1         4       4         1.702174
## 5   DEH1.2020           1         5       5         1.723041
## 6   DEH1.2020           1         6       6         2.311538
```

```r
names(results_dt)[1] <- "env"
dim(results_dt)
```

```
## [1] 232029      5
```

The following chunks of code go through the sliding window analysis for each tester population separately. A data frame is created displaying the mean PTR values (`mean_temperature` vector) for window sizes from 1 day - 126 days of all the possible day combinations. The mean PTR values for each window size and day combination (w:d) were calculated for each environment for each tester population. Next, the correlation (Pearson) between the mean PTR for each w:d and the mean grain yield across all the environments. Thus,

the x-axis contained the PTR values for the specified window sizes and the y-axis contained the grain yield BLUP mean values for the environments. The time window corresponding to the highest correlation between the mean PTR values and grain yield BLUP means across all environments was noted, and the x-axis values were retained as the environment index values.

- **PHK76 Analysis**

```
pacman::p_load(ggplot2,ggrepel,ggpubr)

phk76.cor <- dplyr::left_join(results_dt, phk76, by="env")
phk76.cor <- na.omit(phk76.cor)

phk76.cor <- phk76.cor %>% group_by(start_day,end_day) %>% mutate(cor=cor(mean_temperature,Mean,use = "
head(phk76.cor)
```

```
##          env window_size start_day end_day mean_temperature     Mean         cor
## 1 DEH1.2020           1         1       1         2.124969 9.406983  0.17888758
## 2 DEH1.2020           1         2       2         1.055950 9.406983 -0.29727344
## 3 DEH1.2020           1         3       3         1.249930 9.406983 -0.11207047
## 4 DEH1.2020           1         4       4         1.702174 9.406983  0.30037507
## 5 DEH1.2020           1         5       5         1.723041 9.406983  0.33590557
## 6 DEH1.2020           1         6       6         2.311538 9.406983 -0.02302635
```

```
max_cor <- max(phk76.cor$cor)
max_cor
```

```
## [1] 0.9582252
```

```
git_path <- "https://raw.githubusercontent.com/fatmaoz25/ReactionNormGenomicPrediction/refs/heads/main"

phk_file <- "env.index.phk76.GY.csv"
phk_path <- file.path(git_path, phk_file)
dff <- read.csv(phk_path)

p <- ggplot(phk76.cor,aes(x= start_day ,y=end_day))+
  geom_tile(aes(fill=cor))+
  annotate("segment",x=0,xend=unique(dff$start_day), y=unique(dff$end_day), yend=unique(dff$end_day),co
  annotate("segment",x=unique(dff$start_day),xend=unique(dff$start_day), y=0, yend=unique(dff$end_day),
  #annotate("segment", x = 0, xend = unique(dff$start_day), y = dff$end_day, yend = unique(dff$end_day)
  geom_point(dff, mapping=aes(start_day, end_day),shape=1, size=4, color="black")+
  xlab("Begining of window (days)")+
  annotate("text",
           label = paste( "(", unique(dff$start_day),",",unique(dff$end_day)  ,")",   sep=""),
           x = dff$start_day+17, y = dff$end_day, colour = "black"
  )+
  #  geom_label(label = paste( "(", unique(dff$start_day),",",unique(dff$end_day)  ,")",   sep=""))+
  ylab("End of window (days)")+
  theme_bw()+
  scale_fill_distiller("cor (R)",palette = "Spectral",breaks = seq(from = -1, to = 1, by = 0.2))+
  #  scale_fill_continuous(colors = "viridis")+
  #  scale_fill_viridis_c()+
  # scale_fill_gradient2(low = "darkgreen", high = "brown",mid="white",midpoint=0,breaks = seq(from = -
```

```
  theme(panel.background = element_blank(),
        axis.line = element_line(colour="black"))+
  #ggtitle(name_cor_plot[q])+
  theme(plot.title = element_text(hjust = 0.5),
        #  text = element_text(family = "sans"),
        legend.position = c(0.9,0.3),
        panel.grid.major = element_blank(),
        panel.grid.minor = element_blank(),
        legend.key.size = unit(12,"pt"))+
  scale_x_continuous(breaks = c(0,10,20,30,40,50,60,70,80,90,100,110,120))+
  scale_y_continuous(breaks = c(0,10,20,30,40,50,60,70,80,90,100,110,120))
p
```

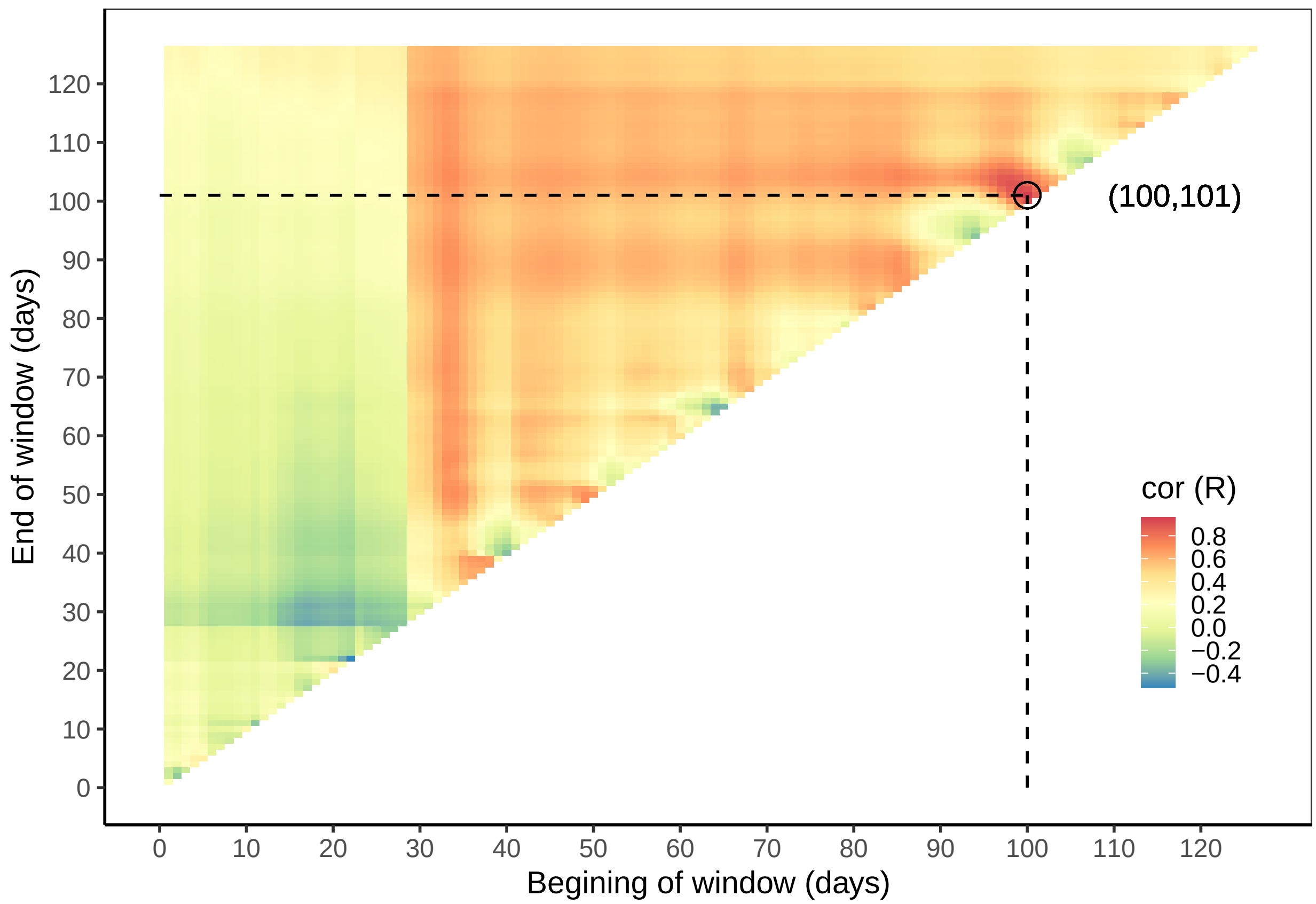


```
p1 <- ggplot(data= dff, aes(x=mean_temperature, y=Mean, group=1)) +
  geom_smooth(method = lm, se = T)+
  geom_point(color="red", size=3)+
  geom_text_repel(aes(label = env),size=3 )+
  theme_bw()+
  theme(panel.grid.major = element_blank(),
        panel.grid.minor = element_blank())+
  stat_cor(method = "pearson")+
  scale_y_continuous("Environmental mean of yield (t/ha)")+
  scale_x_continuous( paste("PTR"," ", "(", unique(dff$start_day),",",unique(dff$end_day)  ,")",    sep=
p1
```

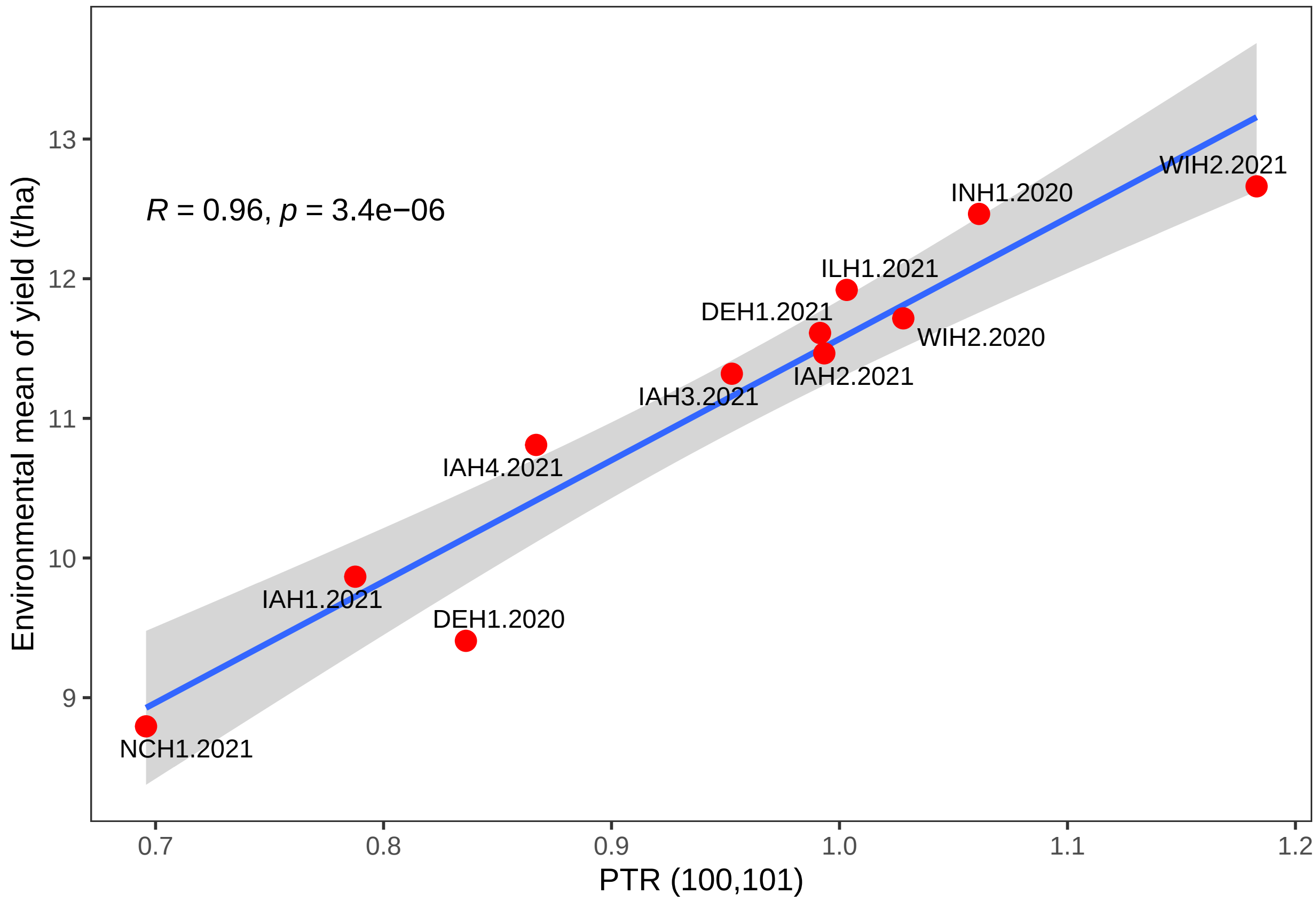

- **PHP02 Analysis**

```
##          env window_size start_day end_day mean_temperature     Mean         cor
## 1 DEH1.2020           1         1       1         2.124969 9.872847  0.15639938
## 2 DEH1.2020           1         2       2         1.055950 9.872847         NaN
## 3 DEH1.2020           1         3       3         1.249930 9.872847 -0.03450445
## 4 DEH1.2020           1         4       4         1.702174 9.872847         NaN
## 5 DEH1.2020           1         5       5         1.723041 9.872847  0.11963817
## 6 DEH1.2020           1         6       6         2.311538 9.872847 -0.06770063
```

```
## [1] 0.6926673
```

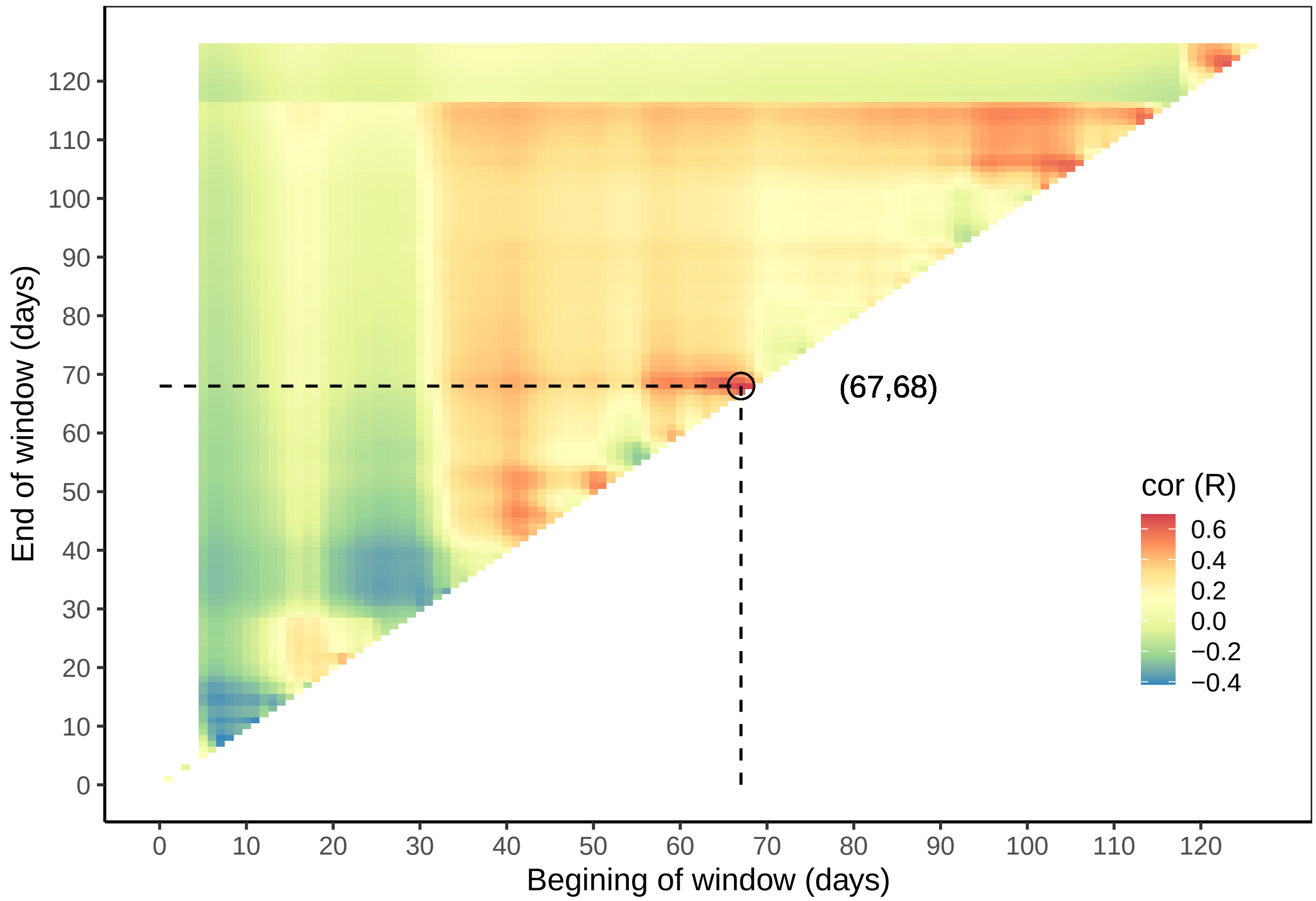

(67,68)
cor (R)
0.6
0.4
0.2
0.0
−0.2
−0.4
End of window (days)
Begining of window (days)
0
10
20
30
40
50
60
70
80
90
100
110
120

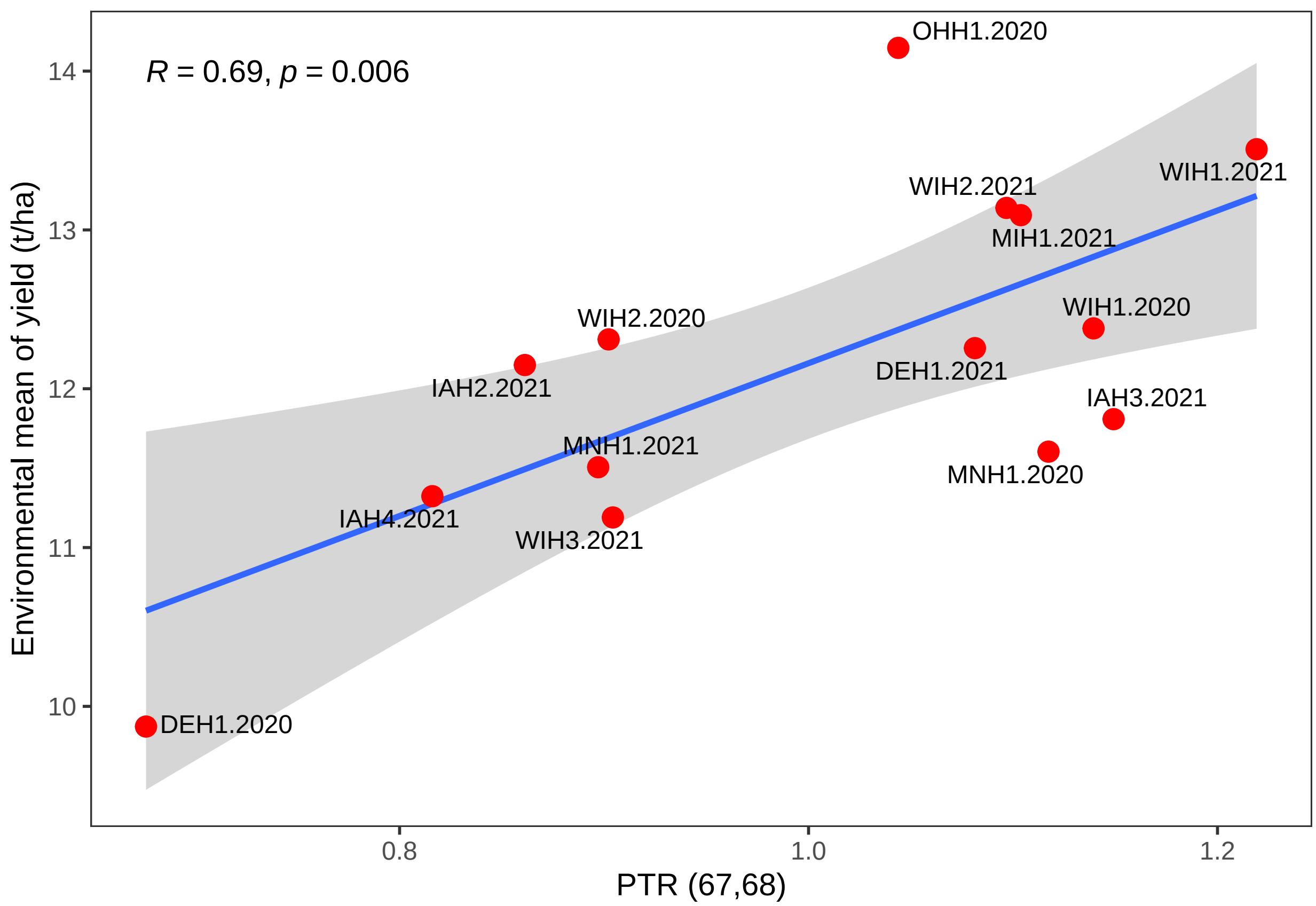

- **PHZ51 Analysis**

```
##          env window_size start_day end_day mean_temperature     Mean       cor
## 1 DEH1.2020           1         1       1         2.124969 8.204401 0.3028280
## 2 DEH1.2020           1         2       2         1.055950 8.204401 0.1893633
## 3 DEH1.2020           1         3       3         1.249930 8.204401 0.2216891
## 4 DEH1.2020           1         4       4         1.702174 8.204401 0.4232868
## 5 DEH1.2020           1         5       5         1.723041 8.204401 0.5771262
## 6 DEH1.2020           1         6       6         2.311538 8.204401 0.4597236
```

```
## [1] 0.7326232
```

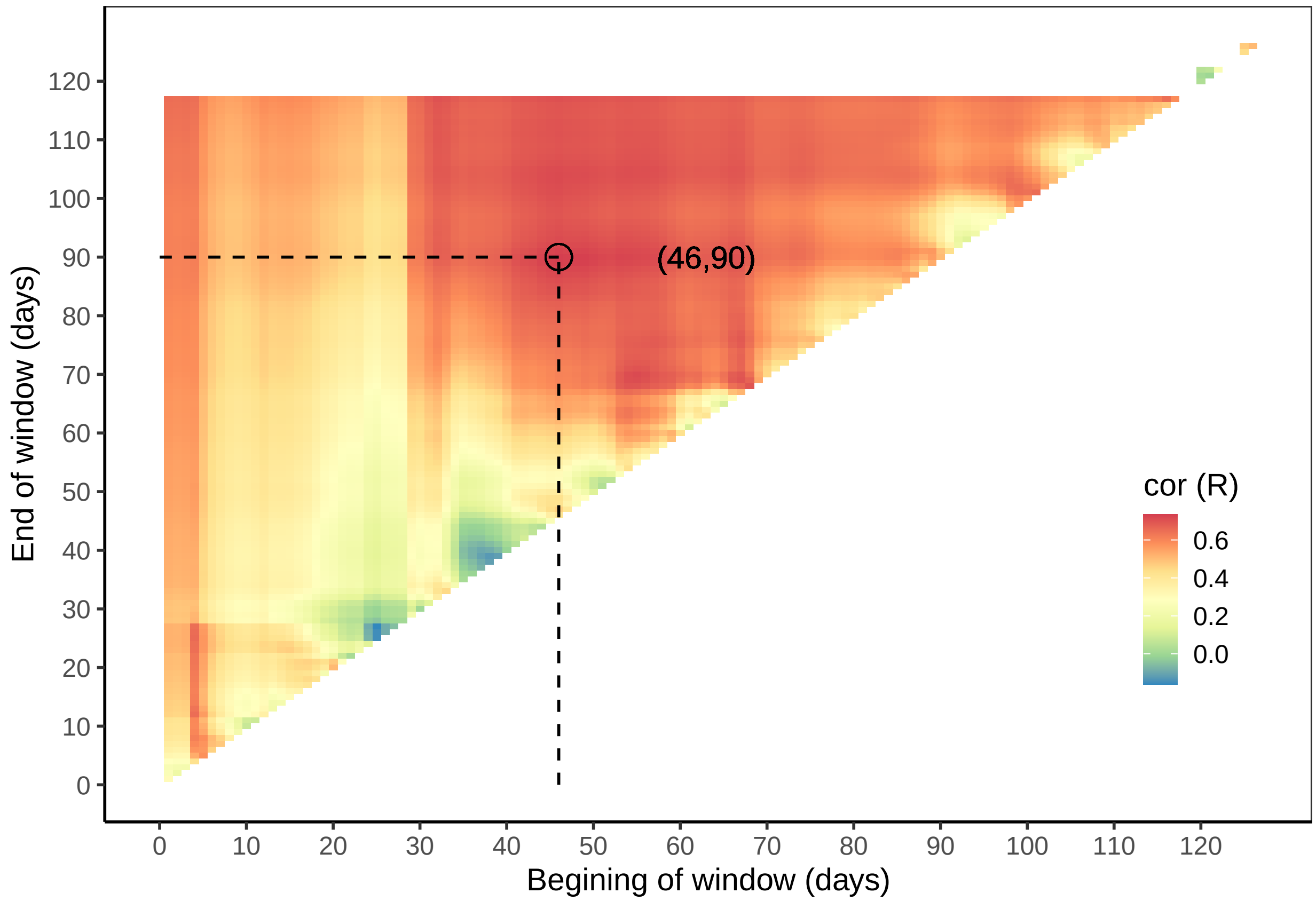

End of window (days)
Begining of window (days)
(46,90)
cor (R)
0.6
0.4
0.2
0.0

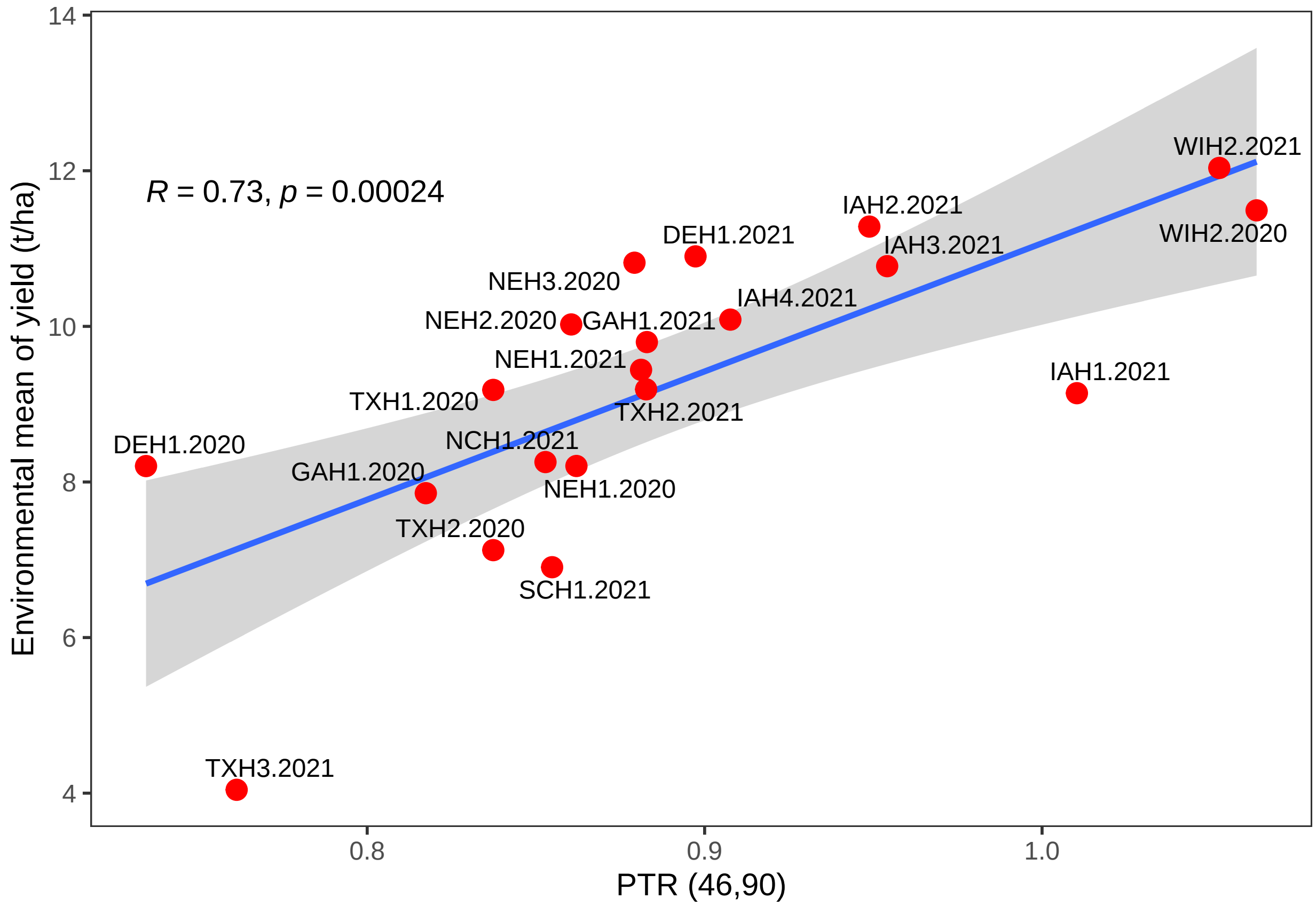

Reaction Norm Modeling

Data Files Needed:

- Yield.wide.na.csv
- env.index.phk76.GY.csv
- env.index.php02.GY.csv
- env.index.phz51.GY.csv

Code:

The following code goes through each tester population to plot the grain yield plasticity parameters for each hybrid – the fit slopes and intercepts when plotting the hybrid grain yield BLUPs against the PTR values identified from the environmental index search.

- **PHK76 Analysis**

```
pacman::p_load(ggplot2,ggrepel,ggpubr,dplyr,data.table)

git_path <- "https://raw.githubusercontent.com/fatmaoz25/ReactionNormGenomicPrediction/refs/heads/main"

yield_file <- "Yield.wide.na.csv"
yield_path <- file.path(git_path, yield_file)
```

```r
GY.wide <- read.csv(yield_path)

GY.phk76 <- GY.wide[,c(2,3,4,5,6,7,8,9,10,11,12,13)]
GY.phk76 <- cbind(GY.phk76[,1], stack(GY.phk76[,2:ncol(GY.phk76)]))
names(GY.phk76) <- c("Inbred", "GY", "env")

GY.phk76 <- GY.phk76 %>% tidyr::separate(env, into = c("Tester", "env"), sep = "_")
head(GY.phk76)
```

```
##        Inbred        GY Tester      env
## 1 W10004_0007 10.395594  PHK76 DEH1.2020
## 2 W10004_0011  8.371664  PHK76 DEH1.2020
## 3 W10004_0014 11.050319  PHK76 DEH1.2020
## 4 W10004_0018  9.368259  PHK76 DEH1.2020
## 5 W10004_0026 10.437017  PHK76 DEH1.2020
## 6 W10004_0032  9.778872  PHK76 DEH1.2020
```

```r
dim(GY.phk76)
```

```
## [1] 1914    4
```

```r
phk_file <- "env.index.phk76.GY.csv"
phk_path <- file.path(git_path, phk_file)
env.index.phk76.GY <- read.csv(phk_path)
head(env.index.phk76.GY)
```

```
##       X       env window_size start_day end_day mean_temperature      Mean
## 1   226 DEH1.2020           2       100     101        0.8361215  9.406983
## 2  8227 DEH1.2021           2       100     101        0.9914683 11.611270
## 3 16228 IAH1.2021           2       100     101        0.7875916  9.866236
## 4 24229 IAH2.2021           2       100     101        0.9933251 11.465615
## 5 32230 IAH3.2021           2       100     101        0.9527715 11.319659
## 6 40231 IAH4.2021           2       100     101        0.8669274 10.809764
##         cor
## 1 0.9582252
## 2 0.9582252
## 3 0.9582252
## 4 0.9582252
## 5 0.9582252
## 6 0.9582252
```

```r
dim(env.index.phk76.GY)
```

```
## [1] 11  8
```

```r
GY.phk76.stability <- dplyr::left_join(GY.phk76, env.index.phk76.GY, by="env")
head(GY.phk76.stability)
```

```
##        Inbred        GY Tester      env   X window_size start_day end_day
## 1 W10004_0007 10.395594  PHK76 DEH1.2020 226           2       100     101
```

```
## 2 W10004_0011  8.371664  PHK76 DEH1.2020 226           2       100      101
## 3 W10004_0014 11.050319  PHK76 DEH1.2020 226           2       100      101
## 4 W10004_0018  9.368259  PHK76 DEH1.2020 226           2       100      101
## 5 W10004_0026 10.437017  PHK76 DEH1.2020 226           2       100      101
## 6 W10004_0032  9.778872  PHK76 DEH1.2020 226           2       100      101
##   mean_temperature     Mean       cor
## 1        0.8361215 9.406983 0.9582252
## 2        0.8361215 9.406983 0.9582252
## 3        0.8361215 9.406983 0.9582252
## 4        0.8361215 9.406983 0.9582252
## 5        0.8361215 9.406983 0.9582252
## 6        0.8361215 9.406983 0.9582252
```

```
dim(GY.phk76.stability)
```

```
## [1] 1914   11
```

- **PHP02 Analysis**

```
git_path <- "https://raw.githubusercontent.com/fatmaoz25/ReactionNormGenomicPrediction/refs/heads/main"

yield_file <- "Yield.wide.na.csv"
yield_path <- file.path(git_path, yield_file)
GY.wide <- read.csv(yield_path)

GY.php02 <- GY.wide[,c(2,14:27)]
head(GY.php02)
```

```
##        Inbred PHP02_DEH1.2020 PHP02_DEH1.2021 PHP02_IAH2.2021 PHP02_IAH3.2021
## 1 W10004_0007        9.797786        12.84561        11.73630       11.843965
## 2 W10004_0011        9.815967        11.56275        11.23017       11.297544
## 3 W10004_0014       11.129001        14.00365        14.94558       14.603535
## 4 W10004_0018        8.519591        10.47899        10.12657        9.690322
## 5 W10004_0026       10.933065        12.81375        13.40871       12.058114
## 6 W10004_0032        9.693562        12.10590        12.20176       12.389796
##   PHP02_IAH4.2021 PHP02_MIH1.2021 PHP02_MNH1.2020 PHP02_MNH1.2021
## 1        11.59665       14.424982       12.551783        11.60085
## 2        10.37560       12.093223       10.532706        10.96900
## 3        13.52296       15.548537       14.191185        13.62540
## 4        10.06994        9.963197        8.576707        10.02438
## 5        12.39005       13.851457       12.152802        12.09161
## 6        11.26940       13.467011       11.654503        11.44477
##   PHP02_OHH1.2020 PHP02_WIH1.2020 PHP02_WIH1.2021 PHP02_WIH2.2020
## 1        14.45873        12.82516        14.20236        13.888174
## 2        13.58197        11.69032        13.30720         9.696058
## 3        16.49231        14.85796        15.97108        12.645385
## 4        12.64049        10.55475        12.36739        10.381469
## 5        15.08682        13.62246        13.84801        13.674190
## 6        14.18127        11.78967        12.78811        10.761356
##   PHP02_WIH2.2021 PHP02_WIH3.2021
## 1        13.63586       11.557067
## 2        11.87131       10.402453
```

```
## 3        14.88880       14.251496
## 4        11.57282        8.698027
## 5        13.77506       12.408980
## 6        11.82213       10.774981
```

```
GY.php02 <- cbind(GY.php02[,1], stack(GY.php02[,2:ncol(GY.php02)]))
names(GY.php02) <- c("Inbred", "GY", "env")
GY.php02 <- GY.php02 %>% tidyr::separate(env, into = c("Tester", "env"), sep = "_")

head(GY.php02)
```

```
##        Inbred        GY Tester       env
## 1 W10004_0007  9.797786  PHP02 DEH1.2020
## 2 W10004_0011  9.815967  PHP02 DEH1.2020
## 3 W10004_0014 11.129001  PHP02 DEH1.2020
## 4 W10004_0018  8.519591  PHP02 DEH1.2020
## 5 W10004_0026 10.933065  PHP02 DEH1.2020
## 6 W10004_0032  9.693562  PHP02 DEH1.2020
```

```
php_file <- "env.index.php02.GY.csv"
php_path <- file.path(git_path, php_file)
env.index.php02.GY <- read.csv(php_path)
head(env.index.php02.GY)
```

```
##       X       env window_size start_day end_day mean_temperature      Mean
## 1   193 DEH1.2020           2        67      68        0.6759941  9.872847
## 2  8194 DEH1.2021           2        67      68        1.0813678 12.255644
## 3 40198 IAH2.2021           2        67      68        0.8612614 12.149298
## 4 48199 IAH3.2021           2        67      68        1.1491964 11.808447
## 5 56200 IAH4.2021           2        67      68        0.8160279 11.323348
## 6 80203 MIH1.2021           2        67      68        1.1038191 13.092889
##         cor
## 1 0.6926673
## 2 0.6926673
## 3 0.6926673
## 4 0.6926673
## 5 0.6926673
## 6 0.6926673
```

```
GY.php02.stability <- dplyr::left_join(GY.php02, env.index.php02.GY, by="env")
head(GY.php02.stability)
```

```
##        Inbred        GY Tester       env   X window_size start_day end_day
## 1 W10004_0007  9.797786  PHP02 DEH1.2020 193           2        67      68
## 2 W10004_0011  9.815967  PHP02 DEH1.2020 193           2        67      68
## 3 W10004_0014 11.129001  PHP02 DEH1.2020 193           2        67      68
## 4 W10004_0018  8.519591  PHP02 DEH1.2020 193           2        67      68
## 5 W10004_0026 10.933065  PHP02 DEH1.2020 193           2        67      68
## 6 W10004_0032  9.693562  PHP02 DEH1.2020 193           2        67      68
##   mean_temperature     Mean       cor
## 1        0.6759941 9.872847 0.6926673
## 2        0.6759941 9.872847 0.6926673
```

```
## 3        0.6759941 9.872847 0.6926673
## 4        0.6759941 9.872847 0.6926673
## 5        0.6759941 9.872847 0.6926673
## 6        0.6759941 9.872847 0.6926673
```

- **PHZ51 Analysis**

```
git_path <- "https://raw.githubusercontent.com/fatmaoz25/ReactionNormGenomicPrediction/refs/heads/main"

yield_file <- "Yield.wide.na.csv"
yield_path <- file.path(git_path, yield_file)
GY.wide <- read.csv(yield_path)

GY.phz51 <- GY.wide[,c(2,28:47)]
head(GY.phz51)
```

```
##        Inbred PHZ51_DEH1.2020 PHZ51_DEH1.2021 PHZ51_GAH1.2020 PHZ51_GAH1.2021
## 1 W10004_0007        9.288831        11.21449        8.949733       10.305163
## 2 W10004_0011        7.091116        10.25501        6.591163        8.899289
## 3 W10004_0014        9.398324        11.84821        8.225487        9.566614
## 4 W10004_0018        6.668914        10.32386        6.935572        8.801453
## 5 W10004_0026        7.800206        11.08133        8.321225       10.032927
## 6 W10004_0032        7.589699        11.23896        7.073529        9.342681
##   PHZ51_IAH1.2021 PHZ51_IAH2.2021 PHZ51_IAH3.2021 PHZ51_IAH4.2021
## 1        8.101275        11.82649       12.210923       10.163747
## 2        7.812028        10.34810        8.924262        9.195727
## 3       10.358976        12.02660       11.069983       11.259271
## 4        8.906650        10.80153       10.629378        9.640500
## 5        8.950674        10.78451        9.093160        9.524618
## 6        9.313303        11.44250       10.764499       10.134623
##   PHZ51_NCH1.2021 PHZ51_NEH1.2020 PHZ51_NEH1.2021 PHZ51_NEH2.2020
## 1        8.574247        9.314713       11.192281       10.652657
## 2        7.183664        7.953082        7.573318        7.175881
## 3        8.550132        9.632448       11.335435       10.293771
## 4        7.597329        7.205523        6.610941        9.824061
## 5        8.611617        7.208446        9.142053       12.676529
## 6        8.669857        8.602385       10.711584       10.680057
##   PHZ51_NEH3.2020 PHZ51_SCH1.2021 PHZ51_TXH1.2020 PHZ51_TXH2.2020
## 1        10.59540        7.200764        9.207735        9.392135
## 2        10.14526        5.761004        7.924498        5.921937
## 3        11.48039        8.362518       10.577292        7.474514
## 4        10.13814        6.655994        8.097084        5.973930
## 5        10.18102        7.049889        9.664098        7.461703
## 6        10.26019        7.836149       10.140324        8.036569
##   PHZ51_TXH2.2021 PHZ51_TXH3.2021 PHZ51_WIH2.2020 PHZ51_WIH2.2021
## 1        8.875863        4.202074        9.474609        11.80527
## 2        8.953560        3.310938        9.301504        10.97433
## 3        8.763002        4.377614       12.311423        13.74152
## 4        7.387736        3.414941       11.224346        11.00785
## 5       10.116463        3.720163       10.653223        11.03379
## 6        9.775729        3.715120       12.204420        11.63943
```

```r
GY.phz51 <- cbind(GY.phz51[,1], stack(GY.phz51[,2:ncol(GY.phz51)]))
names(GY.phz51) <- c("Inbred", "GY", "env")
GY.phz51 <- GY.phz51 %>% tidyr::separate(env, into = c("Tester", "env"), sep = "_")

head(GY.phz51)
```

```
##        Inbred       GY Tester      env
## 1 W10004_0007 9.288831  PHZ51 DEH1.2020
## 2 W10004_0011 7.091116  PHZ51 DEH1.2020
## 3 W10004_0014 9.398324  PHZ51 DEH1.2020
## 4 W10004_0018 6.668914  PHZ51 DEH1.2020
## 5 W10004_0026 7.800206  PHZ51 DEH1.2020
## 6 W10004_0032 7.589699  PHZ51 DEH1.2020
```

```r
phz_file <- "env.index.phz51.GY.csv"
phz_path <- file.path(git_path, phz_file)
env.index.phz51.GY <- read.csv(phz_path)
head(env.index.phz51.GY)
```

```
##       X       env window_size start_day end_day mean_temperature      Mean
## 1  4644 DEH1.2020          45        46      90        0.7344577  8.204401
## 2 12645 DEH1.2021          45        46      90        0.8972993 10.899782
## 3 20646 GAH1.2020          45        46      90        0.8173709  7.855889
## 4 28647 GAH1.2021          45        46      90        0.8828835  9.798067
## 5 36648 IAH1.2021          45        46      90        1.0103203  9.140951
## 6 44649 IAH2.2021          45        46      90        0.9488085 11.282331
##         cor
## 1 0.7326232
## 2 0.7326232
## 3 0.7326232
## 4 0.7326232
## 5 0.7326232
## 6 0.7326232
```

```r
GY.phz51.stability <- dplyr::left_join(GY.phz51, env.index.phz51.GY, by="env")
head(GY.phz51.stability)
```

```
##        Inbred       GY Tester       env    X window_size start_day end_day
## 1 W10004_0007 9.288831  PHZ51 DEH1.2020 4644          45        46      90
## 2 W10004_0011 7.091116  PHZ51 DEH1.2020 4644          45        46      90
## 3 W10004_0014 9.398324  PHZ51 DEH1.2020 4644          45        46      90
## 4 W10004_0018 6.668914  PHZ51 DEH1.2020 4644          45        46      90
## 5 W10004_0026 7.800206  PHZ51 DEH1.2020 4644          45        46      90
## 6 W10004_0032 7.589699  PHZ51 DEH1.2020 4644          45        46      90
##   mean_temperature     Mean       cor
## 1        0.7344577 8.204401 0.7326232
## 2        0.7344577 8.204401 0.7326232
## 3        0.7344577 8.204401 0.7326232
## 4        0.7344577 8.204401 0.7326232
## 5        0.7344577 8.204401 0.7326232
## 6        0.7344577 8.204401 0.7326232
```

The data sets for the individual tester populations from the above code are combined below to create a combined data set to display the reaction norms of each hybrid across the numerically-ordered environments according to increasing mean PTR.

- **Reaction Norm Plotting**

```
GY.stability <- rbind(GY.phk76.stability,GY.php02.stability,GY.phz51.stability)
head(GY.stability)
```

```
##         Inbred        GY Tester       env   X window_size start_day end_day
## 1 W10004_0007 10.395594  PHK76 DEH1.2020 226           2       100     101
## 2 W10004_0011  8.371664  PHK76 DEH1.2020 226           2       100     101
## 3 W10004_0014 11.050319  PHK76 DEH1.2020 226           2       100     101
## 4 W10004_0018  9.368259  PHK76 DEH1.2020 226           2       100     101
## 5 W10004_0026 10.437017  PHK76 DEH1.2020 226           2       100     101
## 6 W10004_0032  9.778872  PHK76 DEH1.2020 226           2       100     101
##   mean_temperature     Mean       cor
## 1        0.8361215 9.406983 0.9582252
## 2        0.8361215 9.406983 0.9582252
## 3        0.8361215 9.406983 0.9582252
## 4        0.8361215 9.406983 0.9582252
## 5        0.8361215 9.406983 0.9582252
## 6        0.8361215 9.406983 0.9582252
```

```
GY.stability <- GY.stability %>% group_by(env,Tester) %>% mutate(ScaledGY = scale(GY, center = TRUE, sca

GY.stability <- GY.stability %>% group_by(Inbred,Tester) %>% dplyr::mutate(Slope = coef(lm(ScaledGY ~ me

GY.stability <- as.data.frame(lapply(GY.stability, unlist))
GY.stability$Slope <- as.numeric(GY.stability$Slope)


result <- GY.stability %>%  group_by(env,Tester) %>% dplyr::mutate(Meann = mean(Predicted.ScaledGY)) %>%
result <- result %>% distinct(env,Tester, .keep_all = TRUE)
head(result)
```

```
##         Inbred       GY Tester       env     X window_size start_day end_day
## 1 W10004_0007 10.39559  PHK76 DEH1.2020   226           2       100     101
## 2 W10004_0007 12.45757  PHK76 DEH1.2021  8227           2       100     101
## 3 W10004_0007 10.80473  PHK76 IAH1.2021 16228           2       100     101
## 4 W10004_0007 12.04884  PHK76 IAH2.2021 24229           2       100     101
## 5 W10004_0007 12.16717  PHK76 IAH3.2021 32230           2       100     101
## 6 W10004_0007 11.76778  PHK76 IAH4.2021 40231           2       100     101
##   mean_temperature      Mean       cor  ScaledGY       Slope Intercept
## 1        0.8361215  9.406983 0.9582252 1.0576008 -0.08205393 0.9387461
## 2        0.9914683 11.611270 0.9582252 0.7582314 -0.08205393 0.9387461
## 3        0.7875916  9.866236 0.9582252 0.9700140 -0.08205393 0.9387461
## 4        0.9933251 11.465615 0.9582252 0.6833282 -0.08205393 0.9387461
## 5        0.9527715 11.319659 0.9582252 0.7458227 -0.08205393 0.9387461
## 6        0.8669274 10.809764 0.9582252 1.1312101 -0.08205393 0.9387461
##   Predicted.ScaledGY         Meann
## 1          0.8701390  7.089187e-17
```

```
## 2          0.8573922 -2.055545e-18
## 3          0.8741211  8.607148e-17
## 4          0.8572398 -6.220844e-18
## 5          0.8605674  1.315849e-17
## 6          0.8676113  4.745394e-17
```

```
p12<- ggplot(GY.stability, aes(x=mean_temperature, y=Predicted.ScaledGY, group=Inbred, color=Slope)) +
  geom_line(alpha=0.4) +
  # geom_smooth(se=F)+
  geom_point(alpha=0.3)+
  # geom_text( result, mapping=aes(label = Env, x=Mean, y=Meann-3), color='black', angle=90, size=1, al
  geom_point(result, mapping=aes(x=mean_temperature, y=2.48 ),color="black" ,shape=24,alpha=0.5   )+
  geom_text_repel(result,mapping=aes(x=mean_temperature, y=2.48, label=env ),color="black",size=2,
                  force_pull   = 0, # do not pull toward data points
                  nudge_y      = 0.05,
                  direction    = "x",
                  angle        = 90,
                  hjust        = 0,
                  segment.size = 0.2,
                  max.iter = 1e4, max.time = 1
  )+
  #scale_color_brewer(palette = "Dark2")+
  # geom_errorbar(aes(ymin=Mean-sd, ymax=Mean+sd), width=.2,
  #                position=position_dodge(0.05))+
  scale_y_continuous(name="Predicted GY")+
  theme(legend.position="right") +
  theme_bw()+
  facet_grid(~Tester,scales ="free_x")+
  scale_x_continuous(name = "Environmental index"  )+
  theme(#legend.background = element_blank(),
    legend.background =  element_rect(fill = alpha("gray80", 0.3) , size=0.2, linetype="solid"),
    legend.key.height = unit(0.5, 'cm'),
    panel.grid.major  = element_blank(),
    panel.grid.minor  = element_blank(),
    legend.key.size = unit(12,"pt"),
    axis.text.y = element_text(angle = 90, vjust=0.8,  hjust=0.5),
    legend.position   = c(0.75, 0.25))+
  scale_colour_gradient2("Slope", low = "navy", mid = "cyan", high = "red", midpoint = mean(GY.stabilit
#  guides(color = guide_legend(override.aes = list(size = 1,alpha = 1) ) )+
#  ggtitle("A) Slope of the temporal plant height across environments")

p12
```

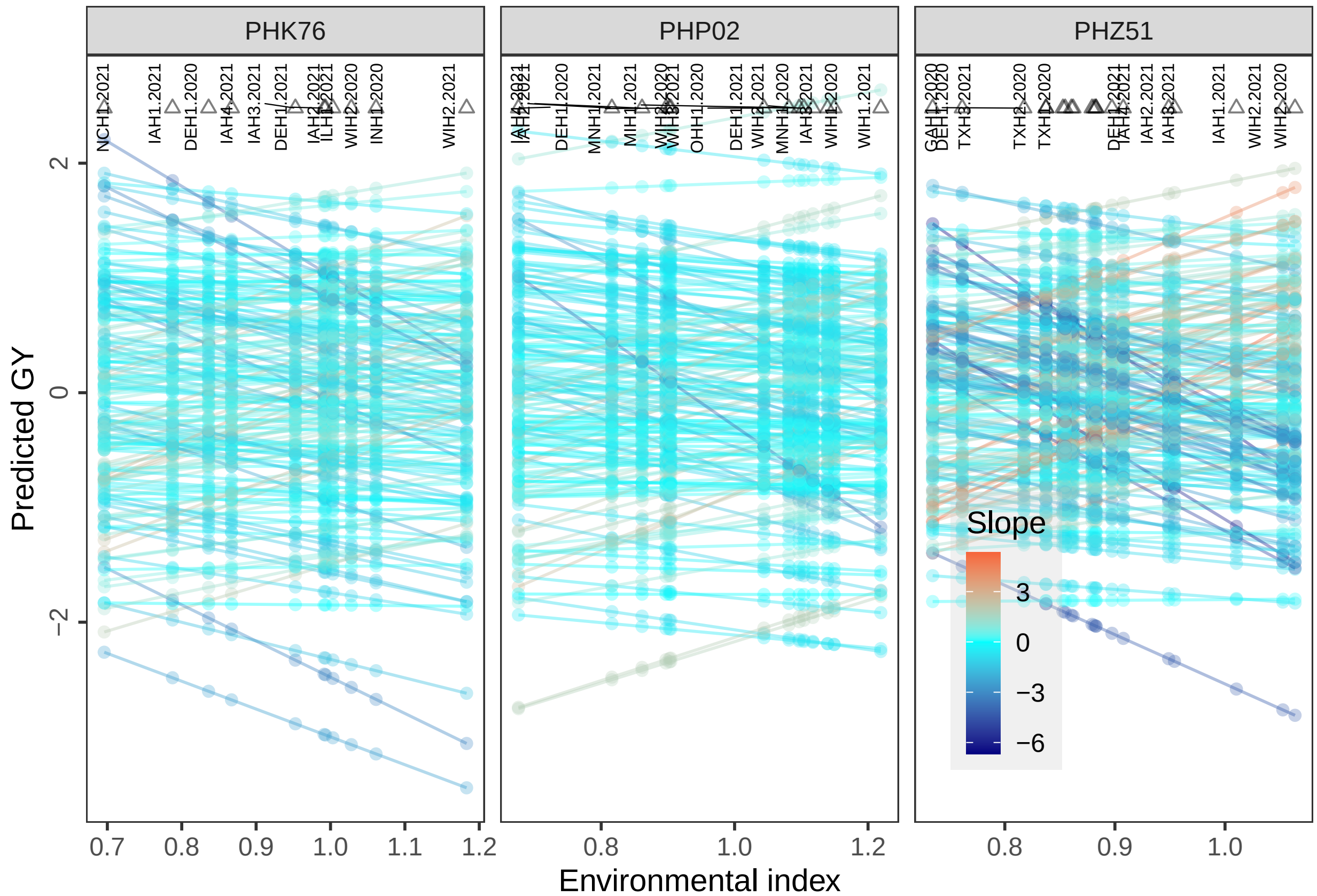

Genomic Prediction

Data Files Needed:

- All.Pred.Ability.reactionnorm.csv
- All.Pred.Ability.reactionnorm.Formatted.csv
- All.Pred.Ability.additive.csv
- All.Pred.Ability.additive.Formatted.csv

Code:

The scripts for the genomic prediction models (A and B) were run using the Texas A&M's High-Performance Research Computing (HPRC) due to the computational power required. The code for the genomic prediction models are not included within this RMarkdown file, but can be found in the same GitHub repository.

The code below uses the outputs of the genomic prediction models to make the prediction accuracy figures. They are split according to common plant breeding scenarios (CV0, CV00, CV1, and CV2) as found in (Jarquin, 2017).

```
pacman::p_load(readr, ggplot2, ggrepel)

git_path <- "https://raw.githubusercontent.com/fatmaoz25/ReactionNormGenomicPrediction/refs/heads/main"
```

```r
combined_data_file <- "All.Pred.Ability.additive.csv"
combined_data_path <- file.path(git_path, combined_data_file)
combined_data <- read.csv(combined_data_path)

df <- combined_data
df <- df %>% group_by(CV,Tester) %>% dplyr::summarise(mean =round(mean(cor),2) ,
                                                      sd =round(sd(cor),2)) %>% as.data.frame()

df$CV <- factor(df$CV ,levels = c("CV1", "CV2", "CV0", "CV00"))
df$facet <- df$CV
df$facet <- gsub("CV1", "Unested hybrids in \ntested environments", df$facet)
df$facet <- gsub("CV2", "Tested hybrids in \ntested environments", df$facet)
df$facet <- gsub("CV00", "Untested hybrids in \nuntested environments", df$facet)
df$facet <- gsub("CV0", "Tested hybrids in \nuntested environments", df$facet)

head(df)
```

```
##     CV Tester mean   sd                                      facet
## 1  CV0  PHK76 0.82 0.05   Tested hybrids in \nuntested environments
## 2  CV0  PHP02 0.78 0.06   Tested hybrids in \nuntested environments
## 3  CV0  PHZ51 0.65 0.09   Tested hybrids in \nuntested environments
## 4 CV00  PHK76 0.35 0.11 Untested hybrids in \nuntested environments
## 5 CV00  PHP02 0.34 0.12 Untested hybrids in \nuntested environments
## 6 CV00  PHZ51 0.27 0.12 Untested hybrids in \nuntested environments
```

```r
df$facet <- factor(df$facet ,levels = c("Tested hybrids in \ntested environments",
                                        "Unested hybrids in \ntested environments",
                                        "Tested hybrids in \nuntested environments",
                                        "Untested hybrids in \nuntested environments"))

p <- ggplot(df, aes(x=Tester, y=mean, fill=Tester)) +
  geom_bar(stat="identity", position=position_dodge()) +
  geom_text(aes(label=paste(sprintf("%.2f", mean),  "\u00B1",  sprintf("%.2f", sd), sep=" "),   y=0.12)
          position=position_dodge(0.9),  angle=90, color="black", size=3.5)+
  geom_errorbar(aes(ymin=mean, ymax=mean+sd), width=.2,
                position=position_dodge(.9)) +
  facet_wrap(~facet)+
  scale_y_continuous("Prediction ability")+
  scale_x_discrete("Population")+
  scale_fill_brewer(palette="Set2")+
  theme(legend.position = c(0.9,0.35),
        legend.background = element_blank())
p
```

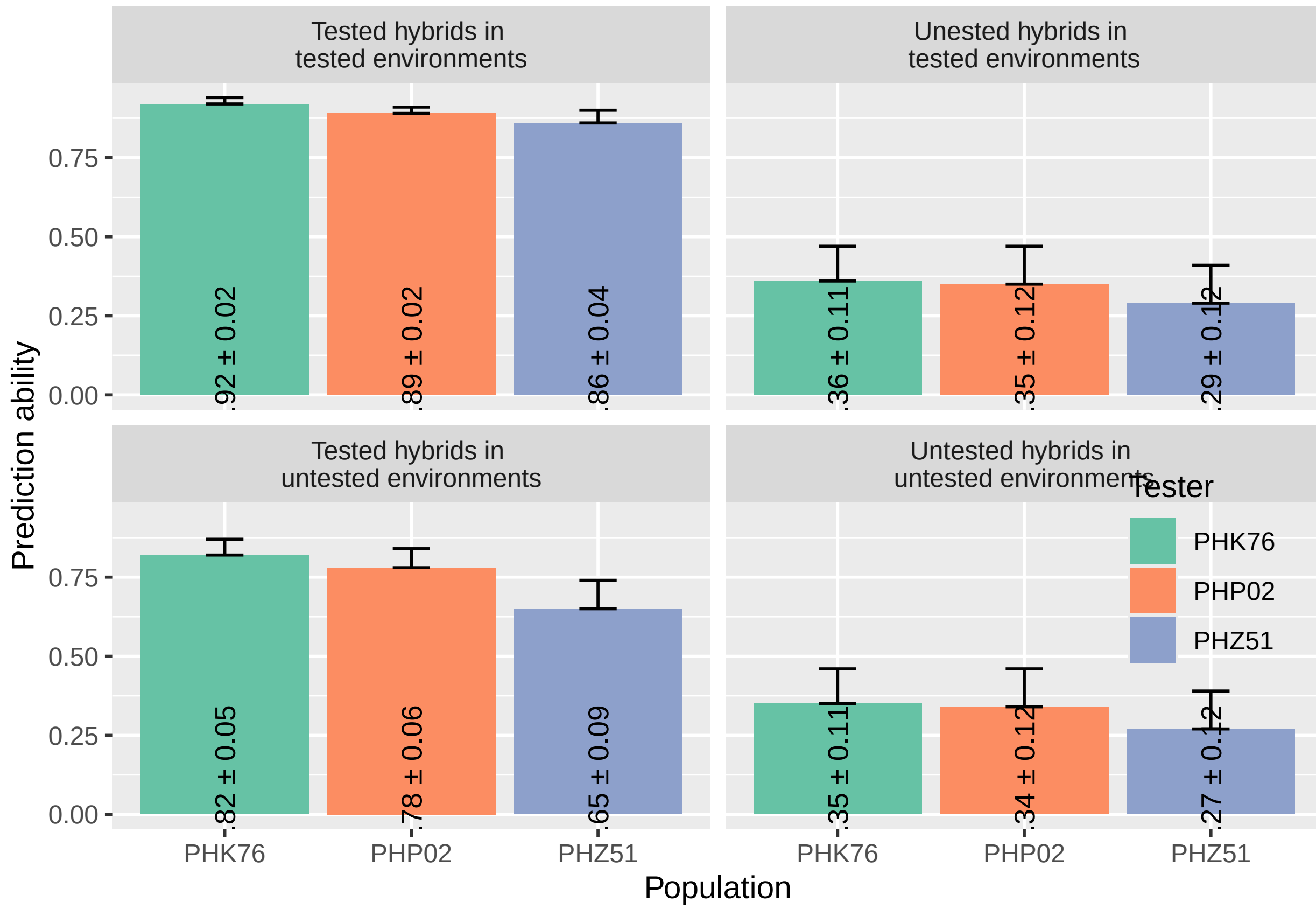


```
pacman::p_load(readr, ggplot2, ggrepel)

git_path <- "https://raw.githubusercontent.com/fatmaoz25/ReactionNormGenomicPrediction/refs/heads/main"

combined_data_file <- "All.Pred.Ability.reactionnorm.csv"
combined_data_path <- file.path(git_path, combined_data_file)
combined_data <- read.csv(combined_data_path)

df <- combined_data
df <- df %>% group_by(CV,Tester) %>% dplyr::summarise(mean =round(mean(cor),2) ,
                                                        sd =round(sd(cor),2)) %>% as.data.frame()

df$CV <- factor(df$CV ,levels = c("CV1", "CV2", "CV0", "CV00"))
df$facet <- df$CV
df$facet <- gsub("CV1", "Unested hybrids in \ntested environments", df$facet)
df$facet <- gsub("CV2", "Tested hybrids in \ntested environments", df$facet)
df$facet <- gsub("CV00", "Untested hybrids in \nuntested environments", df$facet)
df$facet <- gsub("CV0", "Tested hybrids in \nuntested environments", df$facet)

head(df)
```

```
##      CV Tester mean   sd                                   facet
## 1   CV0  phk76 0.74 0.07   Tested hybrids in \nuntested environments
## 2   CV0  php02 0.70 0.07   Tested hybrids in \nuntested environments
## 3   CV0  phz51 0.62 0.08   Tested hybrids in \nuntested environments
## 4  CV00  phk76 0.36 0.12 Untested hybrids in \nuntested environments
```

```
## 5 CV00  php02 0.39 0.11 Untested hybrids in \nuntested environments
## 6 CV00  phz51 0.26 0.13 Untested hybrids in \nuntested environments
```

```r
df$facet <- factor(df$facet ,levels = c("Tested hybrids in \ntested environments",
                                        "Unested hybrids in \ntested environments",
                                        "Tested hybrids in \nuntested environments",
                                        "Untested hybrids in \nuntested environments"))

p <- ggplot(df, aes(x=Tester, y=mean, fill=Tester)) +
  geom_bar(stat="identity", position=position_dodge()) +
  geom_text(aes(label=paste(sprintf("%.2f", mean),  "\u00B1",  sprintf("%.2f", sd), sep=" "),    y=0.12)
          position=position_dodge(0.9),  angle=90, color="black", size=3.5)+
  geom_errorbar(aes(ymin=mean, ymax=mean+sd), width=.2,
              position=position_dodge(.9)) +
  facet_wrap(~facet)+
  scale_y_continuous("Prediction ability")+
  scale_x_discrete("Population")+
  scale_fill_brewer(palette="Set2")+
  theme(legend.position = c(0.9,0.35),
        legend.background = element_blank())
p
```

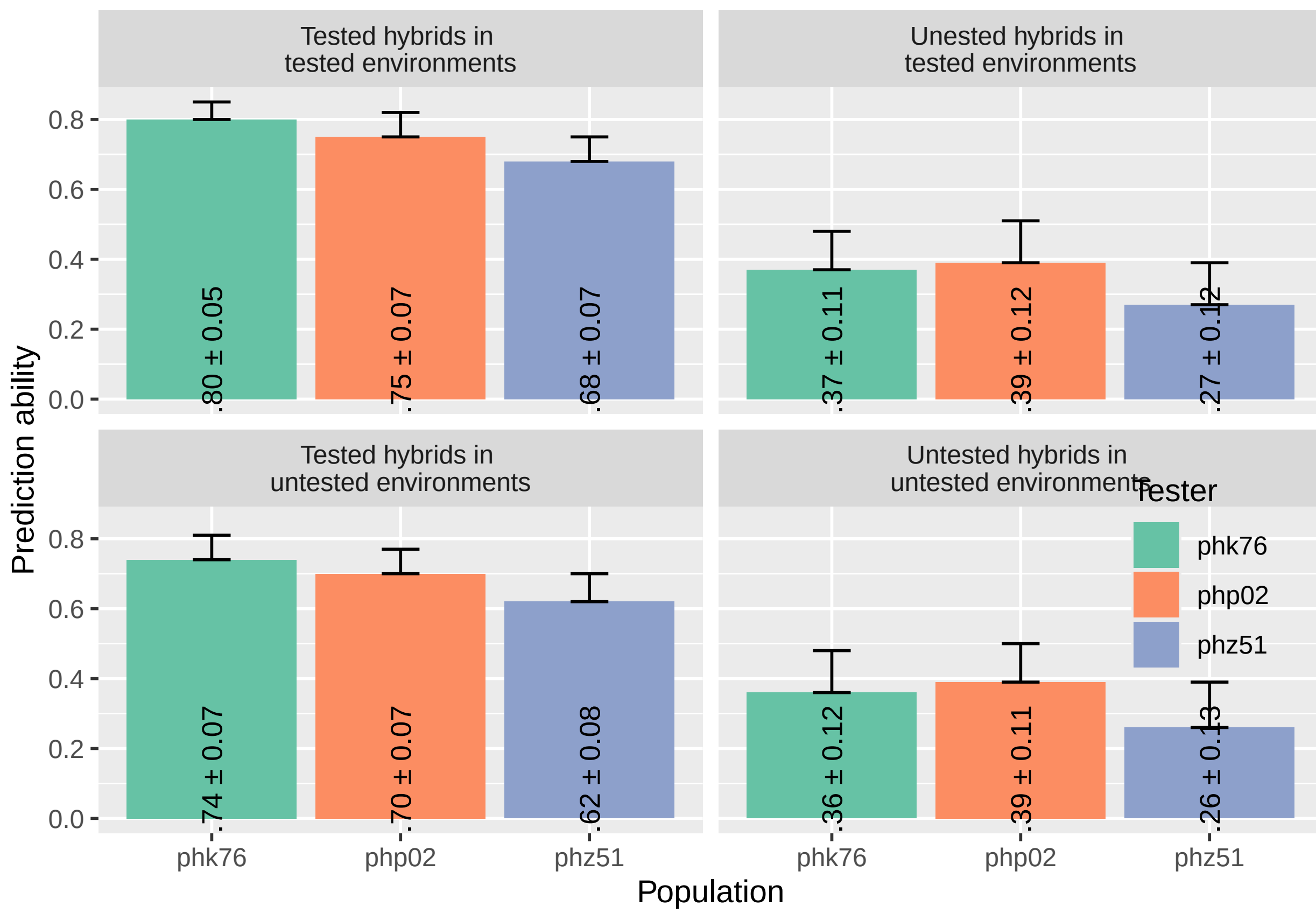

Supplement: Supplementary file 1 [file TPG2-18-e70078-s002.pdf]
