# Supplementary material for: Phenotypic plasticity in maize grain yield: Genetic and environmental insights of response to environmental gradients
Source: Plant Genome. 2025 Aug 7;18(3):e70078. doi: 10.1002/tpg2.70078 (PMC12332327; doi:10.1002/tpg2.70078)
Supplement: Supplementary file 1 — Supplemental Table 1 (S1) contains the significant quantitative trait loci (QTLs) discovered using a LOD threshold of 5, along with candidate genes and their functions for grain yield for three different maize tester populations. Supplemental Table 2 (S2) contains the significant quantitative trait loci (QTLs) discovered using a LOD threshold of 5, along with candidate genes and their functions for grain yield slope and intercept for the same three populations as above. [file TPG2-18-e70078-s001.docx]

**Phenotypic Plasticity in Maize Grain Yield: Genetic and Environmental Insights of Response to Environmental Gradients**

Fatma Ozair, Alper Adak, Seth C. Murray^*^, Ryan T. Alpers, Alejandro C. Aviles, Dayane C. Lima, Jode Edwards, David Ertl, Michael A. Gore, Candice N. Hirsch, Joseph E. Knoll, James C. Schnable, Maninder P. Singh, Erin E. Sparks, Addie Thompson, Teclemariam Weldekidan, Wenwei Xu

*Correspondence: Seth C. Murray, Department of Soil and Crop Sciences, Texas A&M University, College Station, TX, 77843-2474, USA

Number of Pages: 77

Number of Tables: 2

**Supplemental Table 1 (S1):** all quantitative trait loci (QTLs) discovered along with candidate genes and their functions for grain yield for three different maize populations with PHK76, PHP02 and PHZ51 testers.

| Tester | Env | Chr | Start (Mbp) | End (Mbp) | Peak (Mbp) | Peak LOD (Mbp) | No. Genes in Interval | Gene | Function |
| --- | --- | --- | --- | --- | --- | --- | --- | --- | --- |
| PHK76 | DEH1.2020 | 2 | 46101662 | 46183987 | 46102107 | 5.495422099 | 6 |  |  |
|  |  |  |  |  |  |  |  | Zm00001eb081370 (46101548..46107263) |  |
|  |  |  |  |  |  |  |  | Zm00001eb081390 (46139379..46140506) | Uncharacterized protein |
|  |  |  |  |  |  |  |  | Zm00001eb081400 (46142186..46148588 ) | Methyltransferase |
|  |  |  |  |  |  |  |  | Zm00001eb081380 (46132759..46135087) | Serine hydrolase FSH domain-containing protein |
|  |  |  |  |  |  |  |  | Zm00001eb081410 (46152271..46158082) | Signal recognition particle subunit SRP68 |
|  |  |  |  |  |  |  |  | Zm00001eb081420 (46179764..46184123) | Steroid nuclear receptor ligand-binding |
| PHK76 | DEH1.2020 | 3 | 190197719 | 190205506 | 190204793 | 5.201695229 | 1 |  |  |
|  |  |  |  |  |  |  |  | Zm00001eb149600 (190197068..190205511) | Putative WRKY transcription factor 30 |
| PHK76 | DEH1.2020 | 8 | 169059843 | 169065987 | 169062120 | 5.142706631 | 3 |  |  |
|  |  |  |  |  |  |  |  | Zm00001eb364920 (169058676..169059931) | Stomagen C-terminal domain-containing protein |
|  |  |  |  |  |  |  |  | Zm00001eb364930 (169061391..169062240) | Bifunctional inhibitor/plant lipid transfer protein/seed storage helical domain-containing protein |
|  |  |  |  |  |  |  |  | Zm00001eb364940 (169065441..169066013) | PVR3-like protein (Protease inhibitor/seed storage/LTP family protein) |
| PHK76 | DEH1.2021 | 2 | 32769198 | 35051204 | 33489553 | 6.355692708 | 53 |  |  |
|  |  |  |  |  |  |  |  | Zm00001eb078120 (32768952..32788400) | Major facilitator superfamily protein; Nodulin-like domain-containing protein |
|  |  |  |  |  |  |  |  | Zm00001eb078130 (32839664..32842500) | 40S ribosomal protein S11 N-terminal domain-containing protein |
|  |  |  |  |  |  |  |  | Zm00001eb078140 (32870011..32878172) | Trafficking protein particle complex II-specific subunit 120-like protein |
|  |  |  |  |  |  |  |  | Zm00001eb078150 (32878800..32879662) | t-SNARE coiled-coil homology domain-containing protein |
|  |  |  |  |  |  |  |  | Zm00001eb078160 (32918154..32919348) | HMA domain-containing protein |
|  |  |  |  |  |  |  |  | Zm00001eb078170 (33011767..3301826) | Sister chromatid cohesion protein PDS5 B-B |
|  |  |  |  |  |  |  |  | Zm00001eb078190 (33151454..33154775) | DNA glycosylase superfamily protein; DNA-3-methyladenine glycosylase I |
|  |  |  |  |  |  |  |  | Zm00001eb078200 (33157327..33162559) | Magnesium transporter |
|  |  |  |  |  |  |  |  | Zm00001eb078210 (33207589..33209622) | Ribosomal protein L23/L25 N-terminal domain-containing protein; 60S ribosomal protein L23a (60S ribosomal protein L23a-1) |
|  |  |  |  |  |  |  |  | Zm00001eb078220 (33379891..33386009) | Shikimate O-hydroxycinnamoyltransferase |
|  |  |  |  |  |  |  |  | Zm00001eb078230 (33439003..33439593) | Uncharacterized protein |
|  |  |  |  |  |  |  |  | Zm00001eb078240 (33440386..33440793) | Uncharacterized protein |
|  |  |  |  |  |  |  |  | Zm00001eb078250 (33442427..33443391) | Zinc finger CCCH domain-containing protein 22 |
|  |  |  |  |  |  |  |  | Zm00001eb078280 (33485604..33486772) | GPI-anchored protein |
|  |  |  |  |  |  |  |  | Zm00001eb078290 (33487363..33489618) | Cytochrome P450 71A26 |
|  |  |  |  |  |  |  |  | Zm00001eb078300 (33490730..33498659) | Tubulin/FtsZ GTPase domain-containing protein |
|  |  |  |  |  |  |  |  | Zm00001eb078330 (33592210..33596785) | Uncharacterized protein |
|  |  |  |  |  |  |  |  | Zm00001eb078340 (33600173..33602240) | Cytochrome P450 CYP71C36 |
|  |  |  |  |  |  |  |  | Zm00001eb078350 (33817057..33822483) | Eukaryotic translation initiation factor isoform 4G-2 |
|  |  |  |  |  |  |  |  | Zm00001eb078360 (33827014..33830293) | Enhancer of rudimentary homolog |
|  |  |  |  |  |  |  |  | Zm00001eb078370 (33831377..33835361) | Folate-biopterin transporter 2 |
|  |  |  |  |  |  |  |  | Zm00001eb078380 (33834954..33835361) | Pre-mRNA-splicing factor 18 |
|  |  |  |  |  |  |  |  | Zm00001eb078390 (33897655..33900394) | N-acetyltransferase ESCO1 (Protein CHROMOSOME TRANSMISSION FIDELITY 7) |
|  |  |  |  |  |  |  |  | Zm00001eb078400 (33969079..33972745) | Protein CHROMOSOME TRANSMISSION FIDELITY 7 |
|  |  |  |  |  |  |  |  | Zm00001eb078410 (34008734..34012060) | Protein CHROMOSOME TRANSMISSION FIDELITY 7 |
|  |  |  |  |  |  |  |  | Zm00001eb078420 (34022858..34023375) |  |
|  |  |  |  |  |  |  |  | Zm00001eb078430 (34025361..34026186) |  |
|  |  |  |  |  |  |  |  | Zm00001eb078440 (34034310..34037394) | S-adenosylmethionine decarboxylase proenzyme |
|  |  |  |  |  |  |  |  | Zm00001eb078470 (34058765..34061601) | VAN3-binding protein |
|  |  |  |  |  |  |  |  | Zm00001eb078480 (34063699..34065949) | OSJNBa0067K08.20-like protein |
|  |  |  |  |  |  |  |  | Zm00001eb078450 (34039586..34041412) | 14-3-3 domain-containing protein |
|  |  |  |  |  |  |  |  | Zm00001eb078490 (34132741..34134680) | Zinc finger protein CONSTANS-LIKE 3; B box-type domain-containing protein; Zinc finger protein CONSTANS-LIKE 5 |
|  |  |  |  |  |  |  |  | Zm00001eb078500 (34182812..34184501) | Uncharacterized protein |
|  |  |  |  |  |  |  |  | Zm00001eb078510 (34182975..34184501) | Ribonucleoside-diphosphate reductase small chain |
|  |  |  |  |  |  |  |  | Zm00001eb078520 (34300366..34330816) | Mitochondrial proton/calcium exchanger protein |
|  |  |  |  |  |  |  |  | Zm00001eb078530 (34390694..34400773) | CBF1 interacting corepressor; CBF1-interacting co-repressor CIR N-terminal domain-containing protein |
|  |  |  |  |  |  |  |  | Zm00001eb078540 (34393432..34395082) | Protein TRANSPARENT TESTA 16 |
|  |  |  |  |  |  |  |  | Zm00001eb078550 (34396537..34397759) | SAC domain-containing protein |
|  |  |  |  |  |  |  |  | Zm00001eb078560 (34500328..34501166) | Uncharacterized protein |
|  |  |  |  |  |  |  |  | Zm00001eb078570 (34532823..34536576) | NAD(P)-binding Rossmann-fold superfamily protein |
|  |  |  |  |  |  |  |  | Zm00001eb078580 (34589537..34591295) | Uncharacterized protein |
|  |  |  |  |  |  |  |  | Zm00001eb078590 (34638114..34642950) | Protein NLP2; NLP transcription factor |
|  |  |  |  |  |  |  |  | Zm00001eb078600 (34741485..34745044) | Uncharacterized protein; Homeodomain-like transcription factor superfamily protein; Myb-like DNA-binding domain containing protein |
|  |  |  |  |  |  |  |  | Zm00001eb078620 (34901510..34906207) | BZIP domain-containing protein; BZIP transcription factor |
|  |  |  |  |  |  |  |  | Zm00001eb078630 (34905463..34906179) | Uncharacterized protein |
|  |  |  |  |  |  |  |  | Zm00001eb078640 (34908223..34911194) | Cytochrome b561 and DOMON domain-containing protein |
|  |  |  |  |  |  |  |  | Zm00001eb078650 (34915636..34916808) | Uncharacterized protein |
|  |  |  |  |  |  |  |  | Zm00001eb078660 (34947815..34950074) | DUF642 domain-containing protein |
|  |  |  |  |  |  |  |  | Zm00001eb078670 (34988811..34991020) | DUF642 domain-containing protein |
|  |  |  |  |  |  |  |  | Zm00001eb078680 (35009614..35011926) | DUF642 domain-containing protein |
|  |  |  |  |  |  |  |  | Zm00001eb078690 (35013165..35015203) | DUF642 domain-containing protein |
|  |  |  |  |  |  |  |  | Zm00001eb078700 (35049354..35051232) | DUF642 domain-containing protein |
|  |  |  |  |  |  |  |  | Zm00001eb078710 (35053788..35056702) | DUF642 domain-containing protein |
| PHK76 | IAH1.2021 | 2 | 68678727 | 69438828 | 68995922 | 5.533531592 | 8 |  |  |
|  |  |  |  |  |  |  |  | Zm00001eb085110 (68678720..68689329) | Zinc finger CCCH domain-containing protein 41; Zinc finger CCCH domain-containing protein 27 |
|  |  |  |  |  |  |  |  | Zm00001eb085130 (68906606..68924826) | dihydrolipoyllysine-residue succinyltransferase |
|  |  |  |  |  |  |  |  | Zm00001eb085140 (68947960..68948316) | Uncharacterized protein |
|  |  |  |  |  |  |  |  | Zm00001eb085150 (68991625..68996526) | glycerophosphodiester phosphodiesterase |
|  |  |  |  |  |  |  |  | Zm00001eb085160 (68995633..68996332) | Uncharacterized protein |
|  |  |  |  |  |  |  |  | Zm00001eb085170 (69028695..69033776) | Serine/threonine-protein kinase NAK (Serine/threonine-protein kinase PBS1) |
|  |  |  |  |  |  |  |  | Zm00001eb085180 (69194339..69194959) | Uncharacterized protein |
|  |  |  |  |  |  |  |  | Zm00001eb085190 (69437920..69438968) | Grx_C8-glutaredoxin subgroup III |
| PHK76 | IAH1.2021 | 3 | 2645638 | 2756062 | 2756062 | 5.412939597 | 4 |  |  |
|  |  |  |  |  |  |  |  | Zm00001eb119530 (2645358..2649208) | Flavin-containing monooxygenase |
|  |  |  |  |  |  |  |  | Zm00001eb119540 (2682223..2684039) | Ethylene-responsive transcription factor CRF4 |
|  |  |  |  |  |  |  |  | Zm00001eb119560 (2750418..2756485) | Uncharacterized protein |
|  |  |  |  |  |  |  |  | Zm00001eb119570 (2750922..2756587) | Leucine-rich repeat-containing N-terminal plant-type domain-containing protein |
|  |  |  |  |  |  |  |  |  |  |
| PHK76 | IAH2.2021 | 2 | 31776899 | 34133822 | 33489553 | 5.869092542 | 53 |  |  |
|  |  |  |  |  |  |  |  | Zm00001eb077880 (31776650..31779535) | GDSL esterase/lipase LTL1 |
|  |  |  |  |  |  |  |  | Zm00001eb077890 (31817305..31823510) | NADP-dependent oxidoreductase domain-containing protein; Pyridoxal reductase chloroplastic |
|  |  |  |  |  |  |  |  | Zm00001eb077900 (31864886..31889610) | CLIP-associated protein; TOG domain-containing protein |
|  |  |  |  |  |  |  |  | Zm00001eb077910 (31909705..31911368) | "Protein LOW PSII ACCUMULATION 1, chloroplastic" |
|  |  |  |  |  |  |  |  | Zm00001eb077920 (31917546..31924058) | Phosphatidylinositol N-acetyglucosaminlytransferase subunit P-related |
|  |  |  |  |  |  |  |  | Zm00001eb077930 (31941048..31942918) | Beta-16-galactosyltransferase GALT29A |
|  |  |  |  |  |  |  |  | Zm00001eb077940 (31947441..31967471) | Brf1 TBP-binding domain-containing protein |
|  |  |  |  |  |  |  |  | Zm00001eb077950 (32049709..32050345) | Peptidase A1 domain-containing protein |
|  |  |  |  |  |  |  |  | Zm00001eb077960 (32100148..32104617) | Serinc-domain containing serine and sphingolipid biosynthesis protein |
|  |  |  |  |  |  |  |  | Zm00001eb077980 (32153012..32158462) | Putative leucine-rich repeat receptor-like protein kinase |
|  |  |  |  |  |  |  |  | Zm00001eb077990 (32158225..32160541) | Glycosyltransferase |
|  |  |  |  |  |  |  |  | Zm00001eb078000 (32377642..32383391) | COI1 F-box domain-containing protein |
|  |  |  |  |  |  |  |  | Zm00001eb078010 (32384392..32386323) | Protein binding protein |
|  |  |  |  |  |  |  |  | Zm00001eb078030 (32440956..32441979) | Protein kinase domain-containing protein |
|  |  |  |  |  |  |  |  | Zm00001eb078040 (32496667..32502295) | Polyadenylate-binding protein |
|  |  |  |  |  |  |  |  | Zm00001eb078050 (32531848..32532839) | Uncharacterized protein |
|  |  |  |  |  |  |  |  | Zm00001eb078060 (32531921..32532902) | Uncharacterized protein |
|  |  |  |  |  |  |  |  | Zm00001eb078070 (32533687..32539866) | adenine phosphoribosyltransferase |
|  |  |  |  |  |  |  |  | Zm00001eb078080 (32604827..32609284) | "Dol-P-Glc:Glc(2)Man(9)GlcNAc(2)-PP-Dol alpha-1, 2-glucosyltransferase" |
|  |  |  |  |  |  |  |  | Zm00001eb078090 (32609680..32614262) | Protein kinase domain-containing protein |
|  |  |  |  |  |  |  |  | Zm00001eb078100 (32714200..32723073) | Leucine-rich repeat family protein |
|  |  |  |  |  |  |  |  | Zm00001eb078110 (32729608..32737169) | Calcium-binding EF hand family protein; EF-hand domain-containing protein |
|  |  |  |  |  |  |  |  | Zm00001eb078120 (32768952..32788400) | Major facilitator superfamily protein; Nodulin-like domain-containing protein |
|  |  |  |  |  |  |  |  | Zm00001eb078130 (32839664..32842500) | 40S ribosomal protein S11 N-terminal domain-containing protein |
|  |  |  |  |  |  |  |  | Zm00001eb078140 (32870011..32878172) | Trafficking protein particle complex II-specific subunit 120-like protein |
|  |  |  |  |  |  |  |  | Zm00001eb078150 (32878800..32879662) | t-SNARE coiled-coil homology domain-containing protein |
|  |  |  |  |  |  |  |  | Zm00001eb078160 (32918154..32919348) | HMA domain-containing protein |
|  |  |  |  |  |  |  |  | Zm00001eb078170 (33011767..33018261) | Sister chromatid cohesion protein PDS5 B-B |
|  |  |  |  |  |  |  |  | Zm00001eb078190 (33151454..33154775) | DNA glycosylase superfamily protein; DNA-3-methyladenine glycosylase I |
|  |  |  |  |  |  |  |  | Zm00001eb078200 (33157327..33162559) | Magnesium transporter |
|  |  |  |  |  |  |  |  | Zm00001eb078210 (33207589..33209622) | Ribosomal protein L23/L25 N-terminal domain-containing protein; 60S ribosomal protein L23a |
|  |  |  |  |  |  |  |  | Zm00001eb078220 (33379891..33386009) | Shikimate O-hydroxycinnamoyltransferase |
|  |  |  |  |  |  |  |  | Zm00001eb078230 (33439003..33439593) | Uncharacterized protein |
|  |  |  |  |  |  |  |  | Zm00001eb078240 (33440386..33440793) | Uncharacterized protein |
|  |  |  |  |  |  |  |  | Zm00001eb078250 (33442427..33443391) | Zinc finger CCCH domain-containing protein 22 |
|  |  |  |  |  |  |  |  | Zm00001eb078280 (33485604..33486772) | GPI-anchored protein |
|  |  |  |  |  |  |  |  | Zm00001eb078290 (33487363..33489618) | Cytochrome P450 71A26 |
|  |  |  |  |  |  |  |  | Zm00001eb078300 (33490730..33498659) | Tubulin/FtsZ GTPase domain-containing protein |
|  |  |  |  |  |  |  |  | Zm00001eb078330 (33592210..33596785) | Uncharacterized protein |
|  |  |  |  |  |  |  |  | Zm00001eb078340 (33600173..33602240) | Cytochrome P450 CYP71C36 |
|  |  |  |  |  |  |  |  | Zm00001eb078350 (33817057..33822483) | Eukaryotic translation initiation factor isoform 4G-2 |
|  |  |  |  |  |  |  |  | Zm00001eb078360 (33827014..33830293) | Enhancer of rudimentary homolog |
|  |  |  |  |  |  |  |  | Zm00001eb078370 (33831377..33835361) | Folate-biopterin transporter 2 |
|  |  |  |  |  |  |  |  | Zm00001eb078380 (33834954..33835361) | Pre-mRNA-splicing factor 18 |
|  |  |  |  |  |  |  |  | Zm00001eb078390 (33897655..33900394) | N-acetyltransferase ESCO1 |
|  |  |  |  |  |  |  |  | Zm00001eb078400 (33969079..33972745) | Protein CHROMOSOME TRANSMISSION FIDELITY 7 |
|  |  |  |  |  |  |  |  | Zm00001eb078420 (34022858..34023375) |  |
|  |  |  |  |  |  |  |  | Zm00001eb078430 (34025361..34026186) |  |
|  |  |  |  |  |  |  |  | Zm00001eb078440 (34034310..34037394) | S-adenosylmethionine decarboxylase proenzyme |
|  |  |  |  |  |  |  |  | Zm00001eb078470 (34058765..34061601) | VAN3-binding protein |
|  |  |  |  |  |  |  |  | Zm00001eb078480 (34063699..34065949) | OSJNBa0067K08.20-like protein |
|  |  |  |  |  |  |  |  | Zm00001eb078450 (34039586..34041412) | 14-3-3 domain-containing protein |
|  |  |  |  |  |  |  |  | Zm00001eb078490 (34132741..34134680) | Zinc finger protein CONSTANS-LIKE 3 |
| PHK76 | IAH2.2021 | 3 | 191059861 | 191061865 | 191059861 | 5.033772683 | 1 |  |  |
|  |  |  |  |  |  |  |  | Zm00001eb149760 (191058968..191062657) | Calcium uniporter protein |
| PHK76 | IAH3.2021 | 2 | 36122371 | 36123549 | 36123549 | 5.004465954 | 1 |  |  |
|  |  |  |  |  |  |  |  | Zm00001eb078880 (36121944..36124802) | Protein kinase domain-containing protein |
| PHK76 | IAH4.2021 | 2 | 46067701 | 49247636 | 47586831 | 7.142483829 | 73 |  |  |
|  |  |  |  |  |  |  |  | Zm00001eb081350 (46065253..46067983) | Pentatricopeptide repeat-containing protein mitochondrial |
|  |  |  |  |  |  |  |  | Zm00001eb081360 (46077412..46079440) | Serine hydrolase FSH domain-containing protein |
|  |  |  |  |  |  |  |  | Zm00001eb081370 (46101548..46107263) |  |
|  |  |  |  |  |  |  |  | Zm00001eb081380 (46132759..46135087) | Serine hydrolase FSH domain-containing protein |
|  |  |  |  |  |  |  |  | Zm00001eb081390 (46139379..46140506) | Uncharacterized protein |
|  |  |  |  |  |  |  |  | Zm00001eb081400 (46142186..46148588) | Methyltransferase |
|  |  |  |  |  |  |  |  | Zm00001eb081430 (46187668..46192641) | protein-serine/threonine phosphatase |
|  |  |  |  |  |  |  |  | Zm00001eb081450 (46237772..46240437) | Mitochondrial uncoupling protein 3 |
|  |  |  |  |  |  |  |  | Zm00001eb081460 (46241585..46250478) | "Zeaxanthin epoxidase, chloroplastic " |
|  |  |  |  |  |  |  |  | Zm00001eb081410 (46152271..46158082) | Signal recognition particle subunit SRP68 |
|  |  |  |  |  |  |  |  | Zm00001eb081420 (46179764..46184123) | Steroid nuclear receptor ligand-binding |
|  |  |  |  |  |  |  |  | Zm00001eb081470 (46245692..46246490) | "Zeaxanthin epoxidase, chloroplastic |
|  |  |  |  |  |  |  |  | Zm00001eb081480 (46273740..46275698) | Eukaryotic aspartyl protease family protein |
|  |  |  |  |  |  |  |  | Zm00001eb081510 (46328883..46338027) | Uncharacterized protein |
|  |  |  |  |  |  |  |  | Zm00001eb081440 (46235545..46237787) | Fucosyltransferase |
|  |  |  |  |  |  |  |  | Zm00001eb081500 (46327760..46328442) | Uncharacterized protein |
|  |  |  |  |  |  |  |  | Zm00001eb081520 (46397819..46399664) | Uncharacterized protein |
|  |  |  |  |  |  |  |  | Zm00001eb081530 (46497812..46499976) | F11F12.5 protein |
|  |  |  |  |  |  |  |  | Zm00001eb081550 (46555608..46558022) | Glutamate decarboxylase |
|  |  |  |  |  |  |  |  | Zm00001eb081570 (46603332..46605515) | Aldose reductase (NAD(P)-linked oxidoreductase superfamily protein); NADP-dependent oxidoreductase domain-containing protein |
|  |  |  |  |  |  |  |  | Zm00001eb081540 (46545338..46555087) | Transcription repressor |
|  |  |  |  |  |  |  |  | Zm00001eb081560 (46597879..46598210) | Uncharacterized protein |
|  |  |  |  |  |  |  |  | Zm00001eb081580 (46657772..46664387) | NAD(P)-linked oxidoreductase superfamily protein |
|  |  |  |  |  |  |  |  | Zm00001eb081590 (46749618..46753776) | Serine carboxypeptidase-like 19 |
|  |  |  |  |  |  |  |  | Zm00001eb081600 (46786871..46789431) | Glutamate decarboxylase |
|  |  |  |  |  |  |  |  | Zm00001eb081610 (47030447..47035081) | Lipoxygenase |
|  |  |  |  |  |  |  |  | Zm00001eb081620 (47033910..47034921) | Uncharacterized protein |
|  |  |  |  |  |  |  |  | Zm00001eb081630 (47048631..47053049) | Pentatricopeptide repeat-containing protein |
|  |  |  |  |  |  |  |  | Zm00001eb081640 (47254140..47255433) | Uncharacterized protein |
|  |  |  |  |  |  |  |  | Zm00001eb081650 (47334562..47340256) | Signal peptidase complex subunit 2 |
|  |  |  |  |  |  |  |  | Zm00001eb081660 (47340250..47342830) | OBP3-responsive gene 4 |
|  |  |  |  |  |  |  |  | Zm00001eb081670 (47533650..47569713) | Uncharacterized protein |
|  |  |  |  |  |  |  |  | Zm00001eb081690 (47583409..47584109) | Uncharacterized protein |
|  |  |  |  |  |  |  |  | Zm00001eb081680 (47570358..47578628) | Pentatricopeptide repeat-containing protein |
|  |  |  |  |  |  |  |  | Zm00001eb081700 (47586389..47594455) | U6 snRNA-associated Sm-like protein LSm1 |
|  |  |  |  |  |  |  |  | Zm00001eb081710 (47588950..47589632) | Uncharacterized protein |
|  |  |  |  |  |  |  |  | Zm00001eb081720 (47595159..47597455) | Trihelix transcription factor ASIL2 |
|  |  |  |  |  |  |  |  | Zm00001eb081740 (47754596..47755860) | ABC transporter domain-containing protein |
|  |  |  |  |  |  |  |  | Zm00001eb081750 (47768705..47769509) | RmlC-like cupins superfamily protein |
|  |  |  |  |  |  |  |  | Zm00001eb081760 (47770487..47773701) | Uncharacterized protein |
|  |  |  |  |  |  |  |  | Zm00001eb081770 (47778184..47779308) | 22.0 kDa heat shock protein |
|  |  |  |  |  |  |  |  | Zm00001eb081780 (47798316..47802673) | Potassium channel |
|  |  |  |  |  |  |  |  | Zm00001eb081790 (47801197..47813654) | Putative translation initiation factor IF-2 |
|  |  |  |  |  |  |  |  | Zm00001eb081800 (47871801..47875027) | Uncharacterized protein |
|  |  |  |  |  |  |  |  | Zm00001eb081810 (47978176..48001319) | Ferric reduction oxidase 7 chloroplastic |
|  |  |  |  |  |  |  |  | Zm00001eb081840 (48018774..48019227) | Uncharacterized protein |
|  |  |  |  |  |  |  |  | Zm00001eb081850 (48026292..48028099) | S-adenosyl-L-methionine-dependent methyltransferase superfamily protein |
|  |  |  |  |  |  |  |  | Zm00001eb081860 (48108946..48110031) | Uncharacterized protein |
|  |  |  |  |  |  |  |  | Zm00001eb081870 (48126197..48132785) | PCI domain-containing protein |
|  |  |  |  |  |  |  |  | Zm00001eb081880 (48196829..48203589) | PCI domain-containing protein |
|  |  |  |  |  |  |  |  | Zm00001eb081890 (48263555..48266221) | Uncharacterized protein |
|  |  |  |  |  |  |  |  | Zm00001eb081900 (48302544..48307701) | MLO-like protein |
|  |  |  |  |  |  |  |  | Zm00001eb081930 (48388882..48402806) | Calmodulin-binding protein 60 |
|  |  |  |  |  |  |  |  | Zm00001eb081910 (48381263..48381938) | Nucleoside diphosphate kinase |
|  |  |  |  |  |  |  |  | Zm00001eb081920 (48382057..48383013) | CCT motif family protein |
|  |  |  |  |  |  |  |  | Zm00001eb081940 (48448135..48449169) | C2H2-type domain-containing protein |
|  |  |  |  |  |  |  |  | Zm00001eb081950 (48451637..48452203) | Late embryogenesis abundant protein |
|  |  |  |  |  |  |  |  | Zm00001eb081960 (48453863..48454429) | Late embryogenesis abundant protein |
|  |  |  |  |  |  |  |  | Zm00001eb081970 (48456087..48456653) | Late embryogenesis abundant protein |
|  |  |  |  |  |  |  |  | Zm00001eb081980 (48621818..48623259) | RING-type E3 ubiquitin transferase |
|  |  |  |  |  |  |  |  | Zm00001eb081990 (48621901..48623325) | Uncharacterized protein |
|  |  |  |  |  |  |  |  | Zm00001eb082000 (48718948..48722206) | Polyketide cyclase/dehydrase and lipid transport superfamily protein |
|  |  |  |  |  |  |  |  | Zm00001eb082010 (48727359..48727970) | Helitron helicase-like domain-containing protein |
|  |  |  |  |  |  |  |  | Zm00001eb082030 (48797636..48799444) | Cytochrome P450 89A2 |
|  |  |  |  |  |  |  |  | Zm00001eb082040 (48923717..48925293) | Protein BEARSKIN2 |
|  |  |  |  |  |  |  |  | Zm00001eb082050 (48964493..48967723) | NADH:flavin oxidoreductase/NADH oxidase N-terminal domain-containing protein |
|  |  |  |  |  |  |  |  | Zm00001eb082060 (48987250..48990338) | Ras-related protein RIC1 |
|  |  |  |  |  |  |  |  | Zm00001eb082070 (48990557..48993738) | GST N-terminal domain-containing protein; Ypt homolog1 |
|  |  |  |  |  |  |  |  | Zm00001eb082080 (49035325..49040647) | Protein phosphatase methylesterase 1 |
|  |  |  |  |  |  |  |  | Zm00001eb082100 (49138609..49139611) | DUF538 family protein |
|  |  |  |  |  |  |  |  | Zm00001eb082110 (49153267..49154343) | Uncharacterized protein |
|  |  |  |  |  |  |  |  | Zm00001eb082120 (49158393..49158743) | Uncharacterized protein |
|  |  |  |  |  |  |  |  | Zm00001eb082130 (49244431..49248089) | Two-component response regulator ARR16 |
| PHK76 | IAH4.2021 | 3 | 194758451 | 194884678 | 194883814 | 5.239972806 | 4 |  |  |
|  |  |  |  |  |  |  |  | Zm00001eb151140 (194758159..194759901) | Phytocyanin domain-containing protein |
|  |  |  |  |  |  |  |  | Zm00001eb151150 (194836245..194840589) | Disease resistance gene analog PIC21 |
|  |  |  |  |  |  |  |  | Zm00001eb151160 (194841782..194842840) | NTF2 domain-containing protein |
|  |  |  |  |  |  |  |  | Zm00001eb151170 (194882743..194884968) | Methylesterase 3 |
| PHK76 | IAH4.2021 | 8 | 155373364 | 156276242 | 155749799 | 6.035976377 | 20 |  |  |
|  |  |  |  |  |  |  |  | Zm00001eb360540 (155434900..155440191) | Zinc transporter 5 |
|  |  |  |  |  |  |  |  | Zm00001eb360530 (155411484..155415250) | Protein binding protein |
|  |  |  |  |  |  |  |  | Zm00001eb360550 (155530469..155532662) | Uncharacterized protein |
|  |  |  |  |  |  |  |  | Zm00001eb360560 (155579869..155583985) | Serine aminopeptidase S33 domain-containing protein; Alpha/beta-Hydrolases superfamily protein |
|  |  |  |  |  |  |  |  | Zm00001eb360570 (155587232..155588729) | Uncharacterized protein; DUF1639 family protein |
|  |  |  |  |  |  |  |  | Zm00001eb360580 (155669960..155683399) | Zinc finger protein; Putative zinc finger protein |
|  |  |  |  |  |  |  |  | Zm00001eb360590 (155735844..155750215) | Uncharacterized protein |
|  |  |  |  |  |  |  |  | Zm00001eb360610 (155836951..155841244) | Serine aminopeptidase S33 domain-containing protein; Alpha/beta-Hydrolases superfamily protein |
|  |  |  |  |  |  |  |  | Zm00001eb360620 (155840954..155854675) | Protein kinase domain-containing protein |
|  |  |  |  |  |  |  |  | Zm00001eb360630 (155975257..155983874) | Protein kinase domain-containing protein |
|  |  |  |  |  |  |  |  | Zm00001eb360640 (156052116..156063408) | Helminthosporium turcicum resistanceN1; Protein kinase domain-containing protein |
|  |  |  |  |  |  |  |  | Zm00001eb360690 (156195694..156204014) | Serine/threonine-protein phosphatase |
|  |  |  |  |  |  |  |  | Zm00001eb360710 (156246281..156250891) | RING-type E3 ubiquitin transferase |
|  |  |  |  |  |  |  |  | Zm00001eb360720 (156274743..156275188) | Uncharacterized protein |
|  |  |  |  |  |  |  |  | Zm00001eb360730 (156275300..156276876) | Hydroxyproline-rich glycoprotein family protein |
|  |  |  |  |  |  |  |  | Zm00001eb360700 (156207974..156208861) | Uncharacterized protein |
|  |  |  |  |  |  |  |  | Zm00001eb360660 (156076121..156076892) | Bifunctional inhibitor/plant lipid transfer protein/seed storage helical domain-containing protein |
|  |  |  |  |  |  |  |  | Zm00001eb360680 (156185904..156186390) | Uncharacterized protein |
|  |  |  |  |  |  |  |  | Zm00001eb360670 (156077079..156079748) | Reticulon-like protein |
|  |  |  |  |  |  |  |  | Zm00001eb360650 (156076120..156091006) | Cytidyltransferase isoform 1 |
| PHK76 | ILH1.2021 | 2 | 34398932 | 34398932 | 34398932 | 5.204872098 | 1 |  |  |
|  |  |  |  |  |  |  |  | Zm00001eb078530 (34390694..34400773) | CBF1 interacting corepressor; CBF1-interacting co-repressor CIR N-terminal domain-containing protein |
| PHK76 | ILH1.2021 | 3 | 203759073 | 204071285 | 203944217 | 5.314662738 | 3 |  |  |
|  |  |  |  |  |  |  |  | Zm00001eb153500 (203931338..203944340) | Casein kinase substrate phosphoprotein PP28 domain-containing protein; Heat-and acid-stable phosphoprotein |
|  |  |  |  |  |  |  |  | Zm00001eb153510 (203969930..203972076) | Uncharacterized protein; Regulator of chromosome condensation (RCC1) family protein |
|  |  |  |  |  |  |  |  | Zm00001eb153520 (204065538..204072197) | Transcription factor VOZ1 |
| PHK76 | INH1.2020 | 2 | 38584591 | 38585812 | 38584591 | 5.090365208 | 1 |  |  |
|  |  |  |  |  |  |  |  | Zm00001eb079430 (38583656..38599214) | galactinol--sucrose galactosyltransferase |
| PHK76 | INH1.2020 | 3 | 201064486 | 201065907 | 201065626 | 5.112182819 | 1 |  |  |
|  |  |  |  |  |  |  |  | Zm00001eb152670 (201063268..201066105) | Interactor of constitutive active ROPs 1; Interactor of constitutive active ROPs 4 |
| PHK76 | NCH1.2021 | 2 | 46179971 | 46183987 | 46183776 | 5.286041725 | 1 |  |  |
|  |  |  |  |  |  |  |  | Zm00001eb081420 (46179764..46184123) | Steroid nuclear receptor ligand-binding |
| PHK76 | NCH1.2021 | 3 | 3509477 | 3510643 | 3510643 | 5.22699144 | 1 |  |  |
|  |  |  |  |  |  |  |  | Zm00001eb119980 (3505517..3511045) | DUF455 family protein |
| PHK76 | NCH1.2021 | 7 | 145323542 | 145337494 | 145334459 | 5.588255117 | 2 |  |  |
|  |  |  |  |  |  |  |  | Zm00001eb318390 (145323466..145331935) | Bromo domain-containing protein; Bromodomain protein 103 |
|  |  |  |  |  |  |  |  | Zm00001eb318400 (145332449..145338302) | Proteasome subunit beta |
| PHK76 | NCH1.2021 | 8 | 155670876 | 155749799 | 155683367 | 5.008453492 | 2 |  |  |
|  |  |  |  |  |  |  |  | Zm00001eb360580 (155669960..155683399) | Zinc finger protein; Putative zinc finger protein |
|  |  |  |  |  |  |  |  | Zm00001eb360590 (155735844..155750215) | Uncharacterized protein |
| PHK76 | WIH2.2020 | 2 | 80518641 | 80629174 | 80618534 | 5.156033481 | 2 |  |  |
|  |  |  |  |  |  |  |  | Zm00001eb086170 (80517380..80519503) | Pentatricopeptide repeat-containing protein |
|  |  |  |  |  |  |  |  | Zm00001eb086180 (80613263..80629344) | Protein kinase domain-containing protein |
| PHK76 | WIH2.2020 | 6 | 162624311 | 162636693 | 162636561 | 5.205945806 | 3 |  |  |
|  |  |  |  |  |  |  |  | Zm00001eb289550 (162620435..162624333) | uracil phosphoribosyltransferase |
|  |  |  |  |  |  |  |  | Zm00001eb289560 (162627928..162633827) | NADP-dependent oxidoreductase domain-containing protein |
|  |  |  |  |  |  |  |  | Zm00001eb289570 (162631979..162637087) | Aldo-keto reductase family 4 member C9 (Aldose reductase); NADP-dependent oxidoreductase domain-containing protein |
| PHK76 | WIH2.2021 | 2 | 33487453 | 33489553 | 33488357 | 5.061509083 | 1 |  |  |
|  |  |  |  |  |  |  |  | Zm00001eb078290 (33487363..33489618) | Cytochrome P450 71A26 |
| PHK76 | WIH2.2021 | 3 | 197124049 | 197326584 | 197206028 | 5.086650136 | 3 |  |  |
|  |  |  |  |  |  |  |  | Zm00001eb151770 (197205724..197216363) | Dymeclin |
|  |  |  |  |  |  |  |  | Zm00001eb151780 (197316065..197318128) | Homocysteine S-methyltransferase4 |
|  |  |  |  |  |  |  |  | Zm00001eb151790 (197317599..197330106) | Signal recognition particle 54 kDa protein |
| PHP02 | DEH1.2020 | 2 | 166185448 | 166904164 | 166904164 | 5.516739752 | 7 |  |  |
|  |  |  |  |  |  |  |  | Zm00001eb095470 (166184962..166185931) | Peptidyl-prolyl cis-trans isomerase |
|  |  |  |  |  |  |  |  | Zm00001eb095480 (166273248..166288490) | Protein NEDD1; Anaphase-promoting complex subunit 4 WD40 domain-containing protein |
|  |  |  |  |  |  |  |  | Zm00001eb095500 (166373925..166383826) | Tyrosine-protein phosphatase; Putative tyrosine-protein phosphatase |
|  |  |  |  |  |  |  |  | Zm00001eb095510 (166516933..166522696) | Protein kinase domain-containing protein; Serine/threonine protein kinase 3 |
|  |  |  |  |  |  |  |  | Zm00001eb095520 (166549067..166549690) | Uncharacterized protein |
|  |  |  |  |  |  |  |  | Zm00001eb095530 (166658652..166664196) | ARM repeat superfamily protein |
|  |  |  |  |  |  |  |  | Zm00001eb095540 (166900507..166906461) | Polyadenylate-binding protein (PABP) |
| PHP02 | DEH1.2020 | 7 | 131727168 | 131738538 | 131738127 | 5.372407165 | 1 |  |  |
|  |  |  |  |  |  |  |  | Zm00001eb315020 (131727150..131739268) | ARP protein (REF); Alcohol dehydrogenase-like N-terminal domain-containing protein |
| PHP02 | DEH1.2020 | 9 | 18962185 | 19346616 | 19309895 | 5.57118468 | 12 |  |  |
|  |  |  |  |  |  |  |  | Zm00001eb376020 (18962006..18968292) | Acyl-coenzyme A oxidase |
|  |  |  |  |  |  |  |  | Zm00001eb376030 (19050656..19057886) | DUF223 domain-containing protein |
|  |  |  |  |  |  |  |  | Zm00001eb376040 (19060329..19062254) | Uncharacterized protein |
|  |  |  |  |  |  |  |  | Zm00001eb376050 (19060547..19061214) | Uncharacterized protein |
|  |  |  |  |  |  |  |  | Zm00001eb376060 (19117164..19117767) | DUF569 domain-containing protein |
|  |  |  |  |  |  |  |  | Zm00001eb376070 (19151614..19154301) | DUF569 domain-containing protein |
|  |  |  |  |  |  |  |  | Zm00001eb376080 (19183076..19186226) | MBD domain-containing protein |
|  |  |  |  |  |  |  |  | Zm00001eb376090 (19258409..19259239) | DUF569 domain-containing protein |
|  |  |  |  |  |  |  |  | Zm00001eb376100 (19309352..19320805) | "Starch synthase, chloroplastic/amyloplastic" |
|  |  |  |  |  |  |  |  | Zm00001eb376110 (19324629..19325718) | AP2-EREBP transcription factor |
|  |  |  |  |  |  |  |  | Zm00001eb376130 (19343656..19347607) | GDSL esterase/lipase |
|  |  |  |  |  |  |  |  | Zm00001eb376120 (19339094..19343364) | Ubiquitin-associated/translation elongation factor EF1B protein |
| PHP02 | DEH1.2021 | 1 | 298363375 | 298423914 | 298408930 | 5.124811396 | 2 |  |  |
|  |  |  |  |  |  |  |  | Zm00001eb061990 (298358526..298365042) | Uncharacterized protein |
|  |  |  |  |  |  |  |  | Zm00001eb062000 (298408180..298424189) | RNA-binding protein 8A |
| PHP02 | DEH1.2021 | 2 | 191652493 | 192627225 | 191757086 | 6.277274561 | 25 |  |  |
|  |  |  |  |  |  |  |  | Zm00001eb100880 (191744743..191745746) | Cyclin-dependent kinase inhibitor domain-containing protein |
|  |  |  |  |  |  |  |  | Zm00001eb100860 (191651871..191653018) | Uncharacterized protein |
|  |  |  |  |  |  |  |  | Zm00001eb100900 (191756947..191758451) | Uncharacterized protein |
|  |  |  |  |  |  |  |  | Zm00001eb100890 (191748719..191756398) | Uncharacterized protein |
|  |  |  |  |  |  |  |  | Zm00001eb100920 (191790294..191814082) | Protein transport protein SEC23 |
|  |  |  |  |  |  |  |  | Zm00001eb100910 (191786638..191787502) | Beta-glucuronosyltransferase GlcAT14B |
|  |  |  |  |  |  |  |  | Zm00001eb100930 (191909253..191910908) | Putative carboxylesterase 2 |
|  |  |  |  |  |  |  |  | Zm00001eb100950 (191981881..191983417) | Gibberellin receptor GID1L2 |
|  |  |  |  |  |  |  |  | Zm00001eb100970 (192012849..192015174) | Patatin |
|  |  |  |  |  |  |  |  | Zm00001eb100940 (191938768..191941240) | Leucine-rich repeat-containing N-terminal plant-type domain-containing protein |
|  |  |  |  |  |  |  |  | Zm00001eb100960 (191983790..191985001) | Putative carboxylesterase 2 |
|  |  |  |  |  |  |  |  | Zm00001eb100980 (192064781..192069688) | Polyadenylate-binding protein RBP47B |
|  |  |  |  |  |  |  |  | Zm00001eb100990 (192070058..192071081) | Serine carboxypeptidase-like 34 |
|  |  |  |  |  |  |  |  | Zm00001eb101000 (192082886..192086044) | Xylose isomerase |
|  |  |  |  |  |  |  |  | Zm00001eb101010 (192221331..192225444) | Ternary complex factor MIP1-like protein; Ternary complex factor MIP1 leucine-zipper domain-containing protein |
|  |  |  |  |  |  |  |  | Zm00001eb101030 (192263171..192263740) | Protein MIZU-KUSSEI 1 |
|  |  |  |  |  |  |  |  | Zm00001eb101040 (192294715..192295919) | BHLH transcription factor (Transcription factor bHLH51) |
|  |  |  |  |  |  |  |  | Zm00001eb101050 (192295801..192300264) | Carbonic anhydrase |
|  |  |  |  |  |  |  |  | Zm00001eb101060 (192453288..192455426) | AT-hook motif nuclear-localized protein 17 |
|  |  |  |  |  |  |  |  | Zm00001eb101070 (192453576..192455426) | Uncharacterized protein |
|  |  |  |  |  |  |  |  | Zm00001eb101080 (192522376..192524875) | MADS box interactor-like |
|  |  |  |  |  |  |  |  | Zm00001eb101100 (192534335..192541992) | Threonine endopeptidase |
|  |  |  |  |  |  |  |  | Zm00001eb101090 (192524874..192530968) | Glucose-6-phosphate isomerase |
|  |  |  |  |  |  |  |  | Zm00001eb101110 (192544005..192544508) | Fe2OG dioxygenase domain-containing protein |
|  |  |  |  |  |  |  |  | Zm00001eb101120 (192624947..192627498) | D-type cyclin; Uncharacterized protein |
| PHP02 | DEH1.2021 | 7 | 147774487 | 147887468 | 147774487 | 5.284828195 | 8 |  |  |
|  |  |  |  |  |  |  |  | Zm00001eb319020 (147779934..147780802) | YY1 protein |
|  |  |  |  |  |  |  |  | Zm00001eb319010 (147773749..147778434) | RING-type domain-containing protein; Protein binding protein |
|  |  |  |  |  |  |  |  | Zm00001eb319030 (147780699..147790384) | UBX domain-containing protein; Plant UBX domain-containing protein 8 |
|  |  |  |  |  |  |  |  | Zm00001eb319040 (147791106..147792420) | "Glucan endo-1, 3-beta-D-glucosidase" |
|  |  |  |  |  |  |  |  | Zm00001eb319060 (147842854..147845360) | Protein ROOT PRIMORDIUM DEFECTIVE 1 |
|  |  |  |  |  |  |  |  | Zm00001eb319050 (147830796..147833634) | RHOMBOID-like protein |
|  |  |  |  |  |  |  |  | Zm00001eb319080 (147874032..147874803) |  |
|  |  |  |  |  |  |  |  | Zm00001eb319090 (147881484..147888019) | Homeodomain leucine zipper family IV protein |
| PHP02 | IAH2.2021 | 2 | 149287679 | 149287679 | 149287679 | 5.030326629 | 1 |  |  |
|  |  |  |  |  |  |  |  | Zm00001eb092900 (149287661..149291098) | Protein TRAUCO |
| PHP02 | IAH2.2021 | 7 | 123208440 | 123209943 | 123209943 | 5.139703639 | 2 |  |  |
|  |  |  |  |  |  |  |  | Zm00001eb313400 (123207787..123209204) | adenosylmethionine decarboxylase |
|  |  |  |  |  |  |  |  | Zm00001eb313410 (123209046..123210160) | S-adenosylmethionine decarboxylase proenzyme |
| PHP02 | IAH3.2021 | 2 | 135410575 | 136644262 | 136643795 | 5.204755716 | 9 |  |  |
|  |  |  |  |  |  |  |  | Zm00001eb091310 (135426444..135427709) | protein-serine/threonine phosphatase |
|  |  |  |  |  |  |  |  | Zm00001eb091300 (135410126..135411588) | Uncharacterized protein |
|  |  |  |  |  |  |  |  | Zm00001eb091340 (136049559..136051605) | Arf-GAP domain-containing protein |
|  |  |  |  |  |  |  |  | Zm00001eb091350 (136133490..136140926) | Protein-tyrosine-phosphatase PTP1; Tyrosine-protein phosphatase domain-containing protein |
|  |  |  |  |  |  |  |  | Zm00001eb091360 (136158418..136159413) | Dirigent protein |
|  |  |  |  |  |  |  |  | Zm00001eb091370 (136314889..136315804) | Dirigent protein |
|  |  |  |  |  |  |  |  | Zm00001eb091380 (136389508..136391354) | Uncharacterized protein |
|  |  |  |  |  |  |  |  | Zm00001eb091390 (136402804..136420634) | Uncharacterized protein |
|  |  |  |  |  |  |  |  | Zm00001eb091400 (136643526..136644455) | Dirigent protein |
| PHP02 | IAH3.2021 | 7 | 137403846 | 137420875 | 137413763 | 5.018496287 | 2 |  |  |
|  |  |  |  |  |  |  |  | Zm00001eb316440 (137403596..137410874) | Putative BPI/LBP family protein |
|  |  |  |  |  |  |  |  | Zm00001eb316450 (137410930..137421089) | DUF1664 domain-containing protein |
| PHP02 | IAH4.2021 | 2 | 149328637 | 149455026 | 149328637 | 5.23733144 | 3 |  |  |
|  |  |  |  |  |  |  |  | Zm00001eb092910 (149328552..149345553) | Strictosidine synthase conserved region domain-containing protein |
|  |  |  |  |  |  |  |  | Zm00001eb092920 (149345763..149346933) | Uncharacterized protein |
|  |  |  |  |  |  |  |  | Zm00001eb092940 (149454841..149455197) | Uncharacterized protein |
| PHP02 | IAH4.2021 | 7 | 147716439 | 148025225 | 147732943 | 6.119390063 | 21 |  |  |
|  |  |  |  |  |  |  |  | Zm00001eb318990 (147715244..147717880) | Ubiquitin-like domain-containing protein; BAG family molecular chaperone regulator 1; Protein binding protein |
|  |  |  |  |  |  |  |  | Zm00001eb319000 (147731360..147733872) | F-box protein PP2-A13 |
|  |  |  |  |  |  |  |  | Zm00001eb319020 (147779934..147780802) | YY1 protein |
|  |  |  |  |  |  |  |  | Zm00001eb319030 (147780699..147790384) | UBX domain-containing protein; Plant UBX domain-containing protein 8 |
|  |  |  |  |  |  |  |  | Zm00001eb319010 (147773749..147778434) | RING-type domain-containing protein; Protein binding protein (RING/U-box superfamily protein) |
|  |  |  |  |  |  |  |  | Zm00001eb319040 (147791106..147792420) | "Glucan endo-1, 3-beta-D-glucosidase" |
|  |  |  |  |  |  |  |  | Zm00001eb319060 (147842854..147845360) | Protein ROOT PRIMORDIUM DEFECTIVE 1 |
|  |  |  |  |  |  |  |  | Zm00001eb319050 (147830796..147833634) | RHOMBOID-like protein |
|  |  |  |  |  |  |  |  | Zm00001eb319080 (147874032..147874803) |  |
|  |  |  |  |  |  |  |  | Zm00001eb319090 (147881484..147888019) | Homeodomain leucine zipper family IV protein |
|  |  |  |  |  |  |  |  | Zm00001eb319100 (147889758..147891284) | SAUR-like auxin-responsive protein family |
|  |  |  |  |  |  |  |  | Zm00001eb319110 (147945930..147951379) | Ypt/Rab-GAP domain of gyp1p superfamily protein |
|  |  |  |  |  |  |  |  | Zm00001eb319130 (147957385..147963651) | Protein TILLER ANGLE CONTROL 1 |
|  |  |  |  |  |  |  |  | Zm00001eb319120 (147954681..147956941) | Uncharacterized protein; MnmG N-terminal domain-containing protein |
|  |  |  |  |  |  |  |  | Zm00001eb319140 (147973341..147975292) | Uncharacterized protein |
|  |  |  |  |  |  |  |  | Zm00001eb319150 (147976312..147980256) | DUF295 domain-containing protein |
|  |  |  |  |  |  |  |  | Zm00001eb319170 (148001961..148003234) | ALA-interacting subunit |
|  |  |  |  |  |  |  |  | Zm00001eb319180 (148008660..148009182) |  |
|  |  |  |  |  |  |  |  | Zm00001eb319210 (148019596..148023694) | Rhodanese domain-containing protein |
|  |  |  |  |  |  |  |  | Zm00001eb319220 (148023190..148026200) | Ras-related protein RABA3 |
|  |  |  |  |  |  |  |  | Zm00001eb319160 (148000313..148000849) | Glutaredoxin domain-containing protein |
| PHP02 | IAH4.2021 | 8 | 13739741 | 13771292 | 13769510 | 5.248848885 | 3 |  |  |
|  |  |  |  |  |  |  |  | Zm00001eb335570 (13735643..13739744) | Mandelate racemase/muconate lactonizing enzyme C-terminal domain-containing protein; Dipeptide epimerase |
|  |  |  |  |  |  |  |  | Zm00001eb335580 (13753550..13753868) | MYB-CC type transcription factor LHEQLE-containing domain-containing protein |
|  |  |  |  |  |  |  |  | Zm00001eb335590 (13768096..13771513) | Octicosapeptide/Phox/Bem1p family protein |
| PHP02 | MIH1.2021 | 3 | 181097729 | 181098373 | 181097729 | 5.135141435 | 1 |  |  |
|  |  |  |  |  |  |  |  | Zm00001eb146920 (181097458..181098558) | Protein YLS9 |
| PHP02 | MIH1.2021 | 9 | 18538860 | 22319190 | 21340633 | 6.999557135 | 134 |  |  |
|  |  |  |  |  |  |  |  | Zm00001eb+I387:I520375820 (18538642..18544276) | Serine/threonine-protein kinase; RING-type E3 ubiquitin transferase |
|  |  |  |  |  |  |  |  | Zm00001eb375840 (18647682..18657775) | 2-oxoglutarate (2OG) and Fe(II)-dependent oxygenase superfamily protein |
|  |  |  |  |  |  |  |  | Zm00001eb375920 (18716972..18717298) | Transcription elongation factor 1 homolog |
|  |  |  |  |  |  |  |  | Zm00001eb375930 (18724184..18724513) | Transcription elongation factor 1 homolog |
|  |  |  |  |  |  |  |  | Zm00001eb375940 (18727407..18727730) | Transcription elongation factor 1 homolog |
|  |  |  |  |  |  |  |  | Zm00001eb375830 (18635800..18643092) | Developmental protein SEPALLATA 2 |
|  |  |  |  |  |  |  |  | Zm00001eb375850 (18650181..18658040) | Uncharacterized protein |
|  |  |  |  |  |  |  |  | Zm00001eb375870 (18662305..18662955) | High-affinity nitrate transporter 2.4 |
|  |  |  |  |  |  |  |  | Zm00001eb375880 (18663087..18664270) | Uncharacterized protein |
|  |  |  |  |  |  |  |  | Zm00001eb375890 (18684444..18684770) | Transcription elongation factor 1 homolog |
|  |  |  |  |  |  |  |  | Zm00001eb375900 (18700899..18712273) | Uncharacterized protein |
|  |  |  |  |  |  |  |  | Zm00001eb375950 (18732013..18740826) | Transcription elongation factor 1 homolog |
|  |  |  |  |  |  |  |  | Zm00001eb375960 (18754617..18756705) | DUF569 domain-containing protein |
|  |  |  |  |  |  |  |  | Zm00001eb375980 (18860750..18861235) | Uncharacterized protein |
|  |  |  |  |  |  |  |  | Zm00001eb376000 (18868075..18868560) |  |
|  |  |  |  |  |  |  |  | Zm00001eb376010 (18886725..18888714) | DUF569 domain-containing protein |
|  |  |  |  |  |  |  |  | Zm00001eb376020 (18962006..18968292) | Acyl-coenzyme A oxidase |
|  |  |  |  |  |  |  |  | Zm00001eb376040 (19060329..19062254) | Uncharacterized protein |
|  |  |  |  |  |  |  |  | Zm00001eb376030 (19050656..19057886) | DUF223 domain-containing protein |
|  |  |  |  |  |  |  |  | Zm00001eb376050 (19060547..19061214) | Uncharacterized protein |
|  |  |  |  |  |  |  |  | Zm00001eb376060 (19117164..19117767) | DUF569 domain-containing protein |
|  |  |  |  |  |  |  |  | Zm00001eb376070 (19151614..19154301) | DUF569 domain-containing protein |
|  |  |  |  |  |  |  |  | Zm00001eb376080 (19183076..19186226) | MBD domain-containing protein |
|  |  |  |  |  |  |  |  | Zm00001eb376090 (19258409..19259239) | DUF569 domain-containing protein |
|  |  |  |  |  |  |  |  | Zm00001eb376100 (19309352..19320805) | "Starch synthase, chloroplastic/amyloplastic" |
|  |  |  |  |  |  |  |  | Zm00001eb376110 (19324629..19325718) | AP2-EREBP transcription factor |
|  |  |  |  |  |  |  |  | Zm00001eb376120 (19339094..19343364) | Ubiquitin-associated/translation elongation factor EF1B protein |
|  |  |  |  |  |  |  |  | Zm00001eb376130 (19343656..19347607) | GDSL esterase/lipase |
|  |  |  |  |  |  |  |  | Zm00001eb376140 (19403188..19404013) | Uncharacterized protein |
|  |  |  |  |  |  |  |  | Zm00001eb376150 (19403190..19403985) | gene |
|  |  |  |  |  |  |  |  | Zm00001eb376160 (19419057..19431789) | Multidrug resistance-associated protein3 |
|  |  |  |  |  |  |  |  | Zm00001eb376180 (19432377..19433847) | Atpob1 |
|  |  |  |  |  |  |  |  | Zm00001eb376210 19508598..19509641) | DUF295 domain-containing protein |
|  |  |  |  |  |  |  |  | Zm00001eb376190 (19502510..19504631) | Glyoxalase/bleomycin resistance protein/dioxygenase |
|  |  |  |  |  |  |  |  | Zm00001eb376220 (19514580..19518554) | Lactoylglutathione lyase / glyoxalase I family protein |
|  |  |  |  |  |  |  |  | Zm00001eb376240 (19524099..19525142) | DUF295 domain-containing protein |
|  |  |  |  |  |  |  |  | Zm00001eb376250 (19530082..19545139) | Lactoylglutathione lyase / glyoxalase I family protein |
|  |  |  |  |  |  |  |  | Zm00001eb376200 (19504839..19507731) | rRNA-processing protein FCF1 |
|  |  |  |  |  |  |  |  | Zm00001eb376230 (19519335..19522940) | PIN domain-containing protein |
|  |  |  |  |  |  |  |  | Zm00001eb376260 (19581035..19583156) | Lactoylglutathione lyase / glyoxalase I family protein |
|  |  |  |  |  |  |  |  | Zm00001eb376280 (19587088..19588131) | DUF295 domain-containing protein |
|  |  |  |  |  |  |  |  | Zm00001eb376310 (19625405..19627600) |  |
|  |  |  |  |  |  |  |  | Zm00001eb376320 (19634308..19636503) | Lactoylglutathione lyase / glyoxalase I family protein |
|  |  |  |  |  |  |  |  | Zm00001eb376340 (19643170..19644213) | DUF295 domain-containing protein |
|  |  |  |  |  |  |  |  | Zm00001eb376350 (19648672..19651345) | Lactoylglutathione lyase / glyoxalase I family protein |
|  |  |  |  |  |  |  |  | Zm00001eb376360 (19656684..19657727) | Uncharacterized protein |
|  |  |  |  |  |  |  |  | Zm00001eb376370 (19661825..19662589) | Uncharacterized protein |
|  |  |  |  |  |  |  |  | Zm00001eb376380 (19663131..19664393) | ABC transmembrane type-1 domain-containing protein |
|  |  |  |  |  |  |  |  | Zm00001eb376300 (19602716..19606322) | PIN domain-containing protein |
|  |  |  |  |  |  |  |  | Zm00001eb376330 (19638320..19655478) | Nucleolar protein involved in pre-mRNA processing |
|  |  |  |  |  |  |  |  | Zm00001eb376390 (19833628..19836367) | Splicing factor 45 |
|  |  |  |  |  |  |  |  | Zm00001eb376400 (19838473..19842846) | Putative WRKY DNA-binding domain superfamily protein |
|  |  |  |  |  |  |  |  | Zm00001eb376410 (19879342..19884048) | U-box domain-containing protein |
|  |  |  |  |  |  |  |  | Zm00001eb376420 (19889571..19890140) |  |
|  |  |  |  |  |  |  |  | Zm00001eb376440 (19917446..19918261) | Uncharacterized protein |
|  |  |  |  |  |  |  |  | Zm00001eb376450 (19947959..19948619) | Gcp-like domain-containing protein |
|  |  |  |  |  |  |  |  | Zm00001eb376460 (19949951..19950272) | Pentatricopeptide repeat-containing protein |
|  |  |  |  |  |  |  |  | Zm00001eb376470 (19978278..19979384) | Aspartic proteinase A1 |
|  |  |  |  |  |  |  |  | Zm00001eb376480 (19989105..19996549) | Protein-tyrosine sulfotransferase |
|  |  |  |  |  |  |  |  | Zm00001eb376490 (20057118..20064158) | Long-chain-fatty-acid--CoA ligase |
|  |  |  |  |  |  |  |  | Zm00001eb376510 (20068328..20069154) | "Succinate dehydrogenase assembly factor 4, mitochondrial" |
|  |  |  |  |  |  |  |  | Zm00001eb376530 (20079355..20081807) | GDSL esterase/lipase; Lipase-like |
|  |  |  |  |  |  |  |  | Zm00001eb376500 (20061343..20062056) | Uncharacterized protein |
|  |  |  |  |  |  |  |  | Zm00001eb376520 (20071705..20078877) | Hexosyltransferase |
|  |  |  |  |  |  |  |  | Zm00001eb376540 (20240498..20240961) | VQ domain-containing protein |
|  |  |  |  |  |  |  |  | Zm00001eb376560 (20304950..20305413) |  |
|  |  |  |  |  |  |  |  | Zm00001eb376600 (20445936..20450128) | Calmodulin binding protein |
|  |  |  |  |  |  |  |  | Zm00001eb376570 (20432209..20434996) | Zea nodulation homolog1; Alpha-L-fucosidase 2 |
|  |  |  |  |  |  |  |  | Zm00001eb376580 (20442230..20443713) | Uncharacterized protein |
|  |  |  |  |  |  |  |  | Zm00001eb376590 (20444149..20446107) | HXXXD-type acyl-transferase family protein |
|  |  |  |  |  |  |  |  | Zm00001eb376620 (20566416..20566719) | Uncharacterized protein |
|  |  |  |  |  |  |  |  | Zm00001eb376630 (20581735..20585861) | GW2 |
|  |  |  |  |  |  |  |  | Zm00001eb376640 (20685730..20691972) | Mitogen-activated protein kinase |
|  |  |  |  |  |  |  |  | Zm00001eb376650 (20692141..20695779) | DUF676 domain-containing protein; Hydrolase-like protein family |
|  |  |  |  |  |  |  |  | Zm00001eb376660 (20701198..20704533) | F-box protein MAX2 |
|  |  |  |  |  |  |  |  | Zm00001eb376670 (20704522..20712771) | Proteasome subunit beta |
|  |  |  |  |  |  |  |  | Zm00001eb376680 (20713479..20728917) | Putative sugar phosphate/phosphate translocator |
|  |  |  |  |  |  |  |  | Zm00001eb376700 (20765539..20770665) | Pentatricopeptide repeat-containing protein |
|  |  |  |  |  |  |  |  | Zm00001eb376690 (20765487..20766505) | Glycosyl transferase CAP10 domain-containing protein |
|  |  |  |  |  |  |  |  | Zm00001eb376710 (20829686..20831292) | Dehydrin family protein expressed |
|  |  |  |  |  |  |  |  | Zm00001eb376720 (20831910..20837110) | "Ubiquinone biosynthesis O-methyltransferase, mitochondria |
|  |  |  |  |  |  |  |  | Zm00001eb376730 (20839309..20845781) | Profilin homolog5; Orphans transcription factor (Profilin homolog5); Uncharacterized protein |
|  |  |  |  |  |  |  |  | Zm00001eb376740 (20846686..20850490) | Profilin-5 (ZmPRO5) (Double B-box zinc finger protein 11) (Pollen allergen Zea m 12) (allergen Zea m 12) |
|  |  |  |  |  |  |  |  | Zm00001eb376750 (20853035..20861375) | Dual specificity protein phosphatase DSP8; Tyrosine specific protein phosphatases domain-containing protein |
|  |  |  |  |  |  |  |  | Zm00001eb376780 (20963923..20969251) | ATP-dependent 6-phosphofructokinase |
|  |  |  |  |  |  |  |  | Zm00001eb376790 (20969937..20973027) | Tryptophan synthase beta chain-like PALP domain-containing protein; Cysteine synthase |
|  |  |  |  |  |  |  |  | Zm00001eb376800 (20983342..20984308) | Uncharacterized protein |
|  |  |  |  |  |  |  |  | Zm00001eb376820 (21032581..21033030) | Uncharacterized protein |
|  |  |  |  |  |  |  |  | Zm00001eb376840 (21039872..21045940) | Uncharacterized protein; NB-ARC domain-containing protein |
|  |  |  |  |  |  |  |  | Zm00001eb376850 (21048095..21054027) | Saposin B-type domain-containing protein |
|  |  |  |  |  |  |  |  | Zm00001eb376870 (21098480..21099308) | Ubiquitin-like domain-containing protein |
|  |  |  |  |  |  |  |  | Zm00001eb376880 (21099772..21102568) | Trafficking protein particle complex subunit |
|  |  |  |  |  |  |  |  | Zm00001eb376860 (21095830..21097630) | HXXXD-type acyl-transferase family protein |
|  |  |  |  |  |  |  |  | Zm00001eb376890 (21119954..21121937) | Tryptophan synthase beta chain-like PALP domain-containing protein; Pyridoxal-5'-phosphate-dependent enzyme family protein |
|  |  |  |  |  |  |  |  | Zm00001eb376900 (21124514..21127951) | Tryptophan synthase beta chain-like PALP domain-containing protein |
|  |  |  |  |  |  |  |  | Zm00001eb376910 (21127836..21130893) | Lactoylglutathione lyase / glyoxalase I family protein |
|  |  |  |  |  |  |  |  | Zm00001eb376970 (21188308..21192571) |  |
|  |  |  |  |  |  |  |  | Zm00001eb376930 (21156037..21168630) | Pyridoxal-5'-phosphate-dependent enzyme family protein |
|  |  |  |  |  |  |  |  | Zm00001eb376980 (21194827..21198952) | Nucleosome assembly protein 1 |
|  |  |  |  |  |  |  |  | Zm00001eb376960 (21186644..21187687) | DUF295 domain-containing protein |
|  |  |  |  |  |  |  |  | Zm00001eb376920 (21154373..21155416) | DUF295 domain-containing protein |
|  |  |  |  |  |  |  |  | Zm00001eb376940 (21158231..21160894) |  |
|  |  |  |  |  |  |  |  | Zm00001eb376950 (21165349..21166392) | DUF295 domain-containing protein |
|  |  |  |  |  |  |  |  | Zm00001eb376990 (21320425..21338542) | Uncharacterized protein |
|  |  |  |  |  |  |  |  | Zm00001eb377000 (21339736..21342929) | CDT1-like protein a chloroplastic |
|  |  |  |  |  |  |  |  | Zm00001eb377010 (21344890..21345865) | Uncharacterized protein |
|  |  |  |  |  |  |  |  | Zm00001eb377020 (21348604..21350111) | Uncharacterized protein |
|  |  |  |  |  |  |  |  | Zm00001eb377030 (21350408..21352445) | ABC1 atypical kinase-like domain-containing protein |
|  |  |  |  |  |  |  |  | Zm00001eb377040 (21425624..21435126) | Uncharacterized protein |
|  |  |  |  |  |  |  |  | Zm00001eb377050 (21436018..21440588) | Mitogen-activated protein kinase kinase 2 |
|  |  |  |  |  |  |  |  | Zm00001eb377060 (21455212..21455854) | Uncharacterized protein |
|  |  |  |  |  |  |  |  | Zm00001eb377090 (21601048..21601673) | Uncharacterized protein |
|  |  |  |  |  |  |  |  | Zm00001eb377100 (21708941..21710146) | Protein kinase domain containing protein |
|  |  |  |  |  |  |  |  | Zm00001eb377110 (21711593..21712754) | Uncharacterized protein |
|  |  |  |  |  |  |  |  | Zm00001eb377120 (21714835..21715778) | Uncharacterized protein |
|  |  |  |  |  |  |  |  | Zm00001eb377130 (21772461..21775833) | FeS cluster biogenesis domain-containing protein; Iron-sulfur assembly protein IscA |
|  |  |  |  |  |  |  |  | Zm00001eb377140 (21774135..21775398) | Secreted protein |
|  |  |  |  |  |  |  |  | Zm00001eb377150 (21777076..21781133) | DUF1995 domain-containing protein; DUF1995 domain protein |
|  |  |  |  |  |  |  |  | Zm00001eb377160 (21780973..21787760) | WRKY domain-containing protein |
|  |  |  |  |  |  |  |  | Zm00001eb377170 (21834848..21848622) | Helicase C-terminal domain-containing protein |
|  |  |  |  |  |  |  |  | Zm00001eb377180 (21935756..21937817) | Putative AP2/EREBP transcription factor superfamily protein |
|  |  |  |  |  |  |  |  | Zm00001eb377190 (21995093..21997068) | Uncharacterized protein |
|  |  |  |  |  |  |  |  | Zm00001eb377200 (21995137..21996747) | Pectate lyase |
|  |  |  |  |  |  |  |  | Zm00001eb377210 (22073218..22080492) | "Translation factor GUF1 homolog, mitochondrial" |
|  |  |  |  |  |  |  |  | Zm00001eb377230 (22139815..22141307) | Uncharacterized protein |
|  |  |  |  |  |  |  |  | Zm00001eb377220 (22128621..22129257) | Replication protein A OB domain-containing protein |
|  |  |  |  |  |  |  |  | Zm00001eb377240 (22141416..22143367) | Uncharacterized protein |
|  |  |  |  |  |  |  |  | Zm00001eb377250 (22148333..22156167) | Peptidase M14 carboxypeptidase A domain-containing protein |
|  |  |  |  |  |  |  |  | Zm00001eb377270 (22243520..22254128) | Coatomer subunit beta |
|  |  |  |  |  |  |  |  | Zm00001eb377260 (22180911..22186039) | Uncharacterized protein |
|  |  |  |  |  |  |  |  | Zm00001eb377280 (22280873..22287798) | DNA-repair protein XRCC1; Uncharacterized protein |
|  |  |  |  |  |  |  |  | Zm00001eb377290 (22301714..22305830) | Pre-mRNA-splicing factor 38; PRP38 family protein |
|  |  |  |  |  |  |  |  | Zm00001eb377300 (22312047..22317106) | 1-deoxy-D-xylulose-5-phosphate synthase |
|  |  |  |  |  |  |  |  | Zm00001eb377310 (22317536..22319277) | Manganese/iron superoxide dismutase C-terminal domain-containing protein |
| PHP02 | MNH1.2020 | 1 | 165030795 | 165032266 | 165032266 | 5.523466239 | 1 |  |  |
|  |  |  |  |  |  |  |  | Zm00001eb030010 (165030710..165032761) | RING-type domain-containing protein; Putative E3 ubiquitin-protein ligase RHY1A |
| PHP02 | MNH1.2020 | 2 | 166182346 | 169534370 | 167386083 | 6.277505896 | 47 |  |  |
|  |  |  |  |  |  |  |  | Zm00001eb095470 (166184962..166185931) | Peptidyl-prolyl cis-trans isomerase (PPIase) |
|  |  |  |  |  |  |  |  | Zm00001eb095460 (166182346..166182898) | Peptidase M16 C-terminal domain-containing protein |
|  |  |  |  |  |  |  |  | Zm00001eb095480 (166273248..166288490) | Protein NEDD1; Anaphase-promoting complex subunit 4 WD40 domain-containing protein |
|  |  |  |  |  |  |  |  | Zm00001eb095510 (166516933..166522696) | Protein kinase domain-containing protein; Serine/threonine protein kinase 3 |
|  |  |  |  |  |  |  |  | Zm00001eb095530 (166658652..166664196) | ARM repeat superfamily protein |
|  |  |  |  |  |  |  |  | Zm00001eb095500 (166373925..166383826) | Tyrosine-protein phosphatase; Putative tyrosine-protein phosphatase |
|  |  |  |  |  |  |  |  | Zm00001eb095520 (166549067..166549690) | Uncharacterized protein |
|  |  |  |  |  |  |  |  | Zm00001eb095540 (166900507..166906461) | Polyadenylate-binding protein (PABP) |
|  |  |  |  |  |  |  |  | Zm00001eb095550 (167021674..167022917) | Peroxidase |
|  |  |  |  |  |  |  |  | Zm00001eb095560 (167384845..167405370) | RNA binding |
|  |  |  |  |  |  |  |  | Zm00001eb095570 (167405600..167407926) | Uncharacterized protein |
|  |  |  |  |  |  |  |  | Zm00001eb095580 (167425436..167426143) | F8M12.18 protein |
|  |  |  |  |  |  |  |  | Zm00001eb095600 (167468425..167469407) | Putative GEM-like protein 8 |
|  |  |  |  |  |  |  |  | Zm00001eb095610 (167473023..167473567) | Uncharacterized protein |
|  |  |  |  |  |  |  |  | Zm00001eb095620 (167752814..167755257) | yclin11; Cyclin-like domain-containing protein; Cyclin N-terminal domain-containing protein |
|  |  |  |  |  |  |  |  | Zm00001eb095630 (167884100..167892257) | DNA-directed RNA polymerase RpoA/D/Rpb3-type domain-containing protein; DNA-directed RNA polymerase II 36 kDa polypeptide A |
|  |  |  |  |  |  |  |  | Zm00001eb095650 (168010946..168012325) | Uncharacterized protein |
|  |  |  |  |  |  |  |  | Zm00001eb095640 (168003513..168012351) | Protein LONGIFOLIA 2; DUF3741 domain-containing protein |
|  |  |  |  |  |  |  |  | Zm00001eb095660 (168132968..168136293) | Receptor-like kinase |
|  |  |  |  |  |  |  |  | Zm00001eb095670 (168222060..168223326) | Uncharacterized protein |
|  |  |  |  |  |  |  |  | Zm00001eb095680 (168247133..168247592) | Uncharacterized protein |
|  |  |  |  |  |  |  |  | Zm00001eb095700 (168276901..168293775) | DNA binding |
|  |  |  |  |  |  |  |  | Zm00001eb095710 (168388452..168390699) | CAX-interacting protein 4 |
|  |  |  |  |  |  |  |  | Zm00001eb095690 (168274815..168276647) | MYB transcription factor (Transcription factor MYB44) |
|  |  |  |  |  |  |  |  | Zm00001eb095720 (168434440..168435685) | Uncharacterized protein |
|  |  |  |  |  |  |  |  | Zm00001eb095740 (168603098..168604649) | CRAL-TRIO domain-containing protein |
|  |  |  |  |  |  |  |  | Zm00001eb095750 (168627694..168632100) | Dynamin-related protein 1E |
|  |  |  |  |  |  |  |  | Zm00001eb095760 (168711390..168712901) | Transcription factor RAX2 |
|  |  |  |  |  |  |  |  | Zm00001eb095730 (168531736..168535409) | Pre-mRNA-splicing factor 38 |
|  |  |  |  |  |  |  |  | Zm00001eb095770 (168769701..168773922) | p-loop NTPase domain-containing protein LPA1 homolog 1 |
|  |  |  |  |  |  |  |  | Zm00001eb095780 (168842246..168842644) | Ethylene-responsive transcription factor 14 |
|  |  |  |  |  |  |  |  | Zm00001eb095790 (168928780..168930621) | Phenolic glucoside malonyltransferase 1 |
|  |  |  |  |  |  |  |  | Zm00001eb095810 (169051114..169051736) | Ypt/Rab-GAP domain of gyp1p superfamily protein |
|  |  |  |  |  |  |  |  | Zm00001eb095830 (169185605..169186387) | Uncharacterized protein |
|  |  |  |  |  |  |  |  | Zm00001eb095850 (169208570..169208882) | SNF2 N-terminal domain-containing protein |
|  |  |  |  |  |  |  |  | Zm00001eb095860 (169208983..169209354) |  |
|  |  |  |  |  |  |  |  | Zm00001eb095880 (169346855..169352766) | C2H2-like zinc finger protein |
|  |  |  |  |  |  |  |  | Zm00001eb095890 (169354285..169359450) | U-box domain-containing protein 33; Protein kinase domain-containing protein |
|  |  |  |  |  |  |  |  | Zm00001eb095930 (169453179..169455530) | Genetic modifier (Haloacid dehalogenase-like hydrolase (HAD) superfamily protein) |
|  |  |  |  |  |  |  |  | Zm00001eb095870 (169325218..169326345) | Histone H2B |
|  |  |  |  |  |  |  |  | Zm00001eb095800 (169015055..169018298) | Fungal lipase-like domain-containing protein; Uncharacterized protein |
|  |  |  |  |  |  |  |  | Zm00001eb095840 (169185814..169188245) | ACT domain-containing protein ACR (Protein ACT DOMAIN REPEATS) |
|  |  |  |  |  |  |  |  | Zm00001eb095900 (169354724..169356032) | Uncharacterized protein |
|  |  |  |  |  |  |  |  | Zm00001eb095920 (169440636..169446060) | Calmodulin-binding heat-shock protein |
|  |  |  |  |  |  |  |  | Zm00001eb095910 (169395660..169396064) | Uncharacterized protein |
|  |  |  |  |  |  |  |  | Zm00001eb095940 (169496785..169499118) | Uncharacterized protein |
|  |  |  |  |  |  |  |  | Zm00001eb095950 (169534236..169534976) | Germin-like protein |
| PHP02 | MNH1.2020 | 4 | 92848382 | 92852889 | 92852889 | 5.53810066 | 1 |  |  |
|  |  |  |  |  |  |  |  | Zm00001eb180290 (92846894..92886790) | USP domain-containing protein |
| PHP02 | MNH1.2020 | 8 | 23433532 | 23444477 | 23444477 | 5.108709399 | 2 |  |  |
|  |  |  |  |  |  |  |  | Zm00001eb338010 (23433452..23440838) | Protein kinase domain-containing protein |
|  |  |  |  |  |  |  |  | Zm00001eb338020 (23440185..23444748) | Protein kinase superfamily protein |
| PHP02 | MNH1.2021 | 1 | 166280546 | 166305173 | 166304801 | 5.423942862 | 3 |  |  |
|  |  |  |  |  |  |  |  | Zm00001eb030220 (166280011..166293970) | CSC1-like protein HYP1 |
|  |  |  |  |  |  |  |  | Zm00001eb030240 (166298390..166300037) | AP2/ERF domain-containing protein |
|  |  |  |  |  |  |  |  | Zm00001eb030250 (166300052..166305189) | "GDP-Man:Man(3)GlcNAc(2)-PP-Dol alpha-1, 2-mannosyltransferase" |
| PHP02 | MNH1.2021 | 2 | 166182346 | 167386083 | 166660350 | 5.30108169 | 10 |  |  |
|  |  |  |  |  |  |  |  | Zm00001eb095460 (166182346..166182898) | Peptidase M16 C-terminal domain-containing protein |
|  |  |  |  |  |  |  |  | Zm00001eb095470 (166184962..166185931) | Peptidyl-prolyl cis-trans isomerase |
|  |  |  |  |  |  |  |  | Zm00001eb095480 (166273248..166288490) | Protein NEDD1; Anaphase-promoting complex subunit 4 WD40 domain-containing protein |
|  |  |  |  |  |  |  |  | Zm00001eb095500 (166373925..166383826) | Tyrosine-protein phosphatase; Putative tyrosine-protein phosphatase |
|  |  |  |  |  |  |  |  | Zm00001eb095510 (166516933..166522696) | Protein kinase domain-containing protein; Serine/threonine protein kinase 3 |
|  |  |  |  |  |  |  |  | Zm00001eb095520 (166549067..166549690) | Uncharacterized protein |
|  |  |  |  |  |  |  |  | Zm00001eb095530 (166658652..166664196) | ARM repeat superfamily protein |
|  |  |  |  |  |  |  |  | Zm00001eb095540 (166900507..166906461) | Polyadenylate-binding protein |
|  |  |  |  |  |  |  |  | Zm00001eb095550 (167021674..167022917) | Peroxidase |
|  |  |  |  |  |  |  |  | Zm00001eb095560 (167384845..167405370) | RNA binding |
| PHP02 | MNH1.2021 | 3 | 170917907 | 170925636 | 170919501 | 5.191487236 | 2 |  |  |
|  |  |  |  |  |  |  |  | Zm00001eb144360 (170915124..170918158) | Putative carboxylesterase 11 |
|  |  |  |  |  |  |  |  | Zm00001eb144370 (170918227..170925988) | Putative thimet oligopeptidase; Peptidase M3A/M3B catalytic domain-containing protein |
| PHP02 | MNH1.2021 | 4 | 89136868 | 89136868 | 89136868 | 5.158345521 | 1 |  |  |
|  |  |  |  |  |  |  |  | Zm00001eb180010 (89136211..89137174) | Uncharacterized protein |
| PHP02 | MNH1.2021 | 8 | 14203705 | 14492868 | 14325217 | 5.30660658 | 7 |  |  |
|  |  |  |  |  |  |  |  | Zm00001eb335690 (14203119..14206058) | Duplicated homeodomain-like superfamily protein (MYB-related transcription factor) |
|  |  |  |  |  |  |  |  | Zm00001eb335710 (14319884..14327098) | Protein NRT1/ PTR FAMILY 8.1 |
|  |  |  |  |  |  |  |  | Zm00001eb335730 (14354170..14360798) | Uncharacterized protein |
|  |  |  |  |  |  |  |  | Zm00001eb335740 (14395402..14417328) | Uncharacterized protein |
|  |  |  |  |  |  |  |  | Zm00001eb335800 (14489458..14493625) | Uncharacterized protein; S-adenosyl-L-methionine-dependent methyltransferase superfamily protein |
|  |  |  |  |  |  |  |  | Zm00001eb335790 (14485981..14488772) | Mitochondrial glycoprotein; Mitochondrial glycoprotein family protein |
|  |  |  |  |  |  |  |  | Zm00001eb335780 (14480413..14481351) | F-box protein |
| PHP02 | OHH1.2020 | 6 | 105778774 | 105827001 | 105827001 | 5.281857203 | 3 |  |  |
|  |  |  |  |  |  |  |  | Zm00001eb275020 (105778502..105780637) | Ubiquitin3 |
|  |  |  |  |  |  |  |  | Zm00001eb275030 (105781152..105784679) | PLD phosphodiesterase domain-containing protein |
|  |  |  |  |  |  |  |  | Zm00001eb275040 (105822734..105827038) | DNA-3-methyladenine glycosylase I |
| PHP02 | OHH1.2020 | 9 | 153330602 | 153456438 | 153330602 | 5.37526014 | 5 |  |  |
|  |  |  |  |  |  |  |  | Zm00001eb400230 (153330178..153331052) | Uncharacterized protein |
|  |  |  |  |  |  |  |  | Zm00001eb400240 (153340322..153349193) | Retrovirus-related Pol polyprotein LINE-1 |
|  |  |  |  |  |  |  |  | Zm00001eb400250 (153358658..153359814) | Uncharacterized protein |
|  |  |  |  |  |  |  |  | Zm00001eb400260 (153359858..153360199) | Uncharacterized protein |
|  |  |  |  |  |  |  |  | Zm00001eb400270 (153434131..153460825) | DExH-box ATP-dependent RNA helicase DExH14 |
| PHP02 | WIH1.2020 | 3 | 181216311 | 181244189 | 181216311 | 5.054871922 | 4 |  |  |
|  |  |  |  |  |  |  |  | Zm00001eb146990 (181215611..181225995) | Uncharacterized protein |
|  |  |  |  |  |  |  |  | Zm00001eb147000 (181224798..181225741) | Uncharacterized protein |
|  |  |  |  |  |  |  |  | Zm00001eb147010 (181227276..181228749) | Duplicated homeodomain-like superfamily protein |
|  |  |  |  |  |  |  |  | Zm00001eb147020 (181242221..181244205) | Uncharacterized protein |
| PHP02 | WIH1.2021 | 7 | 122827200 | 122829167 | 122828618 | 5.06393243 | 1 |  |  |
|  |  |  |  |  |  |  |  | Zm00001eb313290 (122826234..122837217) | Folate-biopterin transporter 6 |
| PHP02 | WIH2.2021 | 6 | 117143559 | 117202756 | 117143559 | 5.512110485 | 2 |  |  |
|  |  |  |  |  |  |  |  | Zm00001eb278010 (117143009..117143722) | Cytoplasmic tRNA 2-thiolation protein 1 |
|  |  |  |  |  |  |  |  | Zm00001eb278020 (117195391..117204329) | riboflavin kinase |
| PJP02 | WIH2.2021 | 7 | 141743512 | 141802284 | 141774354 | 5.815156073 | 6 |  |  |
|  |  |  |  |  |  |  |  | Zm00001eb317410 (141738901..141745064) | Type II inositol polyphosphate 5-phosphatase 15 |
|  |  |  |  |  |  |  |  | Zm00001eb317420 (141747209..141764376) | DNA topoisomerase |
|  |  |  |  |  |  |  |  | Zm00001eb317430 (141766558..141768266) | Cytochrome b561 and DOMON domain-containing protein |
|  |  |  |  |  |  |  |  | Zm00001eb317440 (141770375..141771645) | Auxin-responsive family protein |
|  |  |  |  |  |  |  |  | Zm00001eb317450 (141773763..141775738) |  |
|  |  |  |  |  |  |  |  | Zm00001eb317460 (141800352..141802904) | BHLH domain-containing protein; Transcription factor bHLH62; BHLH transcription factor |
| PHP02 | WIH2.2021 | 9 | 126299270 | 126331089 | 126299270 | 5.344739423 | 2 |  |  |
|  |  |  |  |  |  |  |  | Zm00001eb392560 (126298389..126301301) | hydroxyisourate hydrolase |
|  |  |  |  |  |  |  |  | Zm00001eb392570 (126309043..126331353) | Cytochrome c oxidase subunit 6b-1; Uncharacterized protein |
| PHP02 | WIH2.2021 | 10 | 131242110 | 131242110 | 131242110 | 5.18454783 | 1 |  |  |
|  |  |  |  |  |  |  |  | Zm00001eb426010 (131239331..131242574) | Uncharacterized protein |
|  |  |  |  |  |  |  |  |  |  |
| PHP02 | WIH3.2021 | 7 | 165183006 | 165183006 | 165183006 | 5.014355252 | 1 |  |  |
|  |  |  |  |  |  |  |  | Zm00001eb323710 (165182615..165187497) | Cellulose synthase-like protein D3; Mixed-linked glucan synthase 8 |
| PHP02 | WIH3.2021 | 8 | 180304727 | 180305822 | 180305579 | 5.185710027 | 2 |  |  |
|  |  |  |  |  |  |  |  | Zm00001eb370630 (180304325..180306412) | Uncharacterized protein |
|  |  |  |  |  |  |  |  | Zm00001eb370640 (180304434..180306375) | Interactor of constitutive active ROPs 1 |
| PHP02 | WIH3.2021 | 9 | 21040087 | 21045888 | 21040087 | 5.273213824 | 1 |  |  |
|  |  |  |  |  |  |  |  | Zm00001eb376840 (21039872..21045940) | Uncharacterized protein; NB-ARC domain-containing protein |
| PHZ51 | DEH1.2021 | 4 | 11862714 | 11864631 | 11862714 | 5.002933081 | 1 |  |  |
|  |  |  |  |  |  |  |  | Zm00001eb168010 (11862186..11864645) | protein-serine/threonine phosphatase |
| PHZ51 | DEH1.2021 | 5 | 18905027 | 19008675 | 19005611 | 5.141092691 | 3 |  |  |
|  |  |  |  |  |  |  |  | Zm00001eb218960 (18904784..18910008) | Cytochrome P450 family 87 subfamily A polypeptide 2 |
|  |  |  |  |  |  |  |  | Zm00001eb218970 (18951226..18956858) | Cytochrome P450 family 87 subfamily A polypeptide 2 |
|  |  |  |  |  |  |  |  | Zm00001eb218980 (19005607..19008831) | Uncharacterized protein |
| PHZ51 | DEH1.2021 | 6 | 165359429 | 165515724 | 165382550 | 5.265256879 | 8 |  |  |
|  |  |  |  |  |  |  |  | Zm00001eb290640 (165359333..165360286) | Uncharacterized protein |
|  |  |  |  |  |  |  |  | Zm00001eb290650 (165361305..165367583) | Uncharacterized protein |
|  |  |  |  |  |  |  |  | Zm00001eb290690 (165381210..165382911) | Probable zinc-ribbon domain-containing protein |
|  |  |  |  |  |  |  |  | Zm00001eb290680 (165378046..165378453) |  |
|  |  |  |  |  |  |  |  | Zm00001eb290670 (165372902..165376674) | Protein kinase domain-containing protein |
|  |  |  |  |  |  |  |  | Zm00001eb290700 (165490528..165513964) | Protein kinase domain-containing protein |
|  |  |  |  |  |  |  |  | Zm00001eb290710 (165509806..165513534) | Uncharacterized protein |
|  |  |  |  |  |  |  |  | Zm00001eb290720 (165514145..165517216) | Protein kinase domain-containing protein |
| PHZ51 | GAH1.2021 | 4 | 10898390 | 10899295 | 10899295 | 5.439165294 | 1 |  |  |
|  |  |  |  |  |  |  |  | Zm00001eb167780 (10897637..10905132) | Leucine-rich repeat protein kinase family protein |
| PHZ51 | GAH1.2021 | 5 | 13102195 | 13103157 | 13102195 | 5.019034597 | 1 |  |  |
|  |  |  |  |  |  |  |  | Zm00001eb216980 (13101136..13105398) | Late embryogenesis abundant (LEA) hydroxyproline-rich glycoprotein family; Late embryogenesis abundant protein LEA-2 subgroup domain-containing protein |
| PHZ51 | GAH1.2021 | 10 | 1409076 | 1409076 | 1409076 | 5.028974368 | 1 |  |  |
|  |  |  |  |  |  |  |  | Zm00001eb405060 (1405898..1409262) | Regulatory protein NPR5; Regulatory protein NPR central domain-containing protein; BTB domain-containing protein |
| PHZ51 | IAH1.2021 | 10 | 4309382 | 4411934 | 4353431 | 5.798635539 | 4 |  |  |
|  |  |  |  |  |  |  |  | Zm00001eb406490 (4309015..4309712) | AT3g10020/T22K18_16-like protein |
|  |  |  |  |  |  |  |  | Zm00001eb406500 (4318424..4319026) | Uncharacterized protein |
|  |  |  |  |  |  |  |  | Zm00001eb406510 (4353006..4355156) | DUF668 family protein |
|  |  |  |  |  |  |  |  | Zm00001eb406520 (4405042..4413573) | DDT domain-containing protein PTM |
| PHZ51 | IAH2.2021 | 9 | 26394490 | 26396426 | 26394490 | 5.636060036 | 1 |  |  |
|  |  |  |  |  |  |  |  | Zm00001eb378420 (26394203..26396821) | UV-stimulated scaffold protein A; UV-stimulated scaffold protein A-like protein |
| PHZ51 | IAH2.2021 | 10 | 4309382 | 4411934 | 4407656 | 5.508747158 | 4 |  |  |
|  |  |  |  |  |  |  |  | Zm00001eb406490 (4309015..4309712) | AT3g10020/T22K18_16-like protein |
|  |  |  |  |  |  |  |  | Zm00001eb406500 (4318424..4319026) | Uncharacterized protein |
|  |  |  |  |  |  |  |  | Zm00001eb406510 (4353006..4355156) | DUF668 family protein |
|  |  |  |  |  |  |  |  | Zm00001eb406520 (4405042..4413573) | DDT domain-containing protein PTM |
| PHZ51 | IAH3.2021 | 2 | 19708984 | 19752976 | 19708984 | 5.488269788 | 3 |  |  |
|  |  |  |  |  |  |  |  | Zm00001eb074290 (19708255..19710540) | Subtilisin-like protease SBT1.9 |
|  |  |  |  |  |  |  |  | Zm00001eb074300 (19717708..19718562) | Peptidase S8/S53 domain-containing protein |
|  |  |  |  |  |  |  |  | Zm00001eb074310 (19752930..19753818) | Uncharacterized protein |
| PHZ51 | IAH3.2021 | 3 | 227318635 | 228194509 | 227547488 | 6.29812358 | 22 |  |  |
|  |  |  |  |  |  |  |  | Zm00001eb160830 (227318382..227326524) | Aldehyde dehydrogenase5 |
|  |  |  |  |  |  |  |  | Zm00001eb160840 (227325522..227326490) | Uncharacterized protein |
|  |  |  |  |  |  |  |  | Zm00001eb160850 (227362644..227374171) | 40S ribosomal protein S23 |
|  |  |  |  |  |  |  |  | Zm00001eb160860 (227374548..227375177) |  |
|  |  |  |  |  |  |  |  | Zm00001eb160870 (227375398..227375889) |  |
|  |  |  |  |  |  |  |  | Zm00001eb160880 (227376606..227378915) | Nodulin-like domain-containing protein |
|  |  |  |  |  |  |  |  | Zm00001eb160890 (227378917..227379386) | Uncharacterized protein |
|  |  |  |  |  |  |  |  | Zm00001eb160900 (227379372..227380712) | Uncharacterized protein |
|  |  |  |  |  |  |  |  | Zm00001eb160920 (227426612..227427982) | Uncharacterized protein; Transmembrane protein |
|  |  |  |  |  |  |  |  | Zm00001eb160910 (227384479..227385922) | Uncharacterized protein |
|  |  |  |  |  |  |  |  | Zm00001eb160940 (227481274..227482692) | Uncharacterized protein |
|  |  |  |  |  |  |  |  | Zm00001eb160960 (227502190..227502692) | Uncharacterized protein |
|  |  |  |  |  |  |  |  | Zm00001eb160970 (227503993..227505501) | Uncharacterized protein |
|  |  |  |  |  |  |  |  | Zm00001eb160990 (227531207..227543775) | Pentatricopeptide repeat-containing protein |
|  |  |  |  |  |  |  |  | Zm00001eb161010 (227583442..227584158) | Transcription repressor (Ovate family protein) |
|  |  |  |  |  |  |  |  | Zm00001eb161030 (227681402..227713213) | Helicase ATP-binding domain-containing protein; ATP-dependent helicase C-terminal domain-containing protein |
|  |  |  |  |  |  |  |  | Zm00001eb161040 (228015205..228093433) | Zinc finger FYVE domain-containing protein 26; Uncharacterized protein |
|  |  |  |  |  |  |  |  | Zm00001eb161050 (228162086..228164481) | FLZ-type domain-containing protein; MARD1 |
|  |  |  |  |  |  |  |  | Zm00001eb161060 (228172619..228173088) | Uncharacterized protein |
|  |  |  |  |  |  |  |  | Zm00001eb161070 (228173292..228173849) | Uncharacterized protein |
|  |  |  |  |  |  |  |  | Zm00001eb161080 (228178498..228183335) | Protein SYS1 |
|  |  |  |  |  |  |  |  | Zm00001eb161090 (228193564..228194688) | N-acetyltransferase domain-containing protein |
| PHZ51 | IAH3.2021 | 5 | 167632182 | 167774211 | 167774211 | 5.069152231 | 4 |  |  |
|  |  |  |  |  |  |  |  | Zm00001eb241860 (167630103..167661905) | DNA polymerase epsilon catalytic subunit |
|  |  |  |  |  |  |  |  | Zm00001eb241870 (167665263..167666531) | SAUR-like auxin-responsive protein family |
|  |  |  |  |  |  |  |  | Zm00001eb241880 (167763886..167775432) | LITAF domain-containing protein |
|  |  |  |  |  |  |  |  | Zm00001eb241890 (167767503..167770515) | Uncharacterized protein |
| PHZ51 | IAH3.2021 | 9 | 95693487 | 96174446 | 95695889 | 5.108623649 | 9 |  |  |
|  |  |  |  |  |  |  |  | Zm00001eb386390 (95693470..95696583) | EIN3-binding F-box protein 1 |
|  |  |  |  |  |  |  |  | Zm00001eb386410 (95704137..95716811) | non-specific serine/threonine protein kinase |
|  |  |  |  |  |  |  |  | Zm00001eb386420 (95921125..95921445) | F-box domain-containing protein |
|  |  |  |  |  |  |  |  | Zm00001eb386430 (95986709..95989633) | F-box domain-containing protein |
|  |  |  |  |  |  |  |  | Zm00001eb386440 (96088780..96090931) | F-box/LRR-repeat protein 23 |
|  |  |  |  |  |  |  |  | Zm00001eb386450 (96090984..96097630) | NET domain-containing protein |
|  |  |  |  |  |  |  |  | Zm00001eb386460 (96091965..96093519) | Uncharacterized protein |
|  |  |  |  |  |  |  |  | Zm00001eb386480 (96168093..96170362) | Tetratricopeptide repeat (TPR)-like superfamily protein |
|  |  |  |  |  |  |  |  | Zm00001eb386490 (96168985..96174897) | TPX2 C-terminal domain-containing protein; Protein WVD2-like 5 |
| PHZ51 | IAH4.2021 | 4 | 8017896 | 8017896 | 8017896 | 5.207776139 | 1 |  |  |
|  |  |  |  |  |  |  |  | Zm00001eb167380 (8017637..8018421) | Uncharacterized protein |
| PHZ51 | IAH4.2021 | 8 | 168868442 | 168869401 | 168868967 | 5.049786887 | 1 |  |  |
|  |  |  |  |  |  |  |  | Zm00001eb364850 (168865502..168869455) | BHLH domain-containing protein; BHLH transcription factor |
| PHZ51 | NCH1.2021 | 2 | 20116723 | 20126054 | 20117166 | 5.043411136 | 1 |  |  |
|  |  |  |  |  |  |  |  | Zm00001eb074380 (20116514..20126542) | Putative copper-transporting ATPase HMA5; Copper-transporting ATPase HMA5 |
| PHZ51 | NEH1.2020 | 2 | 3368599 | 3379597 | 3379597 | 5.096030065 | 2 |  |  |
|  |  |  |  |  |  |  |  | Zm00001eb067180 (3368183..3374940) | leucine--tRNA ligase |
|  |  |  |  |  |  |  |  | Zm00001eb067190 (3376438..3379835) | Myb family transcription factor PHL5; HTH myb-type domain-containing protein |
| PHZ51 | NEH1.2020 | 3 | 10569804 | 11214938 | 11035729 | 5.364228572 | 13 |  |  |
|  |  |  |  |  |  |  |  | Zm00001eb122560 (10569254..10572857) | Uncharacterized protein |
|  |  |  |  |  |  |  |  | Zm00001eb122570 (10575440..10579236) | NPH3 domain-containing protein |
|  |  |  |  |  |  |  |  | Zm00001eb122590 (10592078..10594847) | Uncharacterized protein |
|  |  |  |  |  |  |  |  | Zm00001eb122580 (10591538..10592017) | Uncharacterized protein |
|  |  |  |  |  |  |  |  | Zm00001eb122600 (10670245..10672932) | Hyccin |
|  |  |  |  |  |  |  |  | Zm00001eb122610 (10672744..10675487) | Glycosyltransferase |
|  |  |  |  |  |  |  |  | Zm00001eb122620 (10681696..10683755) | Glycosyltransferase (EC 2.4.1.-); Uncharacterized protein |
|  |  |  |  |  |  |  |  | Zm00001eb122630 (10830992..10832903) | Glycosyltransferase |
|  |  |  |  |  |  |  |  | Zm00001eb122640 (10833953..10834711) | Uncharacterized protein |
|  |  |  |  |  |  |  |  | Zm00001eb122650 (10949677..10950652) | Uncharacterized protein |
|  |  |  |  |  |  |  |  | Zm00001eb122660 (10952187..10959287) | Boron transporter-like protein 2 |
|  |  |  |  |  |  |  |  | Zm00001eb122670 (11035421..11036332) | Uncharacterized protein |
|  |  |  |  |  |  |  |  | Zm00001eb122680 (11212309..11216964) | Cytochrome P450 734A1 |
| PHZ51 | NEH1.2020 | 4 | 5528689 | 5529942 | 5528689 | 5.239261397 | 1 |  |  |
|  |  |  |  |  |  |  |  | Zm00001eb166230 (5528494..5530489) | Putative polyol transporter 1 |
| PHZ51 | NEH1.2020 | 5 | 9625767 | 10224189 | 10224189 | 6.544154578 | 21 |  |  |
|  |  |  |  |  |  |  |  | Zm00001eb215610 (9625240..9629237) | BHLH domain-containing protein; Transcription factor bHLH62 |
|  |  |  |  |  |  |  |  | Zm00001eb215620 (9687937..9699401) | ATP-dependent RNA helicase |
|  |  |  |  |  |  |  |  | Zm00001eb215640 (9698957..9701413) | Uncharacterized protein; DNA binding |
|  |  |  |  |  |  |  |  | Zm00001eb215650 (9702544..9706836) | J domain-containing protein; Chaperone DnaJ-domain superfamily protein (Heat shock protein binding protein) |
|  |  |  |  |  |  |  |  | Zm00001eb215660 (9752861..9757248) | E3 ubiquitin-protein ligase FANCL; Zinc ion binding; RWD domain-containing protein |
|  |  |  |  |  |  |  |  | Zm00001eb215670 (9853208..9856504) | Uncharacterized protein |
|  |  |  |  |  |  |  |  | Zm00001eb215680 (9899820..9905037) | CSC1-like protein RXW8 |
|  |  |  |  |  |  |  |  | Zm00001eb215690 (9954675..9955438) | Uncharacterized protein |
|  |  |  |  |  |  |  |  | Zm00001eb215700 (9991095..9992055) | SOUL heme-binding family protein |
|  |  |  |  |  |  |  |  | Zm00001eb215710 (9999369..10002552) | Tubulin alpha chain |
|  |  |  |  |  |  |  |  | Zm00001eb215720 (10130661..10131833) | DUF630 domain-containing protein |
|  |  |  |  |  |  |  |  | Zm00001eb215730 (10132077..10132530) | DUF632 domain-containing protein |
|  |  |  |  |  |  |  |  | Zm00001eb215740 (10136352..10138887) | Symbiotic ammonium transporter |
|  |  |  |  |  |  |  |  | Zm00001eb215750 (10141733..10144991) | Protein kinase domain-containing protein |
|  |  |  |  |  |  |  |  | Zm00001eb215760 (10146997..10147557) | WPP domain-containing protein |
|  |  |  |  |  |  |  |  | Zm00001eb215770 (10148509..10149137) | Uncharacterized protein |
|  |  |  |  |  |  |  |  | Zm00001eb215780 (10155216..10159969) | Lariat debranching enzyme |
|  |  |  |  |  |  |  |  | Zm00001eb215790 (10167739..10173908) | Protein kinase domain-containing protein |
|  |  |  |  |  |  |  |  | Zm00001eb215800 (10181520..10184538) | Uncharacterized protein |
|  |  |  |  |  |  |  |  | Zm00001eb215810 (10185449..10187611) | Maf-like protein CV_0124 |
|  |  |  |  |  |  |  |  | Zm00001eb215820 (10205720..10224396) | FIP1[V]-like protein |
| PHZ51 | NEH1.2021 | 2 | 32714329 | 34064402 | 32730052 | 6.163959669 | 35 |  |  |
|  |  |  |  |  |  |  |  | Zm00001eb078100 (32714200..32723073) | Leucine-rich repeat family protein |
|  |  |  |  |  |  |  |  | Zm00001eb078110 (32729608..32737169) | Calcium-binding EF hand family protein; EF-hand domain-containing protein |
|  |  |  |  |  |  |  |  | Zm00001eb078120 (32768952..32788400) | Major facilitator superfamily protein; Nodulin-like domain-containing protein |
|  |  |  |  |  |  |  |  | Zm00001eb078130 (32839664..32842500) | 40S ribosomal protein S11 N-terminal domain-containing protein |
|  |  |  |  |  |  |  |  | Zm00001eb078140(32870011..32878172) | Trafficking protein particle complex II-specific subunit 120-like protein |
|  |  |  |  |  |  |  |  | Zm00001eb078150 (32878800..32879662) | t-SNARE coiled-coil homology domain-containing protein |
|  |  |  |  |  |  |  |  | Zm00001eb078160 (32918154..32919348) | HMA domain-containing protein |
|  |  |  |  |  |  |  |  | Zm00001eb078170 (33011767..33018261) | Sister chromatid cohesion protein PDS5 B-B |
|  |  |  |  |  |  |  |  | Zm00001eb078190 (33151454..33154775) | DNA glycosylase superfamily protein; DNA-3-methyladenine glycosylase I |
|  |  |  |  |  |  |  |  | Zm00001eb078200 (33157327..33162559) | Magnesium transporter |
|  |  |  |  |  |  |  |  | Zm00001eb078210 (33207589..33209622) | Ribosomal protein L23/L25 N-terminal domain-containing protein; 60S ribosomal protein L23a |
|  |  |  |  |  |  |  |  | Zm00001eb078220 (33379891..33386009) | Shikimate O-hydroxycinnamoyltransferase |
|  |  |  |  |  |  |  |  | Zm00001eb078230 (33439003..33439593) | Uncharacterized protein |
|  |  |  |  |  |  |  |  | Zm00001eb078240 (33440386..33440793) | Uncharacterized protein |
|  |  |  |  |  |  |  |  | Zm00001eb078250 (33442427..33443391) | Zinc finger CCCH domain-containing protein 22 |
|  |  |  |  |  |  |  |  | Zm00001eb078280 (33485604..33486772) | GPI-anchored protein |
|  |  |  |  |  |  |  |  | Zm00001eb078290 (33487363..33489618) | Cytochrome P450 71A26 |
|  |  |  |  |  |  |  |  | Zm00001eb078300 (33490730..33498659) | Tubulin/FtsZ GTPase domain-containing protein |
|  |  |  |  |  |  |  |  | Zm00001eb078320 (33508439..33510487) | Cytochrome P450 71A26 |
|  |  |  |  |  |  |  |  | Zm00001eb078330 (33592210..33596785) | Uncharacterized protein |
|  |  |  |  |  |  |  |  | Zm00001eb078340 (33600173..33602240) | Cytochrome P450 CYP71C36 |
|  |  |  |  |  |  |  |  | Zm00001eb078350 (33817057..33822483) | Eukaryotic translation initiation factor isoform 4G-2 |
|  |  |  |  |  |  |  |  | Zm00001eb078360 (33827014..33830293) | Enhancer of rudimentary homolog |
|  |  |  |  |  |  |  |  | Zm00001eb078370 (33831377..33835361) | Folate-biopterin transporter 2 |
|  |  |  |  |  |  |  |  | Zm00001eb078380 (33834954..33835361) | Pre-mRNA-splicing factor 18 |
|  |  |  |  |  |  |  |  | Zm00001eb078390 (33897655..33900394) | N-acetyltransferase ESCO1 (Protein CHROMOSOME TRANSMISSION FIDELITY 7) |
|  |  |  |  |  |  |  |  | Zm00001eb078410 (34008734..34012060) | Protein CHROMOSOME TRANSMISSION FIDELITY 7 |
|  |  |  |  |  |  |  |  | Zm00001eb078400 (33969079..33972745) | Protein CHROMOSOME TRANSMISSION FIDELITY 7 |
|  |  |  |  |  |  |  |  | Zm00001eb078420 (34022858..34023375) |  |
|  |  |  |  |  |  |  |  | Zm00001eb078430 (34025361..34026186) |  |
|  |  |  |  |  |  |  |  | Zm00001eb078440 (34034310..34037394) | S-adenosylmethionine decarboxylase proenzyme |
|  |  |  |  |  |  |  |  | Zm00001eb078450 (34039586..34041412) | 14-3-3 domain-containing protein |
|  |  |  |  |  |  |  |  | Zm00001eb078460 (34050232..34052019) | "ATP synthase subunit e, mitochondrial" |
|  |  |  |  |  |  |  |  | Zm00001eb078470 (34058765..34061601) | VAN3-binding protein; VAN3-binding protein-like auxin canalisation domain-containing protein |
|  |  |  |  |  |  |  |  | Zm00001eb078480 (34063699..34065949) | OSJNBa0067K08.20-like protein |
| PHZ51 | NEH1.2021 | 3 | 8495672 | 8574759 | 8557242 | 5.320309257 | 7 |  |  |
|  |  |  |  |  |  |  |  | Zm00001eb121760 (8492142..8495789) | C2H2 and C2HC zinc fingers superfamily protein |
|  |  |  |  |  |  |  |  | Zm00001eb121770 (8497540..8499728) | Uncharacterized protein |
|  |  |  |  |  |  |  |  | Zm00001eb121780 (8497640..8499737) | Phospholipase A1-Igamma1 chloroplastic |
|  |  |  |  |  |  |  |  | Zm00001eb121790 (8549086..8549650) | Mitogen-activated protein kinase 1 |
|  |  |  |  |  |  |  |  | Zm00001eb121810 (8556384..8562556) | glutamine--tRNA ligase |
|  |  |  |  |  |  |  |  | Zm00001eb121820 (8565662..8567478) | Pentatricopeptide repeat-containing protein |
|  |  |  |  |  |  |  |  | Zm00001eb121840 (8569190..8575058) | DUF1664 domain-containing protein |
| PHZ51 | NEH1.2021 | 4 | 11874080 | 12096621 | 11964580 | 5.442622418 | 2 |  |  |
|  |  |  |  |  |  |  |  | Zm00001eb168040 (11964369..11965713) | Putative AP2/EREBP transcription factor superfamily protein |
|  |  |  |  |  |  |  |  | Zm00001eb168050 (12089130..12096668) | Non-reducing end alpha-L-arabinofuranosidase |
| PHZ51 | NEH1.2021 | 7 | 144447089 | 145331868 | 145109437 | 9.020905081 | 25 |  |  |
|  |  |  |  |  |  |  |  | Zm00001eb318160 (144446656..144451778) | Nucleotide exchange factor Fes1 domain-containing protein |
|  |  |  |  |  |  |  |  | Zm00001eb318170 (144527754..144530955) | Cobalt ion binding (SnRK1-interacting protein 1) |
|  |  |  |  |  |  |  |  | Zm00001eb318180 (144532753..144535864) | Plasma membrane |
|  |  |  |  |  |  |  |  | Zm00001eb318190 (144558083..144558736) | Uncharacterized protein |
|  |  |  |  |  |  |  |  | Zm00001eb318200 (144562952..144566800) | Ankyrin repeat domain-containing protein 2A; STI1/HOP DP domain-containing protein; Ankyrin repeat domain-containing protein 2 |
|  |  |  |  |  |  |  |  | Zm00001eb318210 (144574636..144592063) | ATP-dependent RNA helicase DRS1 |
|  |  |  |  |  |  |  |  | Zm00001eb318220 (144649738..144652105) | Transmembrane protein |
|  |  |  |  |  |  |  |  | Zm00001eb318230 (144666038..144670524) | Phospholipase A(1) LCAT3 |
|  |  |  |  |  |  |  |  | Zm00001eb318240 (144689142..144700012) | 3-hydroxyisobutyryl-CoA hydrolase |
|  |  |  |  |  |  |  |  | Zm00001eb318250 (144720678..144721995) | Galacturonosyltransferase-like 9 |
|  |  |  |  |  |  |  |  | Zm00001eb318260 (144722791..144723432) | Polymer-forming cytoskeletal protein |
|  |  |  |  |  |  |  |  | Zm00001eb318270 (144729270..144730271) | HEN1 double-stranded RNA binding domain-containing protein |
|  |  |  |  |  |  |  |  | Zm00001eb318280 (144730355..144730930) | Small RNA 2'-O-methyltransferase |
|  |  |  |  |  |  |  |  | Zm00001eb318290 (144853910..144863036) | Protein FLOWERING LOCUS T |
|  |  |  |  |  |  |  |  | Zm00001eb318300 (144875910..144880623) | Protein kinase domain-containing protein; PTI1-like tyrosine-protein kinase 3 |
|  |  |  |  |  |  |  |  | Zm00001eb318310 (144992297..144996111) | U11/U12 small nuclear ribonucleoprotein 35 kDa protein |
|  |  |  |  |  |  |  |  | Zm00001eb318320 (145083368..145094038) | FAD-dependent oxidoreductase family protein; FAD dependent oxidoreductase domain-containing protein |
|  |  |  |  |  |  |  |  | Zm00001eb318330 (145091976..145095427) | Calcium-dependent protein kinase 24 |
|  |  |  |  |  |  |  |  | Zm00001eb318340 (145107432..145112668) | Aleurone layer morphogenesis protein; Uncharacterized protein |
|  |  |  |  |  |  |  |  | Zm00001eb318350 (145113443..145115972) | Ferredoxin |
|  |  |  |  |  |  |  |  | Zm00001eb318360 (145114839..145115956) | Uncharacterized protein |
|  |  |  |  |  |  |  |  | Zm00001eb318370 (145174164..145185159) | SAP domain-containing protein |
|  |  |  |  |  |  |  |  | Zm00001eb318380 (145186233..145187090) | Ribosomal protein L2 C-terminal domain-containing protein |
|  |  |  |  |  |  |  |  | Zm00001eb318390 (145323466..145331935) | Bromo domain-containing protein; Bromodomain protein 103 |
|  |  |  |  |  |  |  |  | Zm00001eb318400 (145332449..145338302) | Proteasome subunit beta |
| PHZ51 | NEH2.2020 | 4 | 178880087 | 178883028 | 178883028 | 5.823397217 | 2 |  |  |
|  |  |  |  |  |  |  |  | Zm00001eb192320 (178879941..178880886) | Putative AP2/EREBP transcription factor superfamily protein |
|  |  |  |  |  |  |  |  | Zm00001eb192330 (178881791..178884815) | Uncharacterized protein |
| PHZ51 | NEH2.2020 | 9 | 24183511 | 24273108 | 24271337 | 5.213722718 | 3 |  |  |
|  |  |  |  |  |  |  |  | Zm00001eb377910 (24180012..24183594) | Glycine-rich protein |
|  |  |  |  |  |  |  |  | Zm00001eb377920 (24183812..24187856) | Aminomethyltransferase |
|  |  |  |  |  |  |  |  | Zm00001eb377930 (24265618..24273109) | Uncharacterized protein |
| PHZ51 | NEH2.2020 | 10 | 49878002 | 51080942 | 49993974 | 5.060951866 | 7 |  |  |
|  |  |  |  |  |  |  |  | Zm00001eb412460 (49873378..49880857) | (DL)-glycerol-3-phosphatase 2 |
|  |  |  |  |  |  |  |  | Zm00001eb412470 (49993528..49994516) | Uncharacterized protein |
|  |  |  |  |  |  |  |  | Zm00001eb412480 (50065481..50066198) | Uncharacterized protein |
|  |  |  |  |  |  |  |  | Zm00001eb412490 (50588872..50600807) | Protein RAE1 (Rae1-like protein) |
|  |  |  |  |  |  |  |  | Zm00001eb412500 (50698736..50709309) | Bifunctional dethiobiotin synthetase/78-diamino-pelargonic acid aminotransferase mitochondrial |
|  |  |  |  |  |  |  |  | Zm00001eb412510 (50783116..50794973) | Transcription factor TGA4; DOG1 domain-containing protein; BZIP transcription factor |
|  |  |  |  |  |  |  |  | Zm00001eb412530 (51075230..51082778) | Uncharacterized protein |
| PHZ51 | NEH3.2020 | 1 | 227406565 | 227416565 | 227410131 | 5.03695784 | 2 |  |  |
|  |  |  |  |  |  |  |  | Zm00001eb043060 (227406373..227410137) | Pentatricopeptide repeat-containing protein chloroplastic |
|  |  |  |  |  |  |  |  | Zm00001eb043070 (227412712..227417072) | Oligosaccharide transporter |
| PHZ51 | NEH3.2020 | 10 | 9234038 | 9234851 | 9234038 | 5.073419741 | 1 |  |  |
|  |  |  |  |  |  |  |  | Zm00001eb407810 (9232979..9235197) | GATA transcription factor 14; GATA-type domain-containing protein |
| PHZ51 | TXH1.2020 | 2 | 19822843 | 19827155 | 19827155 | 5.791918848 | 1 |  |  |
|  |  |  |  |  |  |  |  | Zm00001eb074320 (19822666..19827464) | Regulatory protein |
| PHZ51 | TXH1.2020 | 4 | 23287954 | 23296035 | 23290605 | 5.613823498 | 3 |  |  |
|  |  |  |  |  |  |  |  | Zm00001eb170100 (23287485..23290901) | Uncharacterized protein |
|  |  |  |  |  |  |  |  | Zm00001eb170110 (23287546..23290688) | Protein SCARECROW |
|  |  |  |  |  |  |  |  | Zm00001eb170120 (23294630..23299828) | Protein kinase domain-containing protein |
| PHZ51 | TXH2.2021 | 8 | 9316382 | 9317504 | 9317504 | 5.00477245 | 1 |  |  |
|  |  |  |  |  |  |  |  | Zm00001eb334630 (9316123..10067523) | Protein kinase domain-containing protein; Cysteine-rich receptor-like protein kinase 37; Receptor-like kinase4; Wall-associated receptor kinase galacturonan-binding domain-containing protein |
| PHZ51 | WIH2.2020 | 5 | 20821091 | 20825659 | 20823419 | 5.280284248 | 3 |  |  |
|  |  |  |  |  |  |  |  | Zm00001eb219660 (20820726..20823562) | Uncharacterized protein |
|  |  |  |  |  |  |  |  | Zm00001eb219670 (20821696..20823574) | Uncharacterized protein |
|  |  |  |  |  |  |  |  | Zm00001eb219680 (20825618..20830864) | Peroxisomal membrane 22 kDa (Mpv17/PMP22) family protein |
| PHZ51 | WIH2.2020 | 6 | 174219648 | 174247550 | 174247550 | 5.333582885 | 2 |  |  |
|  |  |  |  |  |  |  |  | Zm00001eb294940 (174219065..174220336) | DUF1639 family protein |
|  |  |  |  |  |  |  |  | Zm00001eb294950 (174246198..174247711) | Uncharacterized protein |
| PHZ51 | WIH2.2020 | 8 | 13769510 | 13829286 | 13829286 | 5.086610414 | 3 |  |  |
|  |  |  |  |  |  |  |  | Zm00001eb335590 (13768096..13771513) | Octicosapeptide/Phox/Bem1p family protein |
|  |  |  |  |  |  |  |  | Zm00001eb335600 (13823967..13827047) | Phosphatidic acid phosphatase type 2/haloperoxidase domain-containing protein; Lipid phosphate phosphatase 3 |
|  |  |  |  |  |  |  |  | Zm00001eb335610 (13828595..13829456) | Uncharacterized protein |
| PHZ51 | WIH2.2021 | 2 | 210634672 | 210645236 | 210644392 | 5.112541404 | 1 |  |  |
|  |  |  |  |  |  |  |  | Zm00001eb106360 (210634514..210645250) | Villin-4; HP domain-containing protein; Gelsolin-like domain-containing protein |
| PHZ51 | WIH2.2021 | 5 | 163943883 | 164015944 | 163943883 | 5.118335014 | 3 |  |  |
|  |  |  |  |  |  |  |  | Zm00001eb241090 (163943648..163945453) |  |
|  |  |  |  |  |  |  |  | Zm00001eb241100 (163986357..163987295) | DUF679 domain membrane protein 7 |
|  |  |  |  |  |  |  |  | Zm00001eb241110 (164015846..164019359) | RS21-C6 protein |

Supplemental Table 2 (S2): All quantitative trait loci (QTLs) discovered along with candidate genes and their functions for grain yield slope and intercept for three different maize populations with PHK76, PHP02 and PHZ51 testers.

| Tester | Trait | Chr | Start | End | Peak | Peak LOD | No. Genes in Interval | Gene | Function |
| --- | --- | --- | --- | --- | --- | --- | --- | --- | --- |
| PHK76 | Slope | 7 | 142051425 | 142271481 | 142231205 | 6.025259231 | 11 |  |  |
|  |  |  |  |  |  |  |  | Zm00001eb317510 (142050974..142053196) | Chloroplastic quinone-oxidoreductase; Alcohol dehydrogenase-like N-terminal domain-containing protein |
|  |  |  |  |  |  |  |  | Zm00001eb317520 (142051798..142053196) | Uncharacterized protein |
|  |  |  |  |  |  |  |  | Zm00001eb317530 (142057573..142059668) | Uncharacterized protein |
|  |  |  |  |  |  |  |  | Zm00001eb317540 (142057579..142059596) | Protein capI |
|  |  |  |  |  |  |  |  | Zm00001eb317550 (142087533..142088868) | Uncharacterized protein |
|  |  |  |  |  |  |  |  | Zm00001eb317560 (142159551..142160030) | Uncharacterized protein |
|  |  |  |  |  |  |  |  | Zm00001eb317570 (142160757..142162005) | Uncharacterized protein |
|  |  |  |  |  |  |  |  | Zm00001eb317580 (142211697..142217496) | Cyclin-like domain-containing protein; Uncharacterized protein |
|  |  |  |  |  |  |  |  | Zm00001eb317590 (142217610..142236552) | Uncharacterized protein; E3 ubiquitin-protein ligase RFWD2 (RING/U-box superfamily protein) (Ubiquitin-protein ligase/ zinc ion binding protein) |
|  |  |  |  |  |  |  |  | Zm00001eb317600 (142246022..142246716) | 3'-5'-exoribonuclease family protein |
|  |  |  |  |  |  |  |  | Zm00001eb317610 (142270750..142271493) | Uncharacterized protein |
| PHK76 | Intercept | 2 | 113699825 | 115964236 | 114601670 | 6.911921154 | 41 |  |  |
|  |  |  |  |  |  |  |  | Zm00001eb088940 (113699727..113714291) | Protein transport protein Sec24-like |
|  |  |  |  |  |  |  |  | Zm00001eb088950 (113764309..113766371) | Flavin-containing monooxygenase |
|  |  |  |  |  |  |  |  | Zm00001eb088960 (113872016..113880850) | 3'-5' exonuclease domain-containing protein; Werner Syndrome-like exonuclease |
|  |  |  |  |  |  |  |  | Zm00001eb088970 (113975628..113979164) | Uncharacterized protein |
|  |  |  |  |  |  |  |  | Zm00001eb088980 (113983740..113984867) | RRM domain-containing protein |
|  |  |  |  |  |  |  |  | Zm00001eb088990 (114029458..114032129) | GRAM domain-containing protein |
|  |  |  |  |  |  |  |  | Zm00001eb089000 (114032164..114032680) | Pentatricopeptide repeat-containing protein |
|  |  |  |  |  |  |  |  | Zm00001eb089010 (114053562..114057510) | Polyketide cyclase/dehydrase and lipid transport superfamily protein |
|  |  |  |  |  |  |  |  | Zm00001eb089020 (114126581..114130435) | Pyruvate dehydrogenase E1 component subunit alpha |
|  |  |  |  |  |  |  |  | Zm00001eb089030 (114253834..114254592) | Uncharacterized protein |
|  |  |  |  |  |  |  |  | Zm00001eb089060 (114363726..114366968) | Serine/arginine-rich splicing factor RS31A |
|  |  |  |  |  |  |  |  | Zm00001eb089070 (114436807..114441147) | "Fe-S cluster assembly factor HCF101, chloroplastic; Fe-S cluster assembly factor HCF101 chloroplastic; Gamma-butyrobetaine hydroxylase-like N-terminal domain-containing protein; MIP18 family-like domain-containing protein" |
|  |  |  |  |  |  |  |  | Zm00001eb089080 (114517779..114519164) | Calcium ion binding protein; EF-hand domain-containing protein |
|  |  |  |  |  |  |  |  | Zm00001eb089090 (114559733..114560620) | Transmembrane-like protein |
|  |  |  |  |  |  |  |  | Zm00001eb089100 (114561277..114562516) | Protein SRC2-like protein |
|  |  |  |  |  |  |  |  | Zm00001eb089110 (114599617..114602053) | Alpha-humulene/(-)-(E)-beta-caryophyllene synthase |
|  |  |  |  |  |  |  |  | Zm00001eb089120 (114607026..114607619) | Aldose 1-epimerase |
|  |  |  |  |  |  |  |  | Zm00001eb089130 (114665157..114667501) | Cytochrome P450 71D7; Uncharacterized protein |
|  |  |  |  |  |  |  |  | Zm00001eb089160 (114736883..114741640) | Uncharacterized protein ycf23 |
|  |  |  |  |  |  |  |  | Zm00001eb089170 (Zm00001eb089170) | Aminotransferase class I/classII domain-containing protein |
|  |  |  |  |  |  |  |  | Zm00001eb089180 (114824707..114827398) | Long chain base biosynthesis protein 1a |
|  |  |  |  |  |  |  |  | Zm00001eb089190 (114836414..114837903) | HXXXD-type acyl-transferase family protein |
|  |  |  |  |  |  |  |  | Zm00001eb089200 (114970096..114971613) | Uncharacterized protein |
|  |  |  |  |  |  |  |  | Zm00001eb089210 (115078302..115084297) | Uncharacterized protein |
|  |  |  |  |  |  |  |  | Zm00001eb089220 (115139474..115142549) | Uncharacterized protein |
|  |  |  |  |  |  |  |  | Zm00001eb089230 (115169460..115174391) | WEB family protein |
|  |  |  |  |  |  |  |  | Zm00001eb089240 (115175930..115176403) | Uncharacterized protein |
|  |  |  |  |  |  |  |  | Zm00001eb089250 (115227780..115230049) | C2H2-type domain-containing protein; C2H2-like zinc finger protein |
|  |  |  |  |  |  |  |  | Zm00001eb089260 (115230384..115235482) | D111/G-patch domain-containing protein |
|  |  |  |  |  |  |  |  | Zm00001eb089270 (115238862..115239505) | DUF676 domain-containing protein |
|  |  |  |  |  |  |  |  | Zm00001eb089280 (115328527..115328829) | Uncharacterized protein |
|  |  |  |  |  |  |  |  | Zm00001eb089290 (115352529..115354691) | Protein kinase domain-containing protein |
|  |  |  |  |  |  |  |  | Zm00001eb089300 (115355845..115357030) | ARM repeat superfamily protein |
|  |  |  |  |  |  |  |  | Zm00001eb089310 (115357056..115357442) | Uncharacterized protein |
|  |  |  |  |  |  |  |  | Zm00001eb089330 (115437481..115441375) | ABC transporter family G domain-containing protein |
|  |  |  |  |  |  |  |  | Zm00001eb089340 (115650244..115652482) | Cytochrome P450 71D7 |
|  |  |  |  |  |  |  |  | Zm00001eb089350 (115652111..115652446) | Uncharacterized protein |
|  |  |  |  |  |  |  |  | Zm00001eb089360 (115899637..115902502) | Eudesmanediol synthase (ZmEDS) (EC 4.2.3.197) (Terpene synthase 17) (Terpene synthase 7); Uncharacterized protein |
|  |  |  |  |  |  |  |  | Zm00001eb089370 (115928437..115929308) | Alpha/beta-Hydrolases superfamily protein |
|  |  |  |  |  |  |  |  | Zm00001eb089380 (115951171..115956690) | E2F-DP transcription factor (Transcription factor E2FC) |
|  |  |  |  |  |  |  |  | Zm00001eb089390 (115958214..115966591) | sulfate adenylyltransferase (EC 2.7.7.4) |
| PHK76 | Intercept | 2 | 117129546 | 121719422 | 120919436 | 6.760166946 | 50 |  |  |
|  |  |  |  |  |  |  |  | Zm00001eb089560 (117129112..117129988) | Uncharacterized protein |
|  |  |  |  |  |  |  |  | Zm00001eb089570 (117150758..117151626) | Uncharacterized protein |
|  |  |  |  |  |  |  |  | Zm00001eb089580 (117151993..117162433) | C3H transcription factor (Zinc finger CCCH domain-containing protein 24) |
|  |  |  |  |  |  |  |  | Zm00001eb089590 (117262167..117266429) | Rhomboid-like protein 19 |
|  |  |  |  |  |  |  |  | Zm00001eb089600 (117266800..117270208) | Glycosyltransferases |
|  |  |  |  |  |  |  |  | Zm00001eb089610 (117279116..117281088) | RING-type domain-containing protein |
|  |  |  |  |  |  |  |  | Zm00001eb089620 (117320949..117323680) | Uncharacterized protein |
|  |  |  |  |  |  |  |  | Zm00001eb089630 (117378105..117381815) | OSJNBa0089E12.13-like protein; DUF4220 domain-containing protein |
|  |  |  |  |  |  |  |  | Zm00001eb089650 (117456634..117462954) | Peptidase S24/S26A/S26B/S26C family protein |
|  |  |  |  |  |  |  |  | Zm00001eb089660 (117558797..117564100) | Phosphoglycerate mutase-like protein |
|  |  |  |  |  |  |  |  | Zm00001eb089670 (117640271..117644752) | Zinc finger (C3HC4-type RING finger) family protein |
|  |  |  |  |  |  |  |  | Zm00001eb089680 (117644462..117648341) | ELMO domain-containing protein; ELMO domain-containing protein 2 (ELMO/CED-12 family protein) |
|  |  |  |  |  |  |  |  | Zm00001eb089700 (117797645..117799833) | Cytochrome P450 family 93 subfamily D polypeptide 1 |
|  |  |  |  |  |  |  |  | Zm00001eb089710 (117908813..117911989) | Uncharacterized protein |
|  |  |  |  |  |  |  |  | Zm00001eb089720 (117944307..117949137) | Formin-like protein |
|  |  |  |  |  |  |  |  | Zm00001eb089730 (117968881..117972282) | Uncharacterized protein |
|  |  |  |  |  |  |  |  | Zm00001eb089740 (118085128..118109868) | Leucine-rich repeat protein kinase family protein; Protein kinase domain-containing protein |
|  |  |  |  |  |  |  |  | Zm00001eb089770 (118169104..118171350) | Long chain base biosynthesis protein 1 |
|  |  |  |  |  |  |  |  | Zm00001eb089790 (118189866..118200007) | Mitochondrial uncoupling protein 2; Mitochondrial uncoupling protein 1 |
|  |  |  |  |  |  |  |  | Zm00001eb089800 (118204227..118211712) | Aconitate hydratase (Aconitase) |
|  |  |  |  |  |  |  |  | Zm00001eb089820 (118256155..118257451) | Uncharacterized protein |
|  |  |  |  |  |  |  |  | Zm00001eb089840 (118391490..118413535) | DUF6598 domain-containing protein |
|  |  |  |  |  |  |  |  | Zm00001eb089850 (118644223..118645844) | Uncharacterized protein |
|  |  |  |  |  |  |  |  | Zm00001eb089860 (118683556..118699323) | DUF6598 domain-containing protein |
|  |  |  |  |  |  |  |  | Zm00001eb089870 (118959463..118968503) | F-box domain-containing protein |
|  |  |  |  |  |  |  |  | Zm00001eb089880 (119080636..119081650) | Uncharacterized protein |
|  |  |  |  |  |  |  |  | Zm00001eb089890 (119086309..119086701) | Uncharacterized protein |
|  |  |  |  |  |  |  |  | Zm00001eb089900 (119282383..119283210) | SAUR-like auxin-responsive protein family |
|  |  |  |  |  |  |  |  | Zm00001eb089910 (119293791..119294312) | BHLH domain-containing protein |
|  |  |  |  |  |  |  |  | Zm00001eb089930 (119547636..119548928) | rRNA N-glycosylase |
|  |  |  |  |  |  |  |  | Zm00001eb089940 (119630970..119631890) | Uncharacterized protein |
|  |  |  |  |  |  |  |  | Zm00001eb089950 (119664507..119665238) | Uncharacterized protein |
|  |  |  |  |  |  |  |  | Zm00001eb089960 (120079466..120083618) | Beta-expansin 1a (Expansin-B4) |
|  |  |  |  |  |  |  |  | Zm00001eb089970 (120227368..120229455) | Uncharacterized protein |
|  |  |  |  |  |  |  |  | Zm00001eb089980 (120680500..120684820) | 60 kDa jasmonate-induced protein; Expansin-like CBD domain-containing protein; DUF6598 domain-containing protein |
|  |  |  |  |  |  |  |  | Zm00001eb089990 (120714562..120716970) |  |
|  |  |  |  |  |  |  |  | Zm00001eb090000 (120850367..120851025) | Uncharacterized protein |
|  |  |  |  |  |  |  |  | Zm00001eb090010 (120893046..120893493) | Uncharacterized protein |
|  |  |  |  |  |  |  |  | Zm00001eb090020 (120893613..120894283) | Myb/SANT-like domain-containing protein |
|  |  |  |  |  |  |  |  | Zm00001eb090030 (120908328..120934745) | Uncharacterized protein |
|  |  |  |  |  |  |  |  | Zm00001eb090050 (121351851..121352528) | Uncharacterized protein |
|  |  |  |  |  |  |  |  | Zm00001eb090060 (121369977..121370556) | Uncharacterized protein |
|  |  |  |  |  |  |  |  | Zm00001eb090070 (121373897..121376581) | Uncharacterized protein |
|  |  |  |  |  |  |  |  | Zm00001eb090080 (121415955..121416497) | Uncharacterized protein |
|  |  |  |  |  |  |  |  | Zm00001eb090090 (121417936..121427213) | TEA domain-containing protein |
|  |  |  |  |  |  |  |  | Zm00001eb090100 (121538441..121541983) | Uncharacterized protein |
|  |  |  |  |  |  |  |  | Zm00001eb090110 (121664131..121665331) | Acyl-CoA N-acyltransferase (NAT) superfamily protein (GNAT transcription factor) |
|  |  |  |  |  |  |  |  | Zm00001eb090120 (121702530..121702925) | N-acetyltransferase domain-containing protein |
|  |  |  |  |  |  |  |  | Zm00001eb090130 (121702953..121719902) | Uncharacterized protein |
|  |  |  |  |  |  |  |  | Zm00001eb090160 (121851020..121852010) | Bifunctional inhibitor/plant lipid transfer protein/seed storage helical domain-containing protein |
| PHK76 | Intercept | 7 | 142212068 | 142272843 | 142228900 | 6.27107602 | 5 |  |  |
|  |  |  |  |  |  |  |  | Zm00001eb317580 (142211697..142217496) | Cyclin-like domain-containing protein; Uncharacterized protein |
|  |  |  |  |  |  |  |  | Zm00001eb317590 (142217610..142236552) | ncharacterized protein; E3 ubiquitin-protein ligase RFWD2 (RING/U-box superfamily protein) (Ubiquitin-protein ligase/ zinc ion binding protein) |
|  |  |  |  |  |  |  |  | Zm00001eb317600 (142246022..142246716) | 3'-5'-exoribonuclease family protein |
|  |  |  |  |  |  |  |  | Zm00001eb317610 (142270750..142271493) | Uncharacterized protein |
|  |  |  |  |  |  |  |  | Zm00001eb317630 (142271973..142276574) | Calcium-dependent protein kinase 14 |
| PHP02 | Slope | 6 | 171965401 | 171966021 | 171965401 | 5.457138481 | 1 |  |  |
|  |  |  |  |  |  |  |  | Zm00001eb293730 (171965158..171966322) | Uncharacterized protein |
| PHP02 | Intercept | 9 | 21040087 | 21345635 | 21102505 | 5.76575703 | 15 |  |  |
|  |  |  |  |  |  |  |  | Zm00001eb376840 (21039872..21045940) | Uncharacterized protein; NB-ARC domain-containing protein |
|  |  |  |  |  |  |  |  | Zm00001eb376850 (21048095..21054027) | Saposin B-type domain-containing protein |
|  |  |  |  |  |  |  |  | Zm00001eb376860 (21095830..21097630) | HXXXD-type acyl-transferase family protein |
|  |  |  |  |  |  |  |  | Zm00001eb376870 (21098480..21099308) | Ubiquitin-like domain-containing protein |
|  |  |  |  |  |  |  |  | Zm00001eb376880 (21099772..21102568) | Trafficking protein particle complex subunit |
|  |  |  |  |  |  |  |  | Zm00001eb376890 (21119954..21121937) | Tryptophan synthase beta chain-like PALP domain-containing protein; Pyridoxal-5'-phosphate-dependent enzyme family protein |
|  |  |  |  |  |  |  |  | Zm00001eb376900 (21124514..21127951) | Tryptophan synthase beta chain-like PALP domain-containing protein |
|  |  |  |  |  |  |  |  | Zm00001eb376910 (21127836..21130893) | Lactoylglutathione lyase / glyoxalase I family protein |
|  |  |  |  |  |  |  |  | Zm00001eb376920 (21154373..21155416) | DUF295 domain-containing protein |
|  |  |  |  |  |  |  |  | Zm00001eb376930 (21156037..21168630) | Pyridoxal-5'-phosphate-dependent enzyme family protein |
|  |  |  |  |  |  |  |  | Zm00001eb376940 (21158231..21160894) |  |
|  |  |  |  |  |  |  |  | Zm00001eb376950 (21165349..21166392) | DUF295 domain-containing protein |
|  |  |  |  |  |  |  |  | Zm00001eb376960 (21186644..21187687) | DUF295 domain-containing protein |
|  |  |  |  |  |  |  |  | Zm00001eb376970 (21188308..21192571) |  |
|  |  |  |  |  |  |  |  | Zm00001eb376980 (21194827..21198952) | Nucleosome assembly protein 1 |
| PHZ51 | Slope | 7 | 147716439 | 149566039 | 148936212 | 6.48169504 | 69 |  |  |
|  |  |  |  |  |  |  |  | Zm00001eb318990 (147715244..147717880) | Ubiquitin-like domain-containing protein; BAG family molecular chaperone regulator 1; Protein binding protein |
|  |  |  |  |  |  |  |  | Zm00001eb319000 (147731360..147733872) | F-box protein PP2-A13 |
|  |  |  |  |  |  |  |  | Zm00001eb319010 (147773749..147778434) | RING-type domain-containing protein; Protein binding protein (RING/U-box superfamily protein) |
|  |  |  |  |  |  |  |  | Zm00001eb319020 (147779934..147780802) | YY1 protein |
|  |  |  |  |  |  |  |  | Zm00001eb319030 (147780699..147790384) | UBX domain-containing protein; Plant UBX domain-containing protein 8 |
|  |  |  |  |  |  |  |  | Zm00001eb319040 (147791106..147792420) | "Glucan endo-1, 3-beta-D-glucosidase" |
|  |  |  |  |  |  |  |  | Zm00001eb319050 (147830796..147833634) | RHOMBOID-like protein (EC 3.4.21.-) (Rhomboid-like protein) |
|  |  |  |  |  |  |  |  | Zm00001eb319060 (147842854..147845360) | Protein ROOT PRIMORDIUM DEFECTIVE 1 |
|  |  |  |  |  |  |  |  | Zm00001eb319080 (147874032..147874803) |  |
|  |  |  |  |  |  |  |  | Zm00001eb319090 (147881484..147888019) | Homeodomain leucine zipper family IV protein |
|  |  |  |  |  |  |  |  | Zm00001eb319100 (147889758..147891284) | SAUR-like auxin-responsive protein family |
|  |  |  |  |  |  |  |  | Zm00001eb319110 (147945930..147951379) | Ypt/Rab-GAP domain of gyp1p superfamily protein |
|  |  |  |  |  |  |  |  | Zm00001eb319120 (147954681..147956941) | Uncharacterized protein; MnmG N-terminal domain-containing protein |
|  |  |  |  |  |  |  |  | Zm00001eb319130 (147957385..147963651) | Protein TILLER ANGLE CONTROL 1 |
|  |  |  |  |  |  |  |  | Zm00001eb319140 (147973341..147975292) | Uncharacterized protein |
|  |  |  |  |  |  |  |  | Zm00001eb319150 (147976312..147980256) | DUF295 domain-containing protein |
|  |  |  |  |  |  |  |  | Zm00001eb319160 (148000313..148000849) | Glutaredoxin domain-containing protein |
|  |  |  |  |  |  |  |  | Zm00001eb319170 (148001961..148003234) | ALA-interacting subunit |
|  |  |  |  |  |  |  |  | Zm00001eb319180 (148008660..148009182) |  |
|  |  |  |  |  |  |  |  | Zm00001eb319210 (148019596..148023694) | Rhodanese domain-containing protein; Rhodanese-like domain containing protein |
|  |  |  |  |  |  |  |  | Zm00001eb319220 (148023190..148026200) | Ras-related protein RABA3 (Ras-related protein Rab11A) |
|  |  |  |  |  |  |  |  | Zm00001eb319230 (148027449..148035225) | DOG1 domain-containing protein |
|  |  |  |  |  |  |  |  | Zm00001eb319240 (148039685..148042169) | LRAT domain-containing protein; NC domain-containing protein-related |
|  |  |  |  |  |  |  |  | Zm00001eb319250 (148043140..148046414) | UDP-glucose 4-epimerase |
|  |  |  |  |  |  |  |  | Zm00001eb319260 (148068570..148070541) | Uncharacterized protein |
|  |  |  |  |  |  |  |  | Zm00001eb319270 (148068871..148070402) | Transmembrane protein |
|  |  |  |  |  |  |  |  | Zm00001eb319280 (148076420..148086870) | Glutamyl-tRNA reductase-binding protein chloroplastic |
|  |  |  |  |  |  |  |  | Zm00001eb319300 (148244032..148252180) | Cysteine-rich receptor-like protein kinase 25 |
|  |  |  |  |  |  |  |  | Zm00001eb319310 (148307133..148308826) | EndoU domain-containing protein |
|  |  |  |  |  |  |  |  | Zm00001eb319320 (148309926..148313207) | Uncharacterized protein |
|  |  |  |  |  |  |  |  | Zm00001eb319330 (148314919..148345199) | ATP binding protein (Chloroplast sensor kinase chloroplastic) |
|  |  |  |  |  |  |  |  | Zm00001eb319340 (148364274..148368417) | Thioredoxin domain-containing protein |
|  |  |  |  |  |  |  |  | Zm00001eb319350 (148373290..148375381) | Heat stress transcription factor B-2b |
|  |  |  |  |  |  |  |  | Zm00001eb319360 (148452225..148453017) | Rx N-terminal domain-containing protein |
|  |  |  |  |  |  |  |  | Zm00001eb319370 (148455019..148456037) | BON1-associated protein 2 |
|  |  |  |  |  |  |  |  | Zm00001eb319380 (148531796..148542267) | Mediator of RNA polymerase II transcription subunit 10 (Mediator complex subunit 10) |
|  |  |  |  |  |  |  |  | Zm00001eb319390 (148545101..148546761) | Homeobox-leucine zipper protein (HD-ZIP protein) (Homeodomain transcription factor) |
|  |  |  |  |  |  |  |  | Zm00001eb319400 (148556853..148560430) | F-box domain-containing protein |
|  |  |  |  |  |  |  |  | Zm00001eb319410 (148563965..148571077) | Kinesin-like protein KIN-7G; Kinesin motor domain-containing protein |
|  |  |  |  |  |  |  |  | Zm00001eb319420 (148567285..148568039) | Uncharacterized protein |
|  |  |  |  |  |  |  |  | Zm00001eb319430 (148676802..148677260) | Uncharacterized protein |
|  |  |  |  |  |  |  |  | Zm00001eb319440 (148677305..148677724) | Uncharacterized protein |
|  |  |  |  |  |  |  |  | Zm00001eb319450 (148745038..148745394) | Protein TIFY (Jasmonate ZIM domain-containing protein) |
|  |  |  |  |  |  |  |  | Zm00001eb319460 (148842830..148847329) | B box-type domain-containing protein; B-box zinc finger protein 19 (Orphans transcription factor) (Salt tolerance-like protein) |
|  |  |  |  |  |  |  |  | Zm00001eb319470 (148935616..148937490) | Auxin-responsive protein |
|  |  |  |  |  |  |  |  | Zm00001eb319480 (148941882..148945639) | cellulase (EC 3.2.1.4); Endoglucanase (EC 3.2.1.4) |
|  |  |  |  |  |  |  |  | Zm00001eb319490 (148959266..148959892) | Late embryogenesis abundant protein |
|  |  |  |  |  |  |  |  | Zm00001eb319500 (148972124..148975338) | Uncharacterized protein |
|  |  |  |  |  |  |  |  | Zm00001eb319510 (149013325..149018005) | Oxidoreductase-like domain-containing protein |
|  |  |  |  |  |  |  |  | Zm00001eb319520 (149013576..149018003) | Subtilisin-like protease SBT3.9; Uncharacterized protein |
|  |  |  |  |  |  |  |  | Zm00001eb319530 (149063306..149065181) | Protein SHI RELATED SEQUENCE 1 (SRS transcription factor) |
|  |  |  |  |  |  |  |  | Zm00001eb319540 (149162356..149164248) | Protein kinase domain-containing protein |
|  |  |  |  |  |  |  |  | Zm00001eb319550 (149170577..149175477) | Hexosyltransferase |
|  |  |  |  |  |  |  |  | Zm00001eb319560 (149181028..149182975) | Senescence-inducible chloroplast stay-green protein 1 |
|  |  |  |  |  |  |  |  | Zm00001eb319570 (149210004..149211074) | Late embryogenesis abundant (LEA) hydroxyproline-rich glycoprotein family |
|  |  |  |  |  |  |  |  | Zm00001eb319580 (149271489..149272289) | Protein SHI RELATED SEQUENCE 1 |
|  |  |  |  |  |  |  |  | Zm00001eb319590 (149275677..149281853) | Two-component response regulator-like APRR9 |
|  |  |  |  |  |  |  |  | Zm00001eb319600 (149338212..149349010) | "Pentatricopeptide repeat-containing protein mitochondrial (Peptidase S8 and S53, subtilisin, kexin, sedolisin)" |
|  |  |  |  |  |  |  |  | Zm00001eb319610 (149349585..149368712) | DNA photolyase; AB hydrolase-1 domain-containing protein; DNA photolyase (Deoxyribodipyrimidine photolyase family protein) |
|  |  |  |  |  |  |  |  | Zm00001eb319620 (149364659..149375517) | Protein ODORANT1 |
|  |  |  |  |  |  |  |  | Zm00001eb319630 (149376706..149401753) | Pantothenate kinase 2 (EC 2.7.1.33); Damage-control phosphatase ARMT1-like metal-binding domain-containing protein |
|  |  |  |  |  |  |  |  | Zm00001eb319640 (149392346..149393434) | Uncharacterized protein |
|  |  |  |  |  |  |  |  | Zm00001eb319650 (149399852..149401753) | Putative glucan endo-13-beta-glucosidase BG4 |
|  |  |  |  |  |  |  |  | Zm00001eb319660 (149432598..149436424) | Phosphatase DCR2; Calcineurin-like phosphoesterase domain-containing protein |
|  |  |  |  |  |  |  |  | Zm00001eb319670 (149437446..149438323) | Histone H2A |
|  |  |  |  |  |  |  |  | Zm00001eb319680 (149474851..149483722) | "Lon protease homolog 2, peroxisomal" |
|  |  |  |  |  |  |  |  | Zm00001eb319700 (149542446..149547197) | Protein kinase domain-containing protein; Putative receptor-like protein kinase |
|  |  |  |  |  |  |  |  | Zm00001eb319710 (149558075..149563530) | Endoglucanase |
|  |  |  |  |  |  |  |  | Zm00001eb319720 (149564247..149567044) | Seipin-2; Uncharacterized protein |
| PHZ51 | Intercept | 7 | 147650397 | 150017427 | 148936212 | 7.861814653 | 84 |  |  |
|  |  |  |  |  |  |  |  | Zm00001eb318940 (147650115..147652333) | Protein DETOXIFICATION (Multidrug and toxic compound extrusion protein) |
|  |  |  |  |  |  |  |  | Zm00001eb318950 (147651253..147651627) | Uncharacterized protein |
|  |  |  |  |  |  |  |  | Zm00001eb318970 (147684571..147686411) | Glycosyltransferase |
|  |  |  |  |  |  |  |  | Zm00001eb318980 (147703570..147707267) | Protein kinase domain-containing protein |
|  |  |  |  |  |  |  |  | Zm00001eb318990 (147715244..147717880) | Ubiquitin-like domain-containing protein; BAG family molecular chaperone regulator 1; Protein binding protein |
|  |  |  |  |  |  |  |  | Zm00001eb319000 (147731360..147733872) | F-box protein PP2-A13 |
|  |  |  |  |  |  |  |  | Zm00001eb319010 (147773749..147778434) | RING-type domain-containing protein; Protein binding protein (RING/U-box superfamily protein) |
|  |  |  |  |  |  |  |  | Zm00001eb319020 (147779934..147780802) | YY1 protein |
|  |  |  |  |  |  |  |  | Zm00001eb319030 (147780699..147790384) | UBX domain-containing protein; Plant UBX domain-containing protein 8 |
|  |  |  |  |  |  |  |  | Zm00001eb319040 (147791106..147792420) | "Glucan endo-1 3-beta-D-glucosidase" |
|  |  |  |  |  |  |  |  | Zm00001eb319050 (147830796..147833634) | RHOMBOID-like protein (EC 3.4.21.-) (Rhomboid-like protein) |
|  |  |  |  |  |  |  |  | Zm00001eb319060 (147842854..147845360) | Protein ROOT PRIMORDIUM DEFECTIVE 1 |
|  |  |  |  |  |  |  |  | Zm00001eb319080 (147874032..147874803) |  |
|  |  |  |  |  |  |  |  | Zm00001eb319090 (147881484..147888019) | Homeodomain leucine zipper family IV protein |
|  |  |  |  |  |  |  |  | Zm00001eb319100 (147889758..147891284) | SAUR-like auxin-responsive protein family |
|  |  |  |  |  |  |  |  | Zm00001eb319110 (147945930..147951379) | Ypt/Rab-GAP domain of gyp1p superfamily protein |
|  |  |  |  |  |  |  |  | Zm00001eb319120 (147954681..147956941) | Uncharacterized protein; MnmG N-terminal domain-containing protein |
|  |  |  |  |  |  |  |  | Zm00001eb319130 (147957385..147963651) | Protein TILLER ANGLE CONTROL 1 |
|  |  |  |  |  |  |  |  | Zm00001eb319140 (147973341..147975292) | Uncharacterized protein |
|  |  |  |  |  |  |  |  | Zm00001eb319150 (147976312..147980256) | DUF295 domain-containing protein |
|  |  |  |  |  |  |  |  | Zm00001eb319160 (148000313..148000849) | Glutaredoxin domain-containing protein |
|  |  |  |  |  |  |  |  | Zm00001eb319170 (148001961..148003234) | ALA-interacting subunit |
|  |  |  |  |  |  |  |  | Zm00001eb319180 (148008660..148009182) |  |
|  |  |  |  |  |  |  |  | Zm00001eb319210 (148019596..148023694) | Rhodanese domain-containing protein; Rhodanese-like domain containing protein |
|  |  |  |  |  |  |  |  | Zm00001eb319220 (148023190..148026200) | Ras-related protein RABA3 (Ras-related protein Rab11A) |
|  |  |  |  |  |  |  |  | Zm00001eb319230 (148027449..148035225) | DOG1 domain-containing protein |
|  |  |  |  |  |  |  |  | Zm00001eb319240 (148039685..148042169) | LRAT domain-containing protein; NC domain-containing protein-related |
|  |  |  |  |  |  |  |  | Zm00001eb319250 (148043140..148046414) | UDP-glucose 4-epimerase |
|  |  |  |  |  |  |  |  | Zm00001eb319260 (148068570..148070541) | Uncharacterized protein |
|  |  |  |  |  |  |  |  | Zm00001eb319270 (148068871..148070402) | Transmembrane protein |
|  |  |  |  |  |  |  |  | Zm00001eb319280 (148076420..148086870) | Glutamyl-tRNA reductase-binding protein chloroplastic |
|  |  |  |  |  |  |  |  | Zm00001eb319300 (148244032..148252180) | Cysteine-rich receptor-like protein kinase 25 |
|  |  |  |  |  |  |  |  | Zm00001eb319310 (148307133..148308826) | EndoU domain-containing protein |
|  |  |  |  |  |  |  |  | Zm00001eb319320 (148309926..148313207) | Uncharacterized protein |
|  |  |  |  |  |  |  |  | Zm00001eb319330 (148314919..148345199) | ATP binding protein (Chloroplast sensor kinase chloroplastic) |
|  |  |  |  |  |  |  |  | Zm00001eb319340 (148364274..148368417) | Thioredoxin domain-containing protein |
|  |  |  |  |  |  |  |  | Zm00001eb319350 (148373290..148375381) | Heat stress transcription factor B-2b |
|  |  |  |  |  |  |  |  | Zm00001eb319360 (148452225..148453017) | Rx N-terminal domain-containing protein |
|  |  |  |  |  |  |  |  | Zm00001eb319370 (148455019..148456037) | BON1-associated protein 2 |
|  |  |  |  |  |  |  |  | Zm00001eb319380 (148531796..148542267) | Mediator of RNA polymerase II transcription subunit 10 (Mediator complex subunit 10) |
|  |  |  |  |  |  |  |  | Zm00001eb319390 (148545101..148546761) | Homeobox-leucine zipper protein (HD-ZIP protein) (Homeodomain transcription factor) |
|  |  |  |  |  |  |  |  | Zm00001eb319400 (148556853..148560430) | F-box domain-containing protein |
|  |  |  |  |  |  |  |  | Zm00001eb319410 (148563965..148571077) | Kinesin-like protein KIN-7G; Kinesin motor domain-containing protein |
|  |  |  |  |  |  |  |  | Zm00001eb319420 (148567285..148568039) | Uncharacterized protein |
|  |  |  |  |  |  |  |  | Zm00001eb319430 (148676802..148677260) | Uncharacterized protein |
|  |  |  |  |  |  |  |  | Zm00001eb319440 (148677305..148677724) | Uncharacterized protein |
|  |  |  |  |  |  |  |  | Zm00001eb319450 (148745038..148745394) | Protein TIFY (Jasmonate ZIM domain-containing protein) |
|  |  |  |  |  |  |  |  | Zm00001eb319460 (148842830..148847329) | B box-type domain-containing protein; B-box zinc finger protein 19 (Orphans transcription factor) (Salt tolerance-like protein) |
|  |  |  |  |  |  |  |  | Zm00001eb319470 (148935616..148937490) | Auxin-responsive protein |
|  |  |  |  |  |  |  |  | Zm00001eb319480 (148941882..148945639) | cellulase (EC 3.2.1.4); Endoglucanase (EC 3.2.1.4) |
|  |  |  |  |  |  |  |  | Zm00001eb319490 (148959266..148959892) | Late embryogenesis abundant protein |
|  |  |  |  |  |  |  |  | Zm00001eb319500 (148972124..148975338) | Uncharacterized protein |
|  |  |  |  |  |  |  |  | Zm00001eb319510 (149013325..149018005) | Oxidoreductase-like domain-containing protein |
|  |  |  |  |  |  |  |  | Zm00001eb319520 (149013576..149018003) | Subtilisin-like protease SBT3.9; Uncharacterized protein |
|  |  |  |  |  |  |  |  | Zm00001eb319530 (149063306..149065181) | Protein SHI RELATED SEQUENCE 1 (SRS transcription factor) |
|  |  |  |  |  |  |  |  | Zm00001eb319540 (149162356..149164248) | Protein kinase domain-containing protein |
|  |  |  |  |  |  |  |  | Zm00001eb319550 (149170577..149175477) | Hexosyltransferase |
|  |  |  |  |  |  |  |  | Zm00001eb319560 (149181028..149182975) | Senescence-inducible chloroplast stay-green protein 1 |
|  |  |  |  |  |  |  |  | Zm00001eb319570 (149210004..149211074) | Late embryogenesis abundant (LEA) hydroxyproline-rich glycoprotein family |
|  |  |  |  |  |  |  |  | Zm00001eb319580 (149271489..149272289) | Protein SHI RELATED SEQUENCE 1 |
|  |  |  |  |  |  |  |  | Zm00001eb319600 (149338212..149349010) | "Pentatricopeptide repeat-containing protein mitochondrial (Peptidase S8 and S53 subtilisin kexin sedolisin)" |
|  |  |  |  |  |  |  |  | Zm00001eb319610 (149349585..149368712) | DNA photolyase; AB hydrolase-1 domain-containing protein; DNA photolyase (Deoxyribodipyrimidine photolyase family protein) |
|  |  |  |  |  |  |  |  | Zm00001eb319620 (149364659..149375517) | Protein ODORANT1 |
|  |  |  |  |  |  |  |  | Zm00001eb319630 (149376706..149401753) | Pantothenate kinase 2 (EC 2.7.1.33); Damage-control phosphatase ARMT1-like metal-binding domain-containing protein |
|  |  |  |  |  |  |  |  | Zm00001eb319640 (149392346..149393434) | Uncharacterized protein |
|  |  |  |  |  |  |  |  | Zm00001eb319650 (149399852..149401753) | Putative glucan endo-13-beta-glucosidase BG4 |
|  |  |  |  |  |  |  |  | Zm00001eb319660 (149432598..149436424) | Phosphatase DCR2; Calcineurin-like phosphoesterase domain-containing protein |
|  |  |  |  |  |  |  |  | Zm00001eb319670 (149437446..149438323) | Histone H2A |
|  |  |  |  |  |  |  |  | Zm00001eb319680 (149474851..149483722) | "Lon protease homolog 2 peroxisomal" |
|  |  |  |  |  |  |  |  | Zm00001eb319700 (149542446..149547197) | Protein kinase domain-containing protein; Putative receptor-like protein kinase |
|  |  |  |  |  |  |  |  | Zm00001eb319710 (149558075..149563530) | Endoglucanase |
|  |  |  |  |  |  |  |  | Zm00001eb319720 (149564247..149567044) | Seipin-2; Uncharacterized protein |
|  |  |  |  |  |  |  |  | Zm00001eb319730 (149571732..149575851) | ER lumen protein retaining receptor C28H8.4 (ER lumen protein retaining receptor family protein) |
|  |  |  |  |  |  |  |  | Zm00001eb319740 (149581376..149585809) | Protein kinase domain-containing protein |
|  |  |  |  |  |  |  |  | Zm00001eb319750 (149651961..149652350) | Uncharacterized protein |
|  |  |  |  |  |  |  |  | Zm00001eb319760 (149661999..149668277) | Cyclin-like domain-containing protein; Transcription initiation factor IIB-2 |
|  |  |  |  |  |  |  |  | Zm00001eb319770 (149671226..149675207) | Triosephosphate isomerase |
|  |  |  |  |  |  |  |  | Zm00001eb319780 (149779817..149782279) | PIN domain-like family protein; rRNA-processing protein UTP23 |
|  |  |  |  |  |  |  |  | Zm00001eb319790 (149840908..149845282) | Protein binding protein; RING-type domain-containing protein |
|  |  |  |  |  |  |  |  | Zm00001eb319800 (149906151..149907928) | "lagen type IV alpha 5" |
|  |  |  |  |  |  |  |  | Zm00001eb319810 (149908363..149909568) | RING-H2 finger protein ATL2A |
|  |  |  |  |  |  |  |  | Zm00001eb319820 (149969321..149971057) | Phosphatidylinositol-specific phospholipase C X domain-containing protein; DNA-(Apurinic or apyrimidinic site) lyase 2 |
|  |  |  |  |  |  |  |  | Zm00001eb319840 (150010081..150011091) | Uncharacterized protein |
|  |  |  |  |  |  |  |  | Zm00001eb319850 (150016595..150018597) | ornithine decarboxylase |
